# Insulin protects acinar cells during pancreatitis by preserving glycolytic ATP supply to calcium pumps

**DOI:** 10.1038/s41467-021-24506-w

**Published:** 2021-07-19

**Authors:** Jason I. E. Bruce, Rosa Sánchez-Alvarez, Maria Dolors Sans, Sarah A. Sugden, Nathan Qi, Andrew D. James, John A. Williams

**Affiliations:** 1 Division of Cancer Sciences, School of Medical Sciences, Faculty of Biology, Medicine and Health, The University of Manchester, Manchester, UK; 2 Department of Molecular and Integrative Physiology, University of Michigan, Ann Arbor, MI USA; 3 Present Address: Division of Cancer Sciences, Department of Biology, University of York, Heslington, York, UK

**Keywords:** Cell biology, Physiology, Endocrinology, Gastroenterology

## Abstract

Acute pancreatitis (AP) is serious inflammatory disease of the pancreas. Accumulating evidence links diabetes with severity of AP, suggesting that endogenous insulin may be protective. We investigated this putative protective effect of insulin during cellular and in vivo models of AP in diabetic mice (Ins2^Akita^) and Pancreatic Acinar cell-specific Conditional Insulin Receptor Knock Out mice (PACIRKO). Caerulein and palmitoleic acid (POA)/ethanol-induced pancreatitis was more severe in both Ins2^Akita^ and PACIRKO vs control mice, suggesting that endogenous insulin directly protects acinar cells in vivo. In isolated pancreatic acinar cells, insulin induced Akt-mediated phosphorylation of 6-phosphofructo-2-kinase/fructose-2,6-biphosphatase 2 (PFKFB2) which upregulated glycolysis thereby preventing POA-induced ATP depletion, inhibition of the ATP-dependent plasma membrane Ca^2+^ ATPase (PMCA) and cytotoxic Ca^2+^ overload. These data provide the first mechanistic link between diabetes and severity of AP and suggest that phosphorylation of PFKFB2 may represent a potential therapeutic strategy for treatment of AP.

## Abstract (web summary)

Acute pancreatitis is a serious inflammatory disease, which is more severe in diabetic mice. Here the authors use mice lacking pancreatic acinar cell insulin receptors to show that this may be because insulin preserves glycolytic energy supply in acinar cell during pancreatitis, which prevents cytotoxic calcium overload and cell death.

## Introduction

Pancreatitis is an inflammatory disease of the exocrine pancreas which in most cases is initiated by bile acid reflux from gall stones or fatty acid/ethanol metabolites from excessive alcohol and fat consumption^1^. Severe acute pancreatitis (SAP) is characterised by pancreatic necrosis and multiple organ failure, which increases mortality and prolongs critical care occupancy. In the USA SAP accounts for 1.4 million hospital days at a cost of $2.6 billion every year^2^. There is no imminent cure and treatment is restricted to nutritional support and fluid resuscitation^3,4^. Clearly a radical approach to the treatment of SAP is urgently required to improve survival and reduce critical care occupancy. Impaired metabolism and cytotoxic Ca^2+^ ([Ca^2+^]_i_) overload in pancreatic acinar cells are central events regardless of the causative factor^1^. Metabolism and [Ca^2+^]_i_ are linked by the ATP-driven plasma membrane Ca^2+^ ATPase (PMCA), which prevents cytotoxic Ca^2+^ overload. Therefore, restoration of acinar cell metabolism and protection of PMCA represents an attractive therapeutic strategy, which might be achieved by insulin.

Using cellular models of pancreatitis our previous studies revealed that insulin protects acinar cells from cellular injury^5,6^. However, it is unclear from these studies in isolated pancreatic acinar cells whether endogenous insulin release has any protective effect in vivo and the specific molecular mechanism remains unknown. Although pancreatitis can lead to type 3c diabetes, due to collateral pancreatic β-cell injury and the loss of insulin secretion^7,8^, emerging evidence from clinical^9–14^ and animal studies^15–17^ suggest that diabetes increases the severity of AP and thus endogenous insulin may be protective.

However, it is very difficult to separate the systemic effects of hyperglycaemia, reduced insulin secretion (type-1 diabetes) or reduced insulin sensitivity (type-2 diabetes), from any putative direct protective effects of insulin on pancreatic acinar cells. This was achieved in the current study by inducing experimental AP in Pancreatic Acinar cell-specific Conditional Insulin Receptor Knock Out mice (PACIRKO) in which insulin receptors (IRs) were deleted specifically in acinar cells using a tamoxifen-induced Cre-lox-based gene deletion system^18^. Results show that pancreatitis was more severe in both Ins2^Akita^ mice (type-1 diabetic mouse model) and in PACIRKO mice. This was due to a loss of insulin-induced, Akt-mediated phosphorylation of the key glycolytic enzyme, 6-phosphofructo-2-kinase/fructose-2,6-bisphosphatase 2 (PFKFB2) in pancreatic acinar cells. This is sufficient to drive glycolytic flux and maintain the glycolytic ATP supply to the plasma membrane Ca^2+^ pumps (PMCA), thereby preventing cytotoxic Ca^2+^ overload and necrotic cell death even in the face of impaired mitochondrial function. Collectively, this provides a strong mechanistic link between diabetes and the severity of acute pancreatitis and suggests that the phosphorylation of PFKFB2, by insulin or insulin-mimetics, and the preservation of acinar cell ATP, may represent a potential therapeutic strategy for the treatment of AP.

## Results

### Pancreatitis is worse in diabetic Ins2^Akita^ mice

**Fig. 1:**
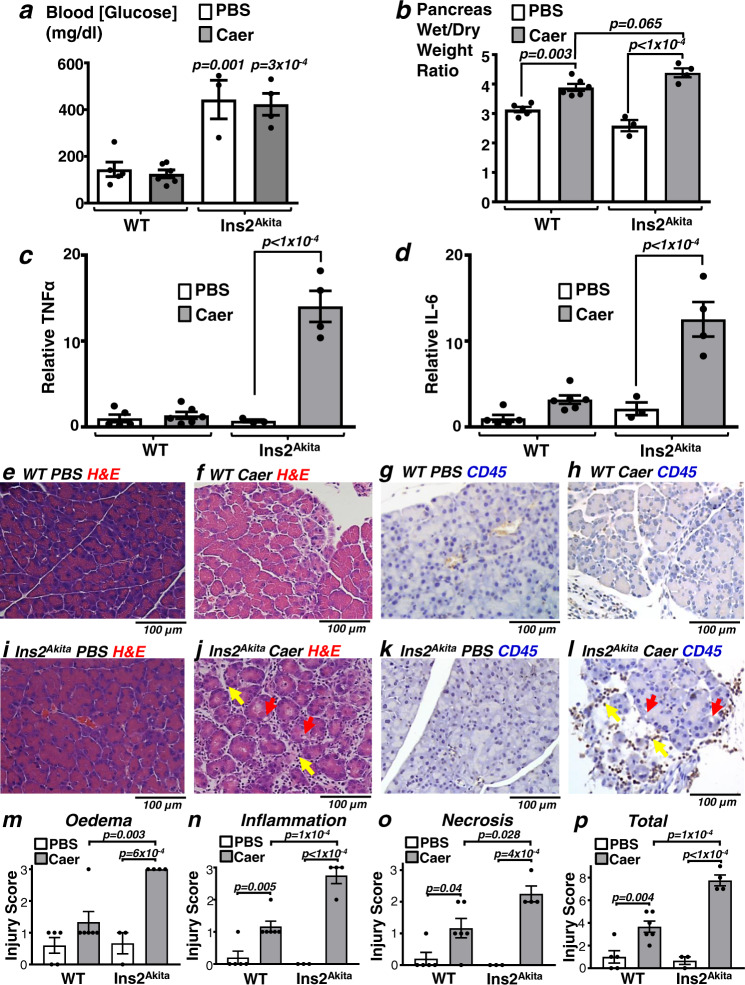
Caerulein-induced pancreatitis is more severe in type-1 diabetic Ins2^Akita^ mice vs wild type (WT) control mice. Pancreatitis was induced in WT and type-1 diabetic Ins2^Akita^ mice by caerulein (Caer; 50 µg/Kg × 8 hourly IP injections over 2 days) or phosphate-buffered saline (PBS) as a control and blood/tissue was harvested 24 h after the last injection. Blood glucose (**a**) and multiple readouts of pancreatitis were assessed (**b**–**p**). These include; pancreatic tissue oedema (wet/dry weight ratio, **b**), cytokine expression (qPCR of tissue mRNA; TNFα, **c**; IL-6, **d**), histological signs of injury (haematoxylin and eosin (H&E), **e**, **f**, **i** and **j**), immunohistochemistry of the inflammatory marker, CD45 (brown) (**g**, **h**, **k** and **l**). Yellow and red arrows in **j** and **l** indicate vacuole formation and inflammatory cell infiltration, respectively. **m**–**p** Histology injury scores (where 3 is the most severe) for oedema (**m**), inflammatory cell infiltration (**n**), necrosis (**o**), and total score (**p**; sum of other scores). Group sizes were: WT PBS, *n* = 5; WT Caer, *n* = 6; Ins2^Akita^ PBS, *n* = 3; Ins2^Akita^ Caer, *n* = 4. Significance (exact *p* values as indicated) was determined by one-way ANOVA with Sidak’s multiple comparisons. Data are presented as mean value ± SEM.

To test whether endogenous insulin had any protective effect during AP, we first induced experimental pancreatitis in parallel groups of age-matched type-1 diabetic Ins2^Akita^ mice vs control C57BL/6 wildtype mice (WT). This was done using the classical caerulein-induced hyperstimulation model of AP. Ins2^Akita^ mice were all confirmed to be hyperglycaemic, regardless of whether pancreatitis was induced (423 ± 47 mg/dl, caerulein treatment, *n* = 4; and 443 ± 82 mg/dl, PBS treatment, *n* = 3) compared to WT mice (125 ± 17 mg/dl, caerulein treatment, *n* = 6; and 145 ± 31 mg/dl, PBS treatment, *n* = 5; Fig. 1a).

For all parameters measured, pancreatitis was potentiated in Ins2^Akita^ mice compared to corresponding WT control mice (Fig. 1). Specifically, caerulein induced a 69 ± 6 % increase in oedema (pancreas wet/dry weight ratio) in Ins2^Akita^ mice compared to 24 ± 4% increase in WT control mice (Fig. 1b). Interestingly, in Ins2^Akita^ mice without pancreatitis (injected with phosphate-buffered saline (PBS)), pancreas wet/dry weight ratio was lower (2.59 ± 0.19) compared to WT mice (3.13 ± 0.09; Fig. 1b), suggesting the pancreas of Ins2^Akita^ mice is more dehydrated than WT pancreas. In addition, qPCR of pancreatic tissue mRNA revealed that pancreatic tissue cytokine expression, including TNFα (Fig. 1c) and IL-6 mRNA expression was markedly potentiated in Ins2^Akita^ mice compared to WT mice (Fig. 1d). Histologically, numerous features of pancreatitis also appeared to be aggravated in Ins2^Akita^ mice compared to WT mice. Haematoxylin and Eosin (H&E) staining of formalin-fixed paraffin-embedded (FFPE) pancreas tissue revealed that in WT mice caerulein induced intralobular oedema with ‘patchy’ areas of severe tissue injury interspersed by normal tissue (Fig. 1f compared to e). However, in Ins2^Akita^ mice, caerulein induced more severe tissue oedema that was more widespread across the tissue, rather than being “patchy” as was the case with the WT pancreas (Fig. 1j compared to i). There were clear signs of vacuole formation (red arrow, Fig. 1j) and inflammatory cell infiltration (yellow arrow, Fig. 1j), which was confirmed using CD45 immunohistochemistry of pancreas tissue counterstained with haematoxylin (Fig. 1g–l). CD45 is a non-specific cell surface marker expressed on haematopoietically-derived inflammatory cells including, neutrophils, macrophages, eosinophils^19^. Consistent with these observations, pancreatitis was further quantified histologically using a well-validated histology injury score of H&E stained slides^20–22^. These data revealed that cerulein induced a consistently higher injury score in diabetic Ins2^Akita^ mice compared to WT mice for all four criteria (Fig. 1m–p).

### Functional characterization of PACIRKO mice

**Fig. 2:**
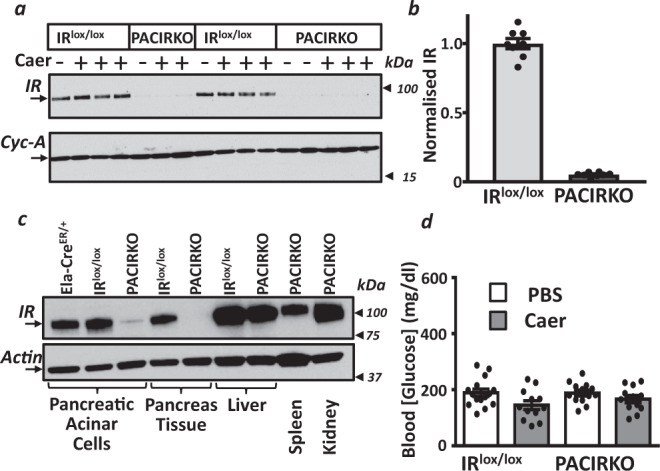
PACIRKO mice that lack pancreatic acinar cell insulin receptors are normoglycaemic. **a** Insulin receptor (IR) expression (western blot using anti-IRβ antibody) in pancreas tissue from double floxed insulin receptor (IR^lox/lox^) mice vs Pancreatic Acinar Conditional Insulin Receptor Knock Out (PACIRKO) mice following administration of tamoxifen for 4 days (oral gavage or tamoxifen feed) with (+) or without (−) caerulein (Caer; 50 µg/Kg × 8 hourly IP injections over 2 days) to induce acute pancreatitis, or PBS. Western blotting with anti-cyclophilin-A was used as a loading control. IR expression was semi-quantified by normalizing band intensities to the corresponding cyclophilin-A band and further normalisation to the mean of IR^lox/lox^ (**b**). **c** IR expression was also compared between lysates from pancreatic acinar cells and tissue from Ela-Cre^ER/+^ mice, IR^lox/lox^ and PACIRKO and also liver, spleen and kidney tissue. Actin was used as a loading control (lower panel, **c**). For each western blot of IR in **a**, **c**, the corresponding loading control (cyclophilin-A in **a** and actin in **b**) were from the same gel (membrane cut and incubated with corresponding antibody) and are representative of three separate experiments. **d** Blood glucose in IR^lox/lox^ vs PACIRKO mice, with caerulein (grey bars) or PBS white bars). Group sizes were: IR^lox/lox^ PBS, *n* = 15; IR^lox/lox^ Caer, *n* = 12; PACIRKO PBS, *n* = 13; PACIRKO Caer, *n* = 12. Data are presented as mean value ± SEM.

We next wanted to separate the confounding effects of hyperglycaemia, or reduced systemic insulin, from a loss of any direct protection of insulin on acinar cells. This was achieved using PACIRKO mice, in which the insulin receptor (IR) was specifically deleted in pancreatic acinar cells of adult mice using a tamoxifen-inducible gene deletion (Supplementary Information). Insulin receptor (IR) expression was greatly reduced following tamoxifen administration (daily oral gavage or tamoxifen feed for 4 consecutive days) in pancreatic tissue (Fig. 2a, b) and acinar cells from PACIRKO mice compared to corresponding littermate control IR^lox/lox^ mice or the feeder Ela-Cre^ER+/^ mouse line (Fig. 2c; assessed by western blotting). Moreover, IR expression is specifically reduced in the pancreas, as expression in the spleen, liver and kidney remains normal (Fig. 2c). This reduced IR expression occurred regardless of whether pancreatitis was induced with caerulein (Fig. 2a). Importantly, unlike Ins2^Akita^ mice, plasma glucose was normal in PACIRKO mice (Fig. 2d).

### Pancreatitis is worse in non-diabetic PACIRKO mice lacking acinar IRs

**Fig. 3:**
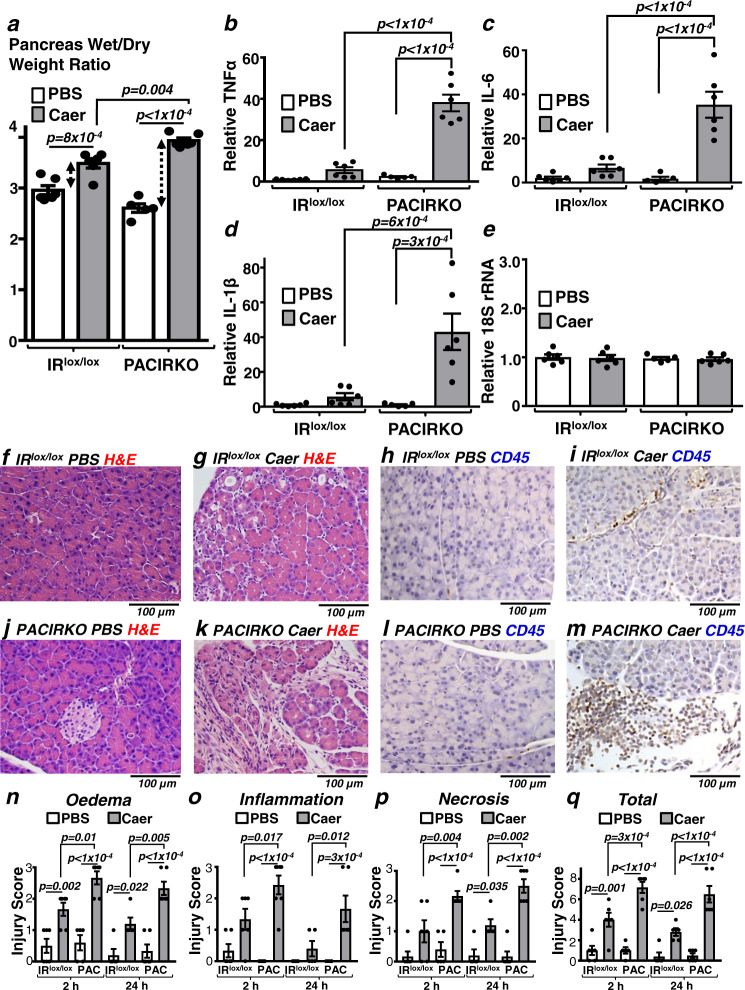
Caerulein-induced pancreatitis is more severe in PACIRKO vs IR^lox/lox^ mice. Pancreatitis was induced in control double floxed insulin receptor (IR^lox/lox^) mice vs Pancreatic Acinar Conditional Insulin Receptor Knock Out (PACIRKO) mice that lack insulin receptors (IRs) by caerulein (Caer; 50 µg/Kg × 8 hourly IP injections over 2 days) or phosphate-buffered saline (PBS) as a control and blood/tissue was harvested 2 and 24 h after the last injection. Multiple readouts of pancreatitis include; pancreatic tissue oedema (wet/dry weight ratio, **a**), cytokine expression (qPCR of tissue mRNA; TNFα, **b**; IL-6, **c**, IL-1β, **d**; and the housekeeping 18 S rRNA, **e**), histological signs of injury (haematoxylin and eosin (H&E), **f**, **g**, **j** and **k**), immunohistochemistry of the inflammatory marker, CD45 (brown) (**h**, **i**, **l** and **m**). **n**–**q** Histology injury scores (where 3 is the most severe) for oedema (**n**), inflammatory cell infiltration (**o**), necrosis (**p**), and total score (**q**; sum of other scores). Group sizes were: 2 h harvest, IR^lox/lox^ PBS, *n* = 6; IR^lox/lox^ Caer, *n* = 6; PACIRKO PBS, *n* = 5; PACIRKO Caer, *n* = 6; 24 h harvest, IR^lox/lox^ PBS, *n* = 5; IR^lox/lox^ Caer, *n* = 5; PACIRKO PBS, *n* = 6; PACIRKO Caer, *n* = 6. Significance (exact *p* values as indicated) was determined by one-way ANOVA with Sidak’s multiple comparisons. Data are presented as mean value ± SEM.

**Fig. 4:**
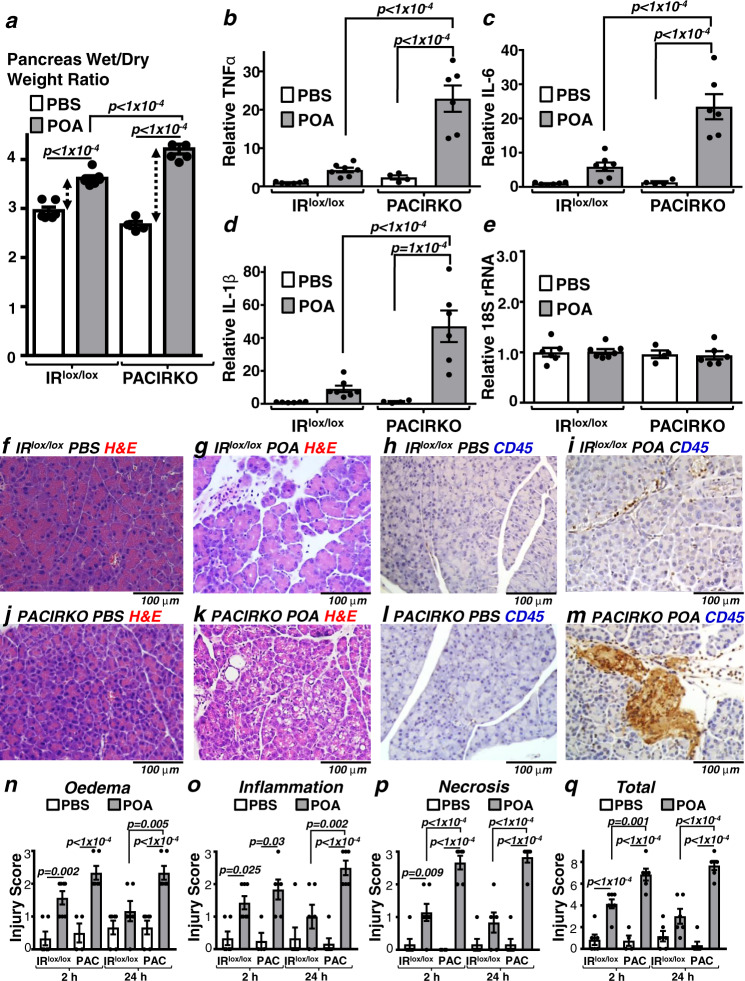
POA/ethanol-induced pancreatitis was more severe in PACIRKO vs IR^lox/lox^ mice. Pancreatitis was induced in IR^lox/lox^ and PACIRKO mice by 2 hourly IP injections of 100 µg/Kg POA in ethanol (0.8 g/Kg) or corresponding PBS control and blood/tissue was harvested 2 and 24 h after the last injection. Multiple readouts of pancreatitis include; pancreatic tissue oedema (wet/dry weight ratio, **a**), cytokine expression (qPCR of tissue mRNA; TNFα, **b**; IL-6, **c**, IL-1β, **d**; and the housekeeping 18S rRNA, **e**), histological signs of injury (haematoxylin and eosin (H&E), **f**, **g**, **j** and **k**), immunohistochemistry of the inflammatory marker, CD45 (brown) (**h**, **i**, **l** and **m**). **n**–**q** Histology injury scores (where 3 is the most severe) for oedema (**n**), inflammatory cell infiltration (**o**), necrosis (**p**), and total score (**q**; sum of other scores). Group sizes were: 2 h harvest, IR^lox/lox^ PBS, *n* = 6; IR^lox/lox^ POA, *n* = 7; PACIRKO PBS, *n* = 4; PACIRKO Caer, *n* = 6; 24 h harvest, IR^lox/lox^ PBS, *n* = 6; IR^lox/lox^ Caer, *n* = 6; PACIRKO PBS, *n* = 6; PACIRKO Caer, *n* = 6 Significance (exact *p* values as indicated) was determined by one-way ANOVA with Sidak’s multiple comparisons. Data are presented as mean value ± SEM.

Similar to Ins2^Akita^ diabetic mice, pancreatitis was worse in PACIRKO mice, either induced by caerulein (Fig. 3) or by fatty acid/ethanol^20^ (Fig. 4), compared to corresponding age-matched littermate control mice (IR^lox/lox^). Specifically, the caerulein-induced increase in pancreas wet/dry weight ratio was markedly potentiated in PACIRKO mice (51 ± 2 %) compared to IR^lox/lox^ mice (18 ± 3 %, Fig. 3a). PACIRKO pancreas wet/dry weight ratio (2.61 ± 0.08) without pancreatitis (PBS-induced) was lower compared to IR^lox/lox^ mice (2.96 ± 0.09). This suggests that PACIRKO mouse pancreas was more dehydrated than IR^lox/lox^, similar to diabetic Ins2^Akita^ mice.

In addition, pancreatic tissue cytokine expression, including TNFα (Fig. 3b), IL-6 (Fig. 2c) and IL-1β mRNA expression (Fig. 3d) was markedly potentiated in PACIRKO mice compared to IR^lox/lox^ mice. However, 18S ribosomal RNA (18S rRNA; Fig. 3e) remained unchanged suggesting that cellular RNA remains relatively intact during pancreatitis. Histologically, numerous features of pancreatitis also appeared to be worse in PACIRKO mice (Fig. 3k), compared to IR^lox/lox^ (Fig. 3g), similarly to diabetic Ins2^Akita^ mice. H&E staining revealed extensive tissue oedema and severe injury (vacuoles, necrosis, inflammatory cell infiltration and haemorrhagic areas; Fig. 3k) compared to the focal areas of less severe oedema/injury in IR^lox/lox^ mice (Fig. 3g). Inflammatory cell infiltration was further confirmed in PACIRKO mice by CD45 immunohistochemistry vs IR^lox/lox^ mice (Fig. 3h–m) and finally, histology injury scores further confirmed, quantitatively, that caerulein-induced pancreatitis was more severe in PACIRKO mice vs IR^lox/lox^ mice (Fig. 3n–q).

We next tested the effect of the more aetiologically relevant palmitoleic acid/ethanol (POA/ETOH) model^20^. This model was further optimised using lower doses of both POA and ETOH, which produced a more specific pancreatitis, and less peripheral organ injury independent of pancreatitis (Fig. S1 and Supplementary Information). Similar results were obtained using the POA/ETOH-induced model of pancreatitis to those obtained with caerulein. In PACIRKO mice, there was a potentiation of POA/ETOH-induced pancreas wet/dry weight ratio (58 ± 4% vs 22 ± 2% in IR^lox/lox^ mice; Fig. 4a), pancreatic tissue cytokine expression (Fig. 4b–e), histological features of pancreatitis (H&E; Fig. 4f–k) and CD45-positive staining (Fig. 4h–m). Likewise, there was a similar overall trend in the POA/ETOH-induced histology injury scores in PACIRKO vs IR^lox/lox^ mice (Fig. 4n–q).

### Insulin-induced protection against POA-induced cytotoxic Ca^2+^ overload is abolished in acinar cells from PACIRKO mice

**Fig. 5:**
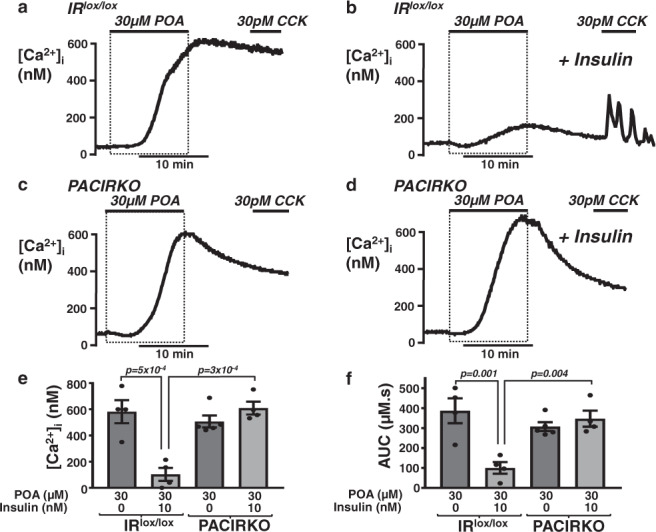
Insulin-mediated protection against palmitoleic-induced Ca^2+^ overload is abolished in pancreatic acinar cells from PACIRKO mice. Representative traces showing POA-induced [Ca^2+^]_i_ responses (**a**–**d**) in untreated fura-2-loaded pancreatic acinar cells (**a**, **c**) and following pre-treatment with 10 nM insulin for 15 min (**b**, **d**) from IR^lox/lox^ (**a**, **b**) and PACIRKO (**c**, **d**). Cells were also subsequently treated with 30 pM CCK to test for recoverability and thus cell viability post-POA treatment. Mean (±SEM) maximum increase in resting [Ca^2+^]_i_ above baseline (**e**) and mean (±SEM) area under the curve (AUC; **f**) over the treatment and recovery period in the absence (dark grey bars) or following treatment with 10 nM insulin (light grey bars). Significance (specific *p* values as indicated) was determined by one-way ANOVA with Sidak’s multiple comparisons. Data were derived from individual values from multiple cells (6-36 cells per experiment) in the field of view for each experiment. These values were averaged giving the experimental mean, that were in turn averaged across multiple experiments (four separate experiments for each experimental condition, except PACIRKO with POA, *n* = 5) giving the true mean ± SEM as indicated in (**e**, **f**).

Since pancreatitis was more severe in PACIRKO mice lacking acinar cell IRs, we next wanted to investigate the mechanism for the protective effect of insulin on acinar cells from IR^lox/lox^ mice vs PACIRKO mice. This was achieved using ‘cellular models’ of pancreatitis in which isolated acinar cells (from IR^lox/lox^ vs PACIRKO mice), were pre-treated with or without insulin (10 nM for 15 min) followed by POA to induce cytotoxic Ca^2+^ overload as a readout of cellular injury (Fig. 5). On average, 30 µM POA induced a robust increase in [Ca^2+^]_i_ by 600 ± 73 nM (Fig. 5a–e), which was dramatically reduced to 103 ± 50 nM (Fig. 5b–e), in the presence of insulin (Fig. 5f; area under the curve (AUC); 397 ± 53 µM.s with POA alone vs 100 ± 29 µM.s with insulin (*p* < 0.05)). However, this insulin-mediated protection was completely abolished in acinar cells from PACIRKO mice (Fig. 5d; POA alone increased [Ca^2+^]_i_ to 520 ± 56 nM (AUC; 315 ± 26 µM.s), while POA in the insulin-treated group increased [Ca^2+^]_i_ to 625 ± 44 nM (AUC; 361 ± 36 µM.s; Fig. 5). Collectively, these data clearly show that insulin protects mouse pancreatic acinar cells against POA-induced cytotoxic [Ca^2+^]_i_ overload, which is abolished in acinar cells from PACIRKO mice lacking acinar IRs.

### Insulin-induced protection against POA-induced PMCA inhibition is abolished in acinar cells from PACIRKO mice

**Fig. 6:**
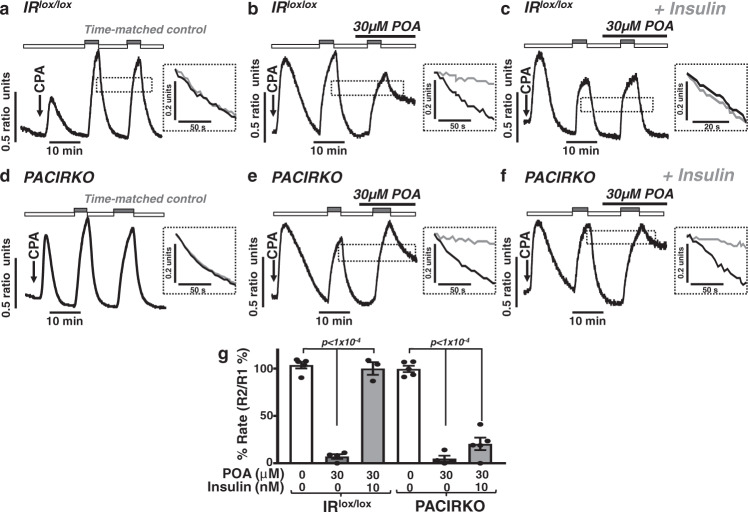
Insulin-mediated protection against palmitoleic acid-induced inhibition of the PMCA is abolished in pancreatic acinar cells from PACIRKO mice. Representative traces showing time-matched control in situ [Ca^2+^]_i_ clearance experiments (**a**, **d**) and the effect of POA (**b,** **e**) on [Ca^2+^]_i_ clearance in untreated cells (**b**, **e**) and cells pre-treated with 10 nM insulin (**c**, **f**) in pancreatic acinar cells from IR^lox/lox^ (**a**–**c**) and PACIRKO mice (**d**–**f**). Cells were treated with 30 μM CPA (arrow) in the absence of external Ca^2+^ (1 mM EGTA; white bar) or 20 mM Ca^2+^ (grey bar) to induce store-operated Ca^2+^ influx phases. POA was added prior to the re-addition of 20 mM external Ca^2+^.during the second influx-clearance phase (black bar). Inset dashed box (**a**–**f**) shows superimposed expanded time-courses of first (black trace) and second clearance phase (grey trace). Traces are representative of 5 (IR^lox/lox^ time-matched control, **a**), 4 (IR^lox/lox^ POA, **b**), 3 (IR^lox/lox^ POA with insulin, **c**), 5 (PACIRKO time-matched control, **d**). 4 (PACIRKO POA, **e**) and 5 (PACIRKO POA with insulin, **f**) separate experiments. Linear clearance rate (in the presence of POA) was normalized to the initial clearance rate in each cell (% relative clearance rate). **g** Mean % relative clearance (±SEM) of corresponding time-matched control (white bar), POA treatment (30 μM; dark grey bar), and POA with insulin (light grey bar). Significance (specifc *p* values as indicated) was determined by one-way ANOVA with Sidak’s multiple comparisons. Data were derived from individual values from multiple cells (3-19 cells per experiment) in the field of view for each experiment. These values were averaged giving the experimental mean, that were in turn averaged across multiple experiments giving the true mean ± SEM as indicated in (**g**).

To test whether the insulin protection of POA-induced Ca^2+^ overload in acinar cells was due to protection of PMCA activity we utilised a similar in situ [Ca^2+^]_i_ clearance assay to our previous studies^5,6,23^. Under these conditions, 30 µM POA caused a dramatic inhibition of relative [Ca^2+^]_i_ clearance, and thus PMCA activity, in cells from both IR^lox/lox^ mice (13 ± 6%; Fig. 6b, compared to time-matched control, 101 ± 2%, Fig. 6a) and PACIRKO mice (10 ± 3%; Fig. 6e compared to time-matched control, 98 ± 1%, Fig. 6d). However, this POA-induced inhibition of PMCA activity was completely abolished following pretreatment with 10 nM insulin in cells from IR^lox/lox^ mice (100 ± 6%; Fig. 6c) but not PACIRKO mice (21 ± 5%; Fig. 6f). These data suggest that insulin protects the PMCA from inhibition by POA which is specifically mediated through IRs, as this protection is abolished in PACIRKO mice lacking IRs.

### Insulin-induced protection against POA-induced ATP depletion is abolished in acinar cells from PACIRKO mice

**Fig. 7:**
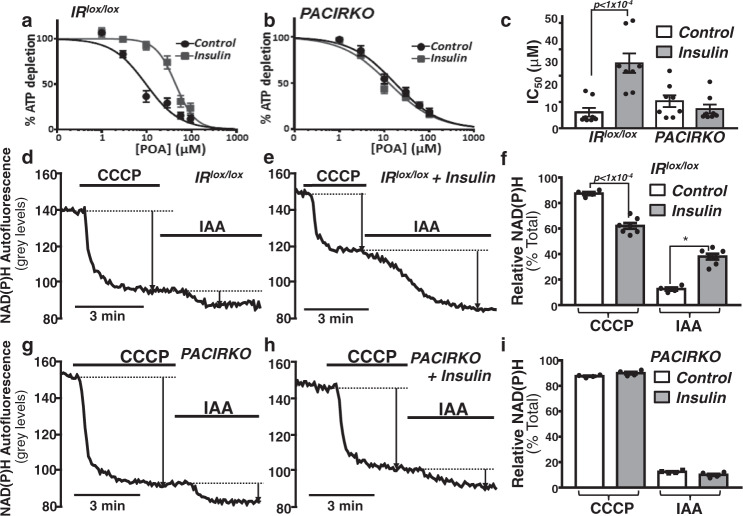
Insulin-mediated protection of POA-induced ATP depletion and switch from mitochondrial metabolism to glycolysis was abolished in PACIRKO mice. POA concentration-response curves for ATP depletion in the absence and presence of insulin (10 nM) in pancreatic acinar cells isolated from IR^lox/lox^ (**a***)* and PACIRKO mice (**b**) and corresponding mean IC_50_ values (±SEM) (**c**). Mean data are from eight separate experiments. Significance (exact *p* values as indictated) was determined by repeated measures one-way ANOVA with Sidak’s multiple comparisons. NADH autofluorescence was used to assess the relative mitochondrial vs glycolytic metabolism in pancreatic acinar cells isolated from IR^lox/lox^ (**d**, **e**) and PACRIKO mice (**g**, **h**). Sequential background-subtracted images were acquired every 5 s (500 ms exposure), and changes in NADH autofluorescence were quantified as raw fluorescence grey levels. To determine the relative mitochondrial and glycolytic contributions to NADH autofluorescence, the cells were treated with 4 μM CCCP and then 2 mM iodoacetate (IAA), respectively, and responses were normalised to the total (**f**, IR^lox/lox^; **i**, PACIRKO). Mean data (±SEM) from four separate experiments for each experimental condition, except IR^lox/lox^ with insulin (*n* = 7). Significance (specifc *p* values as indicated) was determined by either repeated measures (**c**) or ordinary one-way ANOVA (**f**, **i**) with Sidak’s multiple comparisons.

We next wanted to test whether insulin prevents POA-induced ATP depletion in IR^lox/lox^ mouse pancreatic acinar cells, using the firefly luciferase-based chemiluminescence assay^5,6,23^. In acinar cells from IR^lox/lox^ mice, POA caused a concentration-dependent ATP depletion between 1–100 µM which generated an average IC_50_ of 12.2 ± 3.3 µM and Hill slope of −1.58 ± 0.3 (Fig. 7a–c). Pre-treatment of cells with 10 nM insulin caused a rightward shift in the concentration-response curve and significantly increased the average IC_50_ to 49.4 ± 7.4 µM (*p* < 0.05; Hill slope of −2.99 ± 0.84; Fig. 7a–c). However, in acinar cells from PACIRKO mice, POA caused a similar concentration-dependent ATP depletion (IC_50_ of 20.6 ± 4.5 µM and Hill slope of −0.94 ± 0.08; Fig. 7b, c, black circles), which was unaffected by insulin pre-treatment (IC_50_ of 14.6 ± 3.4 µM and Hill slope of −0.87 ± 0.04; Fig. 7b, c). This suggests that insulin preserves ATP over the same POA concentration range (3–100 μM) that induces cytotoxic Ca^2+^ overload and inhibition of PMCA.

### Effect of insulin on cellular bioenergetics in pancreatic acinar cells

To further explore the effect of insulin on acinar cell metabolism we first measured NAD(P)H autofluorescence as an indirect measure of glycolytic vs mitochondrial metabolism^5^. The major source of cellular NAD(P)H is from the mitochondrial Krebs cycle whereas a minor source comes from the glycolytic enzyme, glyceraldehyde phosphate dehydrogenase (GAPDH). Therefore, treatment of cells with the protonophore and mitochondrial uncoupler, CCCP (4 µM), causes rapid consumption of mitochondrial NAD(P)H, and thus a reduction in NAD(P)H autofluorescence. CCCP acts by dissipating the mitochondrial proton gradient and driving force for the ATP synthase activity. The uncoupling of ATP synthesis from the electron transport chain/oxygen consumption results in mitochondrial respiration operating at ‘full tilt’ in an attempt to maintain the mitochondrial proton gradient. The resulting increased mitochondrial NADH consumption by the electron transport chain ultimately leads to mitochondrial NADH depletion. CCCP-induced NADH depletion could therefore be described as a surrogate for mitochondrial bioenergetics and thus ATP production (Fig. 7d). Subsequent inhibition of GAPDH with iodoacetate (IAA, 2 mM), caused a further smaller decrease in NAD(P)H autofluorescence (Fig. 7d). As GAPDH is a major source of glycolytic NADH, IAA-induced NADH depletion could be described as a surrogate for glycolytic bioenergetics and thus ATP production. The relative CCCP vs IAA-induced changes in NAD(P)H autofluorescence were quantified and normalised to the total decrease in NAD(P)H autofluorescence (Fig. 7f–i) and thus represents the relative mitochondrial vs glycolytic metabolism. In untreated control cells derived from IR^lox/lox^ mice, CCCP caused an 88 ± 1.1% decrease and IAA a 12.1 ± 1.1% decrease in NAD(P)H autofluorescence, respectively (Fig. 7d–f), consistent with predominantly mitochondrial metabolism and presumably ATP production. In cells pre-treated with 10 nM insulin for 15 min, the CCCP-induced decrease in NAD(P)H autofluorescence was markedly reduced to only 62.1 ± 2.3%, whereas the IAA-induced decrease in NAD(P)H autofluorescence increased to 38 ± 2.3% (Fig. 7e, f), suggesting that insulin mediates a metabolic switch towards glycolysis. However, this insulin-mediated switch towards glycolysis was abolished in acinar cells from PACIRKO mice (Fig. 7g–i).

Although these NADH autofluorescence responses are a convenient surrogate of mitochondrial vs glycolytic bioenergetics, we next wanted to investigate whether this translated to changes in cellular ATP. We used the same experimental strategy of treating cells with CCCP followed by IAA, but instead in magnesium green (MgGreen)-loaded acinar cells, which is used as an indirect measure of cellular ATP. This is because most cellular ATP exists as MgATP, therefore ATP depletion causes an increase in free Mg^2+^ concentration and thus MgGreen fluorescence (Fig. S6). Similar to NADH autofluorescence, the CCCP-induced and IAA-induced increases in MgGreen fluorescence were normalised to the total change in MgGreen fluorescence (Fig. S6c). In agreement with the changes in NADH autofluorescence, insulin treatment (10 nM for 15 min) reduced the CCCP-induced increase in MgGreen fluorescence (70 ± 3.7% to 32 ± 1.6%; Fig. S6), a measure of mitochondrial ATP depletion, but enhanced the IAA-induced increase in MgGreen fluorescence (30 ± 3.7% to 68 ± 1.5%; Fig. S6), a measure of glycolytoc ATP depetion. These data show that insulin treatment switches pancreatic acinar cell metabolism towards glycolysis, which is sufficient to preserve cellular ATP to fuel the PMCA and maintain cytosolic [Ca^2+^]_i_ homoeostasis.

To investigate this further we next tested whether insulin treatment was sufficient to preserve glycolytic flux during pancreatitis. This was assessed in isolated mouse (C57BL/6) pancreatic acinar cells in response to the pancreatitis-inducing agent POA (30 μM) with or without insulin (10 nM) treatment using the pH-Xtra Glycolysis Assay (Agilent; see Supplementary Information). This measures extracellular acidification rate (ECAR), due primarily to lactic acid efflux and thus is a convenient measure of glycolytic flux. The pH-Xtra assay utilises a dual-read ratiometric time-resolved fluorescence lifetime measurement (Fig. S7a), which is converted to extracellular pH (Fig. S7b) and [H^+^], using the MARS data analysis software (See Supplementary Methods) from which ECAR can be determined and normalised to control (Fig. S7c, mean data).

Insulin (10 nM) alone caused a marginal increase in ECAR (127 ± 14% of untreated control). However, POA (30 μM) markedly reduced ECAR to 54 ± 6% of untreated control cells (Fig. S7), which was restored to similar levels of control (96 ± 5%) by pre-incubation with insulin (10 nM). These data further support the notion that insulin maintains glycolytic flux and thus ATP production to fuel the PMCA, even in the face of POA-induced metabolic crisis.

### The insulin-mediated increase in glycolysis is due to Akt-mediated phosphorylation of phosphofructokinase fructose bisphosphatase-2 (PFKFB2)

**Fig. 8:**
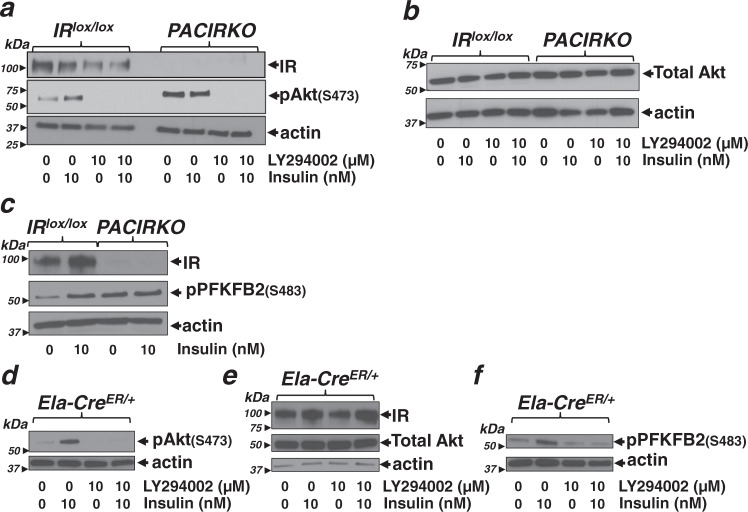
Insulin increases Akt phosphorylation and the downstream Akt-mediated phosphorylation of the key glycolytic enzyme, PFKFB2, which is abolished in acinar cells from PACIRKO mice. Pancreatic acinar cells from IR^lox/lox^ (**a**–**c**), PACIRKO (**a**–**c**) or Elast-Cre (**d**–**f**) were treated with or without 10 nM insulin and/or the PI3K inhibitor, LY294002 (10 μM) for 10 min followed by cell lysis. Proteins were separated by SDS-PAGE and western blotted using antibodies for the insulin receptor (IR, **a**, **c** and **e**), phospho-Akt (Ser473) (**a**, **d**), total Akt (**b**, **e**) and phospho-PFKFB2 (Ser483) (**c** and **f**), which recognizes the specific Akt consensus phosphorylation site. For each representative experiment shown (**a**–**f**) separate gels were run and each membrane cut and incubated with each corresponding antibody either in parallel or in series, including IR, Akt, pAkt(S473), pFKFB2 and the loading control actin. These were all sufficiently separated so that they could be resolved on the same gel. Each experiment shown (**a**–**f**) is representative of at least three independent experiments.

We next wanted to determine the specific molecular mechanism for the protective effects of insulin on acinar cells during pancreatitis. Since insulin preserved cellular ATP and switched metabolism from mitochondrial towards glycolysis, we reasoned that the regulation of glycolytic enzymes may be the primary mechanism. Specifically, we focussed on PI3K/Akt as the major downstream signalling pathway of insulin, primarily because in our previous study the PI3K inhibitor, LY294002, abolished most of insulin’s protection of POA-induced [Ca^2+^]_i_ overload in pancreatic acinar cells^6^. Therefore, we next tested whether insulin treatment led to the Akt-mediated phosphorylation of key glycolytic enzymes that may be responsible for the upregulation of glycolysis. This was initially done using co-immunoprecipitation of all Akt-phosphorylated proteins using immobilised phospho-Akt-substrate antibody followed by western blotting with antibodies for pAkt substrate antibody (positive control (Fig. S8a) and the key glycolytic enzymes, including hexokinase-I (HK-I, Fig. S8b), phosphofructokinase-1 (PFK-1, Fig. S8c), pyruvate kinase-M (PKM, Fig. S8d), pyruvate kinase-L (PK-L, Fig. S8e), and lactate dehydrogenase-A (LDHA, Fig. S8f). However, none of these glycolytic enzymes tested were found to be phosphorylated by Akt following treatment with insulin (Fig. S8). Although this is not an exhaustive list of Akt substrates, or even glycolytic enzymes and indeed there may be other enzymes responsible for the shift towards glycolysis, we decided to focus on the key glycolytic enzyme reported to be activated by insulin via Akt-mediated phosphorylation, which is 6-phosphofructo-2-kinase/fructose-2,6-biphosphatase 2 (PFKFB2)^24^. Using antibodies that specifically detect phospho-Akt (p-Akt(ser473) and the Akt-mediated phospho-PFKFB2 (p-PFKFB2(ser483)) western blotting showed that treatment of acinar cells from IR^lox/lox^ mice with insulin (10 nM) for 10 min induced phosphorylation of Akt and PFKFB2, which was completely abolished by the PI3K inhibitor LY294002 (10 μM) (Fig. 8). There was a surprisingly high basal Akt and PFKFB2 phosphorylation in untreated acinar cells from PACIRKO mice compared to acinar cells from IR^lox/lox^ mice. Importantly, however, there was no further increase in Akt or PFKFB2 phosphorylation following insulin treatment of acinar cells from PACIRKO mice, suggesting that the loss of insulin receptors was responsible for this insulin insensitivity. In acinar cells from Ela-Cre^ER/+^ mice, that express Cre (similar to PACIRKO) but also express IRs (similar to IR^lox/lox^), there was a lower basal Akt and PFKFB2 phosphorylation and insulin-induced an increase in Akt and PFKFB2 phosphorylation similar to that observed in IR^lox/lox^ mouse acinar cells (Fig. 8). This suggests the high basal Akt and PFKFB2 phosphorylation in PACIRKO mice is due to loss of IRs rather than non-specific effects of Cre expression.

The high basal Akt and PFKFB2 phosphorylation in PACIRKO mice was highly consistent and appeared specific for acinar cells from PACIRKO mice. We, therefore, investigated this further and reasoned that the upstream insulin receptor substrates (IRS-1 and IRS-2) may be responsible. Indeed western blotting revealed that both IRS-1 and IRS-2 expression was upregulated in PACIRKO mice (Fig. S9a, b). In addition, immunoprecipitation of IRS-1 or IRS-2 followed by subsequent western blotting with the pan-phosphotyrosine antibody, revealed that IRS1 (Fig. S9c), and to a lesser extent IRS-2 (Fig. S9d), were hyperphosphorylated. Furthermore, treatment of acinar cells isolated from IR^lox/lox^ mice with insulin (10 nM for as little as 1 min), induced phosphorylation of IRS-1 (Fig. S9c), but not IRS-2 (Fig. S9d), whereas acinar cells isolated from PACIRKO mice were insensitive to insulin (Fig. S9b). These data suggest that the high basal Akt and PFKFB2 were likely due to an upregulation of IRS-1 and IRS-2 expression and IRS-1 hyperphosphorylation upstream of Akt and PI3K. The mechanism for this upregulation of IRS-1/IRS-2 expression and IRS-1 hyperphosphorylation remains unclear but may reflect some kind of compensatory mechanism in response to deletion of the insulin receptor (IR). However, it is important to re-emphasise that insulin treatment of PACIRKO mouse acinar cells failed to increase phosphorylation of IRS-1 (Fig. S9c), Akt (Fig. 8a) or PFKFB2 (Fig. 8c), further supporting the notion that IR deletion prevents insulin-mediated downstream signalling and thus protection against cellular injury. On the other hand, insulin treatment of IR^lox/lox^ mouse acinar cells caused phosphorylation of IRS-1 (Fig. S9c), Akt (Fig. 8a) and PFKFB2 (Fig. 8c), as one would expect with normal IR expression.

The expression of Cre has been reported to cause non-specific toxicity regardless of deletion of specific floxed genes^25^. However, there were no signs of pancreatic injury in Ela-Cre^ER/+^ mice (Fig. S3) and CCK-evoked Ca^2+^ signalling (Fig. S4), POA-induced Ca^2+^ overload (Fig. S5) and insulin-mediated Akt phosphorylation (Fig. 8) were identical in isolated pancreatic acinar cells from Ela-Cre^ER/+^ mice vs IR^lox/lox^ mice. Therefore, the more severe pancreatitis observed in PACIRKO mice was most likely due to deletion of IRs and the loss of endogenous insulin protection of acinar cells, rather than the non-specifc potentiation of acinar cell injury induced by Cre expression. Therefore, to reduce the number of mice required for in vivo studies, experimental pancreatitis was only induced in PACIRKO and corresponding littermate control IR^lox/lox^ mice.

It is also interesting to note that the pancreatitis-induced increase in plasma amylase, a classical early diagnostic indicator of pancreatitis, was blunted in PACIRKO mice compared to corresponding IR^lox/lox^ mice (Fig. S2d, e). However, this was most likely due to the reduced pancreatic acinar cell amylase expression (Fig. S2a–c) and secretion, which is well known to be regulated by insulin and is reduced in diabetic animals^26,27^.

### Caerulein infusion-induced increase in plasma amylase is reduced by insulin administration using the hyperinsulinaemic euglycaemic clamp

**Fig. 9:**
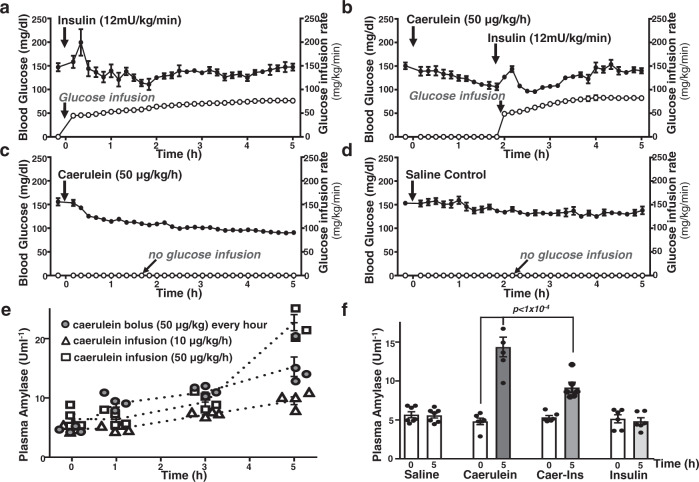
Caerulein infusion-induced increase in plasma amylase is reduced by insulin infusion with tight glycaemic control using the hyperinsulinaemic euglycaemic clamp. C57BL/6 mice were catheterized via the carotid artery and jugular vein under recover anaesthesia to allow the continuous infusion of caerulein (to induce acute pancreatic injury), insulin (12 mU/kg/min) and glucose (variable rate) to maintain euglycaemia. Caerulein-induced acute pancreatic injury was experimentally induced by continuous infusion of caerulein (50 μg/kg/h) over 5 h and plasma amylase was assessed as an early and immediate readout of pancreatic injury during the course of the experiment (**e**) and at the end (**f**). Mice were separated into 4 groups of 6 mice receiving insulin alone (**a**), caerulein and insulin (**b**, insulin added 2 h into the caerulein infusion), caerulein alone (**c**) or saline control (**d**). For clarity, data are presented as mean ± SEM assessed every 10 min during the entire 5 h. The same data are presented as dot plots showing the full data distribution with lines connecting the mean in Fig. S10. The effect of different caerulein administration regimes were compared on plasma amylase at 0, 1 h, 3 h and 5 h (mean plasma amylase ± SEM from groups of 4 mice, **e**) including; bolus caerulein injection (via IV line) every hour (open square), low dose continuous caerulein infusion (10 μg/kg/h) and high dose continuous caerulein infusion (50 μg/kg/h). This higher dose continuous caerulein infusion (50 μg/kg/h) induced the maximum increase in plasma amylase at 5 h and was therefore used in combination with the hyperinsulinaemic euglycaemic clamp (**b**). Mean plasma amylase (±SEM) from 4 groups of 6 mice measured at 0 and 5 h in saline control, caerulein alone, caerulein with insulin infusion and insulin infusion alone (**f**). Significance (exact *p* values as indicated) was determined by one-way ANOVA with Sidak’s multiple comparisons.

Given the protective effect of endogenous insulin during acute pancreatitis—which was abolished in diabetic Ins2^Akita^ mice that lack insulin secretion and PACIRKO mice that lack acinar insulin receptors—we next wanted to assess the potential protective effects of exogenously administered insulin. This was achieved by combining caerulein infusion-induced acute pancreatitis with continuous insulin infusion with tight moment-to-moment glycaemic control using the hyperinsulinaemic euglycaemic clamp. This allowed continuous high dose exogenous insulin infusion (12 mU/kg/min) and glucose infusion rate was adjusted according to blood glucose which was monitored every 10 min (via the arterial line) with a target euglycaemia of 120–130 mg/dl. This high dose insulin (12 mU/Kg/min) infusion is ~3 times higher than that normally used for insulin clamp experiments used to determine insulin resistance, and much higher than the systemic endogenous insulin concentration. However, we reasoned that this would be necessary because acinar cells in vivo are reported to be bathed in approximately 10 times higher insulin^28,29^ compared to the systemic circulation, due to the islet-acinar portal circulation^30^ (see ref. ^31^ for a general description) Initial experiments sought to determine the optimum caerulein administration by comparing continuous low dose infusion (10 µg/kg/h), high dose infusion (50 µg/kg/h) and hourly bolus caerulein (50 µg/kg) injection via the venous line (to mimic the conventional IP injections used in previous well-established models, Figs. 1 and 3). Blood samples were collected (via sampling of the IV line) at time 0, 1 h, 3 h and 5 h of the experiment for assessment of plasma amylase as an early readout of pancreatic injury. This revealed that continuous high dose caerulein infusion (50 µg/kg/h) induced the greatest increase in plasma amylase at 5 h (23 ± 1.4 Uml^−1^, *n* = 4, Fig. 9e) compared to low dose (10 µg/kg) caerulein infusion (10 ± 0.6 Uml^−1^, *n* = 4, Fig. 9e) or hourly bolus injections (15 ± 1.7 Uml^−1^, *n* = 4, Fig. 9e). Moreover, this high dose caerulein infusion (50 µg/kg/h) induced a similar increase in plasma amylase to that induced by 8 hourly IP injections of caerulein over 2 days (23 ± 2.0, *n* = 7, Fig. S1f). This suggests that this high dose caerulein infusion (50 µg/kg/h) was sufficient to induce pancreatic injury at 5 h and was therefore taken forward for the combined caerulein infusion and hyperinsulinaemic euglycaemic clamp experiments.

Blood glucose was reasonably well maintained during each experiment (insulin alone, Fig. 9a; combined caerulein/insulin, Fig. 9b; caerulein alone, Fig. 9c and saline control, Fig. 9d), with the exception of the spike in blood glucose when glucose infusion rate was increased to accommodate the onset of insulin infusion (Fig. 9a, b). This suggests that during high dose insulin infusion, tight glycaemic control can be maintained by close glucose monitoring and adjusting glucose infusion rate accordingly.

Measurement of plasma amylase at 5 h revealed that the caerulein infusion-induced an increase in plasma amylase (14 ± 1.3 Uml^−1^, *n* = 6, Fig. 9f) that was significantly reduced by high dose insulin infusion initiated 2 h after the onset of caerulein infusion (9 ± 0.6 Uml^−1^, *n* = 6, Fig. 9f, *p* < 0.05). However, insulin infusion alone had no effect (5 ± 0.5 Uml^−1^, *n* = 6, Fig. 9f) compared to saline control (5.6 ± 0.3 Uml^−1^, *n* = 7, Fig. 9f). These data suggest that exogenous insulin infusion with tight glycaemic control may reduce early pancreatic injury associated with acute pancreatitis.

Collectively, these data suggest that endogenous insulin directly protects pancreatic acinar cells during experimental models of acute pancreatitis. Loss of insulin secretion (Ins2^Akita^ mice) or loss of acinar IRs (PACIRKO mice) makes pancreatitis worse. This increase in severity of pancreatitis in Ins2^Akita^ and PACIRKO mice was due to a loss of insulin-induced Akt-mediated phosphorylation of PFKFB2, which normally preserves glycolytic ATP supply to PMCA and prevents cytotoxic Ca^2+^ overload. Finally, exogenously administered insulin using the hyperinsulinaemic euglycaemic clamp significantly attenuated caerulein infusion induced pancreatic injury associated with the early phase of pancreatitis.

## Discussion

The current study provides the first direct evidence that endogenously released insulin directly protects pancreatic acinar cell injury during two mechanistically distinct experimental models of acute pancreatitis (caerulein and POA/ethanol-induced). Impaired insulin secretion (which occurs in type-1 diabetic Ins2^Akita^ mice) and deletion of IRs in pancreatic acinar cells (which occurs in PACIRKO mice) both led to worse pancreatitis. Moreover, this study also provides the first evidence that exogenous administration of insulin, using the hyperinsulinaemic euglycaemic clamp, reduces caerulein-induced increase in plasma amylase and thus acute pancreatic injury.

Heterozygous Ins2^Akita^ male mice exhibit a single point mutation in the Ins2 gene which gives rise to inappropriate folding of pro-insulin during its synthesis, leading to ER stress and the consequent pancreatic β-cell apoptosis^32–34^. This leads to the loss of endogenous insulin secretion and the mice spontaneously develop early age onset non-obese type-1, insulin-dependent diabetes^33^. Ins2^Akita^ mice exhibit no signs of collateral injury or inflammation of neighbouring cells of the islets (insulitis) or adjacent acinar cells making this strain a good model for studying the effects of impaired insulin secretion on pancreatitis. Indeed, in the current study caerulein-induced pancreatitis was more severe in type-1 diabetic Ins2^Akita^ mice; characterised by pancreatic oedema, tissue injury (vacuoles), cytokine expression and inflammation^19^. This is consistent with clinical evidence that pre-existing diabetes increases the severity of acute pancreatitis^12^, diabetes is linked to mortality in patients with chronic pancreatitis (CP)^35,36^ and ~50% of type-1 diabetic patients exhibit pancreatic exocrine lesions characteristic of CP^11^. Moreover, the incidence of AP is higher among type-2 diabetics compared to the normal population and the risk of AP is reduced among insulin-treated diabetic patients^10^.

The current data are also consistent with a previous study in which caerulein-induced pancreatitis was aggravated in streptozotocin (STZ)-induced diabetic mice; both the acute phase of injury and regeneration of the pancreas (7 days later) was delayed^17^. Moreover, this diabetic-induced severe pancreatitis phenotype was partially corrected by exogenous administration of insulin. Although generally specific for pancreatic β cells, STZ may induce collateral exocrine injury^37^ which may further aggravate caerulein-induced pancreatitis independent of reduced insulin secretion. However, the mutation in Ins2^Akita^ mice leads to a more specific pancreatic β cell death and is more relevant to the clinical situation making this important when studying the diabetes-pancreatitis link.

Nevertheless, regardless of the specific diabetes model, it is very difficult to separate the confounding effects of hyperglycaemia [which facilitates more severe pancreatitis and sepsis^38^] or reduced systemic effects of insulin [which exhibits anti-inflammatory properties^39^] from a loss of direct insulin protection of acinar cells^5,6^. This can only be achieved using PACIRKO mice. IR expression is greatly reduced specifically in the pancreas and PACIRKO mice are normoglycaemic and insulin levels are normal^18^, thereby removing the confounding systemic effects of hyperglycaemia and loss of insulin secretion that occurs in Ins2^Akita^ mice. The PACIRKO mouse model is essential because global IR knockout mice develop early postnatal diabetes, die young due to severe ketoacidosis^40^ and pancreatic-specific IR deletion will likely affect embryonic pancreatic development^41,42^. Therefore, tamoxifen-induced deletion of IRs in adult PACIRKO mice circumvents these problems.

Results show that caerulein-induced pancreatitis is also exacerbated in PACIRKO mice, similar to Ins2^Akita^ mice. Specifically, pancreatic oedema, pancreatic injury/inflammation and pancreatic cytokine expression were all potentiated in PACIRKO mice. Caerulein is an analogue of cholecystokinin (CCK), which at high doses hyperstimulates acinar cells and induces cytotoxic Ca^2+^ overload. This mimics the downstream pathology and is a well-validated model of both acute and chronic pancreatitis^43^. However, in most cases of the human disease, caerulein does not mimic the natural aetiology. Therefore, it was necessary to compare pancreatitis in PACIRKO vs IR^lox/lox^ mice using the mechanistically distinct POA/ethanol(ETOH)-induced experimental model of pancreatitis^20^. This model was first established by Huang et al.^20^, and is more relevant than other models because not only does it mimic the downstream pathology of the disease, but also mimics the natural aetiology of alcohol-induced pancreatitis. In the present study, we used a slightly lower dose of POA (100 mg/Kg) and ethanol (0.8 g/Kg), which reduced the collateral intraperitoneal tissue injury and induced a more specific pancreatitis compared to ethanol alone (Fig. S1).

Similar to caerulein-induced pancreatitis, we also observed qualitatively identical potentiation of pancreatitis induced by POA/ETOH in PACIRKO mice. Collectively, these data suggest that endogenous insulin directly protects pancreatic acinar cells during pancreatitis and deletion of acinar IRs, as is the case with PACIRKO mice, makes pancreatitis worse. The advantage of using PACIRKO mice is that it removes the confounding effects of hyperglycaemia or loss of systemic insulin secretion which occurs in Ins2^Akita^ mice. A similar loss of direct protection of insulin on pancreatic acinar cells, due to insulin resistance, might also explain why type-2 diabetics have an ~3 fold increased risk of developing acute pancreatitis^9,13,14,44^.

This study also provides the first evidence that exogenous therapeutic administration of insulin with tight moment-to-moment glucose control, using the hyperinsulinaemic euglycaemic clamp reduces early pancreatic injury (plasma amylase) associated with acute pancreatitis (AP). Although further pre-clinical animal studies are required to fully characterise these responses and optimise the insulin dose regimen, these data suggest that insulin has *bona fide* therapeutic potential that could be translated to patients with severe AP (SAP). This is because the standard of care for SAP patients on critical care is fluid resuscitation and supportive nutritional support frequently administered via a parenteral IV line. This makes SAP patients on critical care highly amenable to the hyperinsulinaemic euglycaemic clamp as a therapeutic strategy.

Insulin therapy is routinely used to specifically treat hypertriglyceridaemia (HTG)-induced pancreatitis^45,46^, characterised by plasma lipids exceeding 1,000 mg/dL (normal range, 101–150 mg/dL). Although HTG-induced pancreatitis is relatively rare (~2–10% of all AP cases), HGT is a well-documented aetiological risk factor for severe disease^47^. The rationale for insulin treatment for HTG-induced SAP is to lower plasma triglycerides, thereby limiting inflammation^48^. This is because insulin activates lipoprotein lipase (converts triglycerides into free fatty acids) and inhibits hormone-sensitive lipase (liberates triglyceride from adipocyte). However, given the evidence presented in the present study, it’s entirely possible that the beneficial effects of insulin therapy in HTG-induced pancreatitis patients may also be due to a direct protection of acinar cell injury.

Its also worth noting that intensive insulin therapy is the standard of care for all critically ill patients, regardless of underlying disease, with the aim of reducing hyperglycaemia associated with the acute phase of injury which facilitates inflammation and sepsis^49^. Although several clinical studies and meta-analyses question the overall patient benefit^50–54^, careful scrutinization of these studies reveal that fewer patients receiving insulin die from sepsis, but rather die from the complications of inadvertent hypoglycaemia as a result of inadequate glucose control. Moreover, it is unclear from any of these studies whether any SAP patients specifically benefited from intensive insulin therapy as patients were only identified within broad disease categories (e.g. GI diseases). Furthermore, the present study used a relatively high dose insulin infusion (12 mU/kg/min—approx three times higher than the normal systemic concentration) with close glucose monitoring (every 10 min) to maintain tight blood glucose. The rationale for such high dose insulin is because in vivo pancreatic acinar cells receive a portal blood flow direct from the islets^30^ containing approximately ten times higher insulin concentration than the systemic circulation^28,29^. Furthermore, stress hormones, such as adrenaline, cortisol and glucagon, and inflammatory cytokines (TNFα and IL-1β) reduce the tissue sensitivity of insulin, so a higher dose of insulin may be necessary to overcome this during the acute phase of injury.

The safety record of the hyperinsulinaemic euglycaemic clamp is well recognised as it is used routinely to test for insulin resistance. Moreover, high dose insulin infusion (8 mU/kg/min; adjusted for surface area to weight ratio) with tight physiological glucose control has been tested in healthy human volunteers during endurance exercise studies with no reported adverse effects^55^. Nevertheless, the use of high dose insulin, even with tight glycaemic control, would have to be approached with caution in SAP patients in critical care, given the potential problems of ketoacidosis, hypokalemia or other metabolic disturbances.

**Fig. 10:**
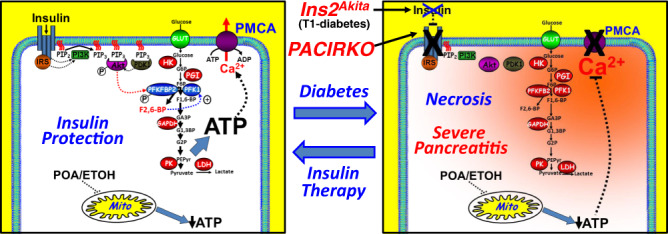
Cartoon depicting the putative molecular mechanism for insulin protection of pancreatic acinar cells during acute pancreatitis. Pancreatitis inducing agents, such as fatty acid/ethanol metabolite palmitoleic acid (POA) impair metabolism, most notably mitochondrial function, leading to ATP depletion and inhibition of Ca^2+^ clearance pathways, such as the plasma membrane Ca^2+^ ATPase (PMCA), leading to cytotoxic Ca^2+^ overload. The net effect of ATP depletion and cytotoxic Ca^2+^ overload is rapid necrotic cell death, a major hallmark of acute pancreatitis. Insulin binding to the insulin receptor leads to the downstream activation of the PI3K/Akt signalling pathway and the direct Akt-mediated phosphorylation and activation of the key glycolytic enzyme, phosphofructokinase fructose bisphosphatase-2 (PFKFB2). This leads to the production of the key glycolytic metabolite fructose 2,6-bisphosphate, which is an allosteric activator of the rate limiting phosphofructokinase-1 (PFK1), which in turn drives glycolytic flux and thus glycolytic ATP production and supply to the PMCA. This appears to be sufficient to maintain acinar cell ATP and prevent inhibition of the PMCA, even in the face of impaired mitochondrial function, thereby protecting against necrotic cell death and the self-perpetuating tissue injury and spiralling inflammatory response characteristic of acute pancreatitis. During diabetes (Ins2^Akita^ mice) and in PACIRKO mice, this endogenous insulin protection is abolished, leading to more severe acute pancreatitis. Moreover, exogenous insulin administration, using the hyperinsulinaemic euglycaemic clamp to infuse high dose insulin while maintaining close glucose control, ameliorates or protects against further pancreatic injury suggesting a potential therapy for severe acute pancreatitis.

The present study also showed, using cellular models of pancreatitis, that insulin signalling protects against POA-induced ATP depletion, inhibition of the PMCA and the consequent cytotoxic Ca^2+^ overload. This was due to an insulin-mediated metabolic shift towards glycolysis, most likely due to Akt-mediated phosphorylation of the rate-limiting glycolytic enzyme PFKFB2. This preserves cellular ATP and fuels the PMCA, thereby maintaining low resting [Ca^2+^]_i_, even in the face of impaired mitochondria by pancreatitis-inducing agents (see Fig. 10). A conceptually similar ‘boost’ in metabolism and preservation of ATP has been recently suggested to be responsible for the protective effect of galactose supplementation during pancreatitis^56^. Glycolytic enzymes are also reported to be associated with the plasma membrane of erythrocytes, smooth muscle cells and pancreatic cancer cells where they provide a privileged ATP supply to the PMCA^57–64^. Cutting off this ATP supply in pancreatic cancer cells reduced numerous hallmarks of cancer and induced cytotoxic Ca^2+^ overload and cell death^63,64^. If extrapolated to acinar cells, such insulin-mediated upregulation of this localized glycolytic ATP supply to the PMCA may be all the more critical when mitochondria, the major source of ATP, are impaired and global ATP is severely depleted.

PFKFB2 is a member of the PFKFB family of bi-functional glycolytic enzymes that convert F6P to F2,6BP, via their kinase activity, and also catalyzes the reverse reaction by converting F2,6BP back to F6P, via their bisphosphatase activity^24^. F2,6BP is a potent positive allosteric activator of the rate-limiting irreversible glycolytic enzyme, PFK-1, which converts F6P to F1,6BP, thereby maintaining glycolytic flux^65^. The relative kinase to bisphosphatase activity, and thus the production of F2,6BP, varies between the different PFKFB isoforms; with PFKFB1/4 expressed in the liver exhibiting almost no kinase activity, which therefore acts as a break in glycolysis, promoting glycogen synthesis and flux through the pentose phosphate pathway important for nucleotide and amino acid biosynthesis^65^. On the other hand, PFKFB3 (which is expressed in cancer cells) has a 700 fold higher kinase activity, which means that glycolytic flux is essentially maximally activated and thus contributes to the Warburg effect in cancer cells^66^. PFKFB2 is a tunable glycolytic enzyme with approximately equal kinase vs bisphosphatase activities, but its kinase activity is dramatically increased following phosphorylation by Akt, PKA and AMPK^65^. Therefore, phosphorylation of PFKFB2 acts as a ‘volume control’ for glycolytic flux and thus ATP production and is thus likely to be the major molecular mechanism for the protective effects of insulin during pancreatitis (see Fig. 10).

It is also interesting to note that the expression of amylase was also reduced in pancreatic acinar cells of PACIRKO mice. Moreover, the pancreatitis-induced increase in plasma amylase, a classical early clinical biomarker for pancreatitis, was blunted in PACIRKO mice. This is particularly interesting because there have been numerous older studies that show that the expression and synthesis of amylase is matched by the relative carbohydrate composition in the diet^67–69^, which in turn is controlled by insulin (and insulin receptors within acinar cells)^26,27,70^. Specifically, fasting reduces acinar amylase, which can be restored by glucose administration, but this is prevented by diazoxide which inhibits glucose-dependent insulin secretion^71–73^. Conversely, and consistent with the present study, acinar amylase content (expression and synthesis) is severely reduced in type-1 diabetic rat models (streptozotocin and alloxan), which is restored by insulin administration^26,27,70^. Similarly, acinar amylase content is also reduced in hyperinsulinaemic insulin-resistant diabetes models (Zucker rat) and restored by ciglitazone (reduces insulin resistance and normalizes glucose)^74^. Therefore, during pancreatitis when acinar cells undergo necrosis and thus cell lysis there is potentially less amylase available to be released into the interstitium and thus plasma, which helps to reconcile these results.

It was also observed in Ins2^Akita^ and PACIRKO mice that the wet/dry weight ratio was significantly lower in mice without pancreatitis compared to the corresponding control mice (WT or IR^lox/lox^, respectively). This suggests that the pancreas tissue is more dehydrated in Ins2^Akita^ and PACIRKO mice than control mice, suggesting that endogenous insulin might regulate tissue water content. However, this is independent of systemic hyperglycaemia, and thus osmotic diuresis, as this occurs in PACIRKO mice that are normoglycaemic. Therefore, this suggests that insulin regulates acinar cell ion/volume regulation and thus tissue fluid homoeostasis specifically mediated through activation of acinar cell IRs. It is interesting to note that insulin, and its downstream signalling pathways, have been shown to regulate numerous ion transport pathways, such as Na^+^/K^+^/2Cl^−^ cotransporter (NKCC)^75–81^ and Na^+^/K^+^-ATPase^77,79,82,83^ that are critical for regulation of cell volume and thus fluid homoeostasis. It is also worth mentioning that the loss of NKCC activity, for example during inhibition with the loop diuretic bumetanide, reduces insulin sensitivity in hepatocytes^84^, suggesting that cell volume regulation is important for maintaining insulin effectiveness. Therefore, if extrapolated to pancreatic acinar cells, this suggests that the loss of NKCC activity and cell volume regulation during diabetes may further exacerbate acinar cell injury and thus contribute to the severity of pancreatitis.

In summary, the current study provides a mechanistic link between diabetes and the severity of acute pancreatitis (AP). Moreover, this study provides evidence that exogenous therapeutic administration of insulin with tight moment-to-moment glucose control, using the hyperinsulinaemic euglycaemic clamp reduces early pancreatic injury associated with AP. Therefore, insulin infusion with the aim of reducing pancreatic injury may prove effective for the treatment of SAP, as long as there is tight moment-to-moment glycaemic control (hyperinsulinaemic euglycaemic clamp). Finally, the present study has identified phosphorylation of PFKFB2 as a potential therapeutic strategy for the design of new drugs, or the repurposing of existing drugs, for the treatment of AP. In addition to Akt-mediated phosphorylation, PFKFB2 can be phosphorylated at the exact same serine residue (S483) by AMP-dependent kinase (AMPK) and/or protein kinase-A, suggesting that AMPK activators or drugs that elevate cAMP might mimic the protective effects of insulin. Interestingly, the anti-diabetic drug metformin is also an AMPK activator, suggesting that this could be repurposed to treat acute pancreatitis. Such drugs might have a more specific protective effects without some of the more adverse systemic effects, technical difficulties or safety issues of insulin administration that could be used to treat all forms of acute pancreatitis from mild to severe disease.

## Methods

Further information and requests for resources, reagents and access to data should be directed to Jason Bruce (jason.bruce@manchester.ac.uk). A more comprehensive description of the methods and list of reagents can be found in Supplementary Information. All studies with research animals, including in vivo experiments and experiments using cells/tissues derived from research animals complied with all relevant ethical regulations regarding the use of research animals (see below).

### Animal study approval

All animal procedures and in vivo experiments were approved by the University of Michigan Institutional Animal Care and Use Committee (IACUC). The breeding of PACIRKO mice and feeder strains (Ela-Cre^ER/+^ and IR^lox/lox^) at the University of Manchester, which includes the administration of tamoxifen to induce insulin receptor deletion was approved by the Home Office Project licence (PPL number, P08B76E2B; PPL holder, Michael Simonson-Jackson).

### Ins2^Akita^ and PACIRKO mice

All experiments using Ins2^Akita^ mice were performed on male heterozygous mice at 6–9 weeks, as this was empirically determined to be the time of onset of consistent hyperglycaemia. Ins2^Akita^ mice were a kind gift from Peter Arvan (Department of Internal Medicine, Unversity of Michigan). PACIRKO mice were originally generated from the feeder strains Elastase-Cre-ER mice (Elast-Cre^ER/−^) double floxed insulin receptor mice (IR^lox/lox^) as previously described^18^. Both the experimental mice (PACIRKO) and age-matched littermate control mice (IR^lox/lox^) were administered tamoxifen (75 mg/kg) by oral gavage daily for 4 days at 5–6 weeks and on day 7 pancreatitis was induced using either caerulein or POA/ETOH. IR expression was confirmed and western blotting with an anti-IRβ antibody (Cell Signaling). For additional details about breeding and genotyping of Ins2^Akita^ and PACIRKO mice see Supplementary Information. All mice are kept in individually ventilated cages with 12 h light: 12 h dark cycles maintained at 22 °C ± 2 °C and between 45 and 65 relative humidity with free access to food and water. These conditions are in accordance with the Codes of Practice for the care and accommodation of animals under section 21 of the Animals (Scientific Procedures) Act 1986 as amended in 2012 (‘ASPA’).

### Caerulein-induced experimental pancreatitis

Mice received eight hourly intraperitoneal (IP) injections of 50 μg/Kg caerulein per day over two consecutive days and were euthanized by CO_2_ asphyxiation followed by cervical dislocation 2 h or 24 h after the last caerulein injection. Blood was immediately collected for assessment of blood glucose and plasma amylase (Phadebas amylase test, Magle Life Sciences). Whole pancreas tissue was rapidly dissected, weighed and cut into sections for processing for histology (10% formalin), RNA (RNA later, ThermoFisher), protein (snap frozen in liquid nitrogen, prior to homogenisation in lysis buffer) and wet/dry weight ratio (weighed before and after drying in an oven at 90 °C for 24 h).

### POA/ETOH-induced experimental pancreatitis

Mice received one IP injection containing PBS followed by two hourly IP injections of 100 µg/Kg POA, 0.8 g/Kg ethanol (ETOH). This was a slightly lower dose of POA and ETOH used in the study that originally characterised this model^20^ and was fully optimized in the current study to produce the least collateral organ injury and a more specific pancreatitis (Supplementary Information). Similar to the caerulein model, mice were euthanized 2 h or 24 h after the last injection and tissue/blood were harvested and processed in the same way.

### RNA extraction and quantitative real time PCR

Pancreatic tissue stored at 4 °C in RNA later was transferred to TRIzol reagent (Ambion Life Technology) and homogenised using a polytron for 5–10 s. RNA was isolated using chloroform/isopropanol extraction and RNeasy spin column kit (Qiagen). Following quantification and assessment of purity using the Nanodrop 280/260 nm optical density ratio (OD_280_/OD_260_ ratios), isolated (200 ng) was reverse transcribed into cDNA using *Taq*Man reverse transcription reagents (Thermofisher) with random hexamers as primers. Quantitative PCR reactions were carried out using the Absolute Blue SYBR Green ROX reagent (Thermo Scientific, Waltham, MA) with specific primers (listed in Supplementary Table 1 and ref. ^85^).

### SDS PAGE and western blotting

Frozen tissue that had been snap-frozen in liquid nitrogen following dissection was homogenized in lysis buffer (in mM: 50, Tris-HCl; 50, NaCl; 5, EDTA, 0.2% triton X-100; 10 mM NF; 10, Na_4_P_2_O_7_; 25, glycerophosphate, 1, DTT; 1, PMSF; 0.2, Na_3_VO_4_; 10 µg/ml leupeptin; 10 µg/ml aprotinin). For phosphorylation assays (phospho-Akt and phosphor-PFKFB2), isolated pancreatic acinar cells were resuspended in a similar lysis buffer containing protease inhibitor cocktail tablets (Roche) and PhosSTOP (Roche). Lysates were allowed to solubilize at 4 °C for 30 min followed by sonication and centrifugation at 16,000 × g for 10 min to remove insoluble debris (pellet). Sample protein was determined (Bradford assay, Bio-Rad Laboratories), denatured by boiling in SDS-Laemmli buffer for 5 min, separated using sodium dodecyl sulphate electrophoresis-polyacrylamide gel electrophoresis (SDS-PAGE), transferred to nitrocellulose and western blotted using specific antibodies at 1:1000 dilution (unless otherwise stated) to insulin receptor (IRβ mAB), amylase (rabbit anti-α-amylase, used at 1:15,000 dilution), Akt1 (C73H10) (rabbit mAb used at 1:14,000) phospho-Akt substrate, phospho-Akt (Ser473) (used at 1:15,000 dilution), phospho-PFKFB2 (Ser483) (used at 1:5,000 dilution), IRS-1 (rabbit MAb;), IRS-2 (mouse MAb), pan-phosphotyrosine (clone 4G10; used at 1:8,000 dilution), HKI, PFK-1, PKM, PKL/R, LDH and actin (used at 1:100,000) or cyclophilin-A were used as a loading control (Supplementary Table 1, Supplementary Information). For immunoprecipitation, cells were lysed in Radioimmune Precipitation Assay (RIPA) buffer (containing 50 mM Trizma® base, 1 mM EDTA, 1 mM EGTA, 0.1 mM vanadate, 1 mM NaF, 40 mM Na_4_P_2_O_7_, 1% Triton X-100, 0.1% SDS, and supplemented with cOmplete EDTAfree protease inhibitors and PhosSTOP phosphatase inhibitors), sonicated and left to solubilize for 30 min on ice^86^. Protein lysates containing around 1 mg/ml of protein were incubated with either IRS-1 or IRS-2 antibody (1 ml lysate; 1:100 dilution) overnight at 4 °C on a rocking platform. Protein lysates were incubated for a further 2 h at 4 °C with magnetic protein-G Dynabeads. Immunoprecipitates were washed five times with lysis buffer, denatured in SDS Laemmli sample buffer and boiled for 15 min at 100 °C. Immunoprecipitated samples were then separated by SDS and western blotted.

### Plasma amylase

Frozen plasma harvested from experimental mice was slowly defrosted and assayed for amylase concentration using Phadebas reagent (Supplementary Information).

### Histological assessment of pancreatitis

Pancreatic tissue was formalin-fixed (10% formalin at 4 °C for 24 h, followed by 70% ethanol) and paraffin-embedded (FFPE), prior to cutting into 5 μm sections and mounting onto slides (Supplementary Information). Tissue sections were stained with haemotoxylin and eosin (H&E) using a standard protocol by the University of Michigan Cancer Center Histology Core Facility. All pancreatic tissue sections were imaged, archived and analysed using the 3D-Histech Pannoramic-250 microscope slide-scanner (University of Manchester Bioimaging Facility) (Supplementary Information). Slides from all animal groups were graded by two independent, blinded observers according to severity and extent of oedema, inflammatory cell infiltration and acinar necrosis using a well-validated histology injury score on a scale of 0–3 (where 3 was the most severe). The total score was the sum of the oedema, inflammation and necrosis scores for each slide (maximum score of 9)^87^. To further assess pancreatic tissue inflammation, immunohistochemistry using anti-CD45 antibody (1:50 dilution) was performed on FFPE pancreatic tissue sections (Supplementary Information).

### Pancreatic acinar cell isolation

Pancreatic acinar cells from IR^lox/lox^, PACIRKO and Ela-Cre^ER/+^ mice were isolated by collagenase-digestion which was adapted from previous methods^23,88^ and described in detail in Supplementary Information. Mice were humanely killed by an approved method as set out in Schedule 1 of the UK Animals Scientific Procedures Act 1986 (Certificate of Designation No 50/2506). Briefly, following rapid dissection the pancreas was chopped and incubated in HEPES-buffered physiological saline (HEPES-PSS; for composition see Supplementary Information) containing 0.15 mg ml collagenase P (~200 U/ml) and 0.15 mg/ml of soybean trypsin inhibitor (Sigma) for 25 min at 37 °C with mechanical trituration every 5 min to break up the tissue into small cell clusters. Cell clusters were resuspended in Dulbecco’s Modified Eagle Medium (DMEM) containing 2.5% FBS, and 0.15 mg/ml of trypsin inhibitor and left to rest for 30 min at 37 °C prior to any experimentation.

### Imaging of fura-2 fluorescence

Pancreatic acinar cells were loaded with 4 μM fura-2-AM for 30 min at room temperature in HEPES-PSS and imaged using a Nikon TE2000S microscope with ×40 oil immersion SFluor objective lens, CoolSNAP HQ CCD camera (Photometrics, Tucson, AZ), Cairn monochromator (Cairn Research, Kent, UK) and MetaFluor imaging software (Molecular Devices, Downington, PA)^23^. Background-subtracted fluorescence images were captured with 50 ms exposure and 5 × 5 binning every 5 s. The fura-2 fluorescence was calibrated into [Ca^2+^]_i_ using the well-established Grynkiewicz method^88^. All imaging experiments were carried out at room temperature (20–22 °C).

### In situ Ca^2+^ clearance assay

Cells were treated with the sarco/endoplasmic reticulum Ca^2+^-ATPase (SERCA) inhibitor, cyclopiazonic acid (CPA) in zero external Ca^2+^ (0 Ca^2+^, 1 mM EGTA) to depelete ER Ca^2+^ and activate store-operated Ca^2+^ entry (SOCE). Addition of 20 mM external Ca^2+^, therefore, results in a rapid increase in [Ca^2+^]_i_ which reaches a steady state and the subsequent removal of external Ca^2+^ (0 Ca^2+^, 1 mM EGTA) causes a rapid [Ca^2+^]_i_ clearance due predominantly to PMCA activity. Repeated Ca^2+^ influx-efflux phases allow POA to be applied during the second phase and compared to the initial clearance phase. [Ca^2+^]_i_ clearance rate (and thus PMCA activity) in the presence or absence (time-matched control) of POA during this second clearance phase is quantified by measuring the linear rate from a standardised value of [Ca^2+^]_i_ and normalised to the corresponding linear rate during the first clearance phase^23^.

### Measurement of cellular ATP

ATP depletion was assessed using fire-fly luciferase (ViaLight® Plus kit; Lonza, Rockland, ME USA) as previously described^5^. Cells were treated with or without 10 nM insulin for 20 min, followed by treatment with or without various concentrations of POA (1–100 µM). In the same 96 well plate some wells were treated with an ATP depletion cocktail, consiting of 4 μM CCCP, 500 μM bromopyruvate, 10 μM oligimycin and 2 mM iodoacetate, for a further 20 min to induce maximum ATP depletion. The total luminescence count of the ATP-depletion cocktail was subtracted from each corresponding assay condition prior to normalisation to the corresponding time-matched controls and expressed as a % and data presented as sigmoidal concentration-response curves.

### Imaging of NADH Autofluorescence

Pancreatic acinar cells were excited with light at 350 nm (500 ms exposure) and NADH autofluorescence collected through a fura-2 400 nm dichroic filter without a band pass filter. Sequential background-subtracted images were acquired every 5 sec and changes in NADH autofluorescence quantified as raw fluorescence grey levels. To determine the relative mitochondrial and glycolytic contributions to NADH autofluorescence, cells were treated with 4 µM CCCP and then 2 mM iodoacetate (IAA), respectively.

### Imaging magnesium green (MgGreen) fluorescence

Pancreatic acinar cells isolated from IR^lox/lox^ mice were loaded with 4 µM MgGreen acetoxymethyl ester (AM) for 30 min at room temperature (see Supplementary Information and refs. ^5,6^).

### Measurement of glycolysis using the pH-Xtra Glycolysis Assay

Acinar cell glycolysis was assessed by real-time, kinetic analysis of extracellular acidification rate (ECAR) using a pH-Xtra Glycolysis Assay kit (Agilent) and a plate reader with time-resolved fluorescence (TRF) capability (CLARIOstar, BMG Labtech). Extracellular acidification is mainly due to lactic acid efflux, which results in a reduction in assay buffer pH. The pH Xtra probe sensitively detects this reduction in pH as an increase in sensor signal. Briefly, isolated mouse pancreatic acinar cells were washed three times with respiration buffer and pre-incubated in the presence or absence of 10 nM insulin for 30 min followed by the addition of 30 μM POA. After 15 min, 40 μl of cell suspension per condition was transferred to a 96 half area well plate and 10 μl of pH-Xtra reagent (fluorescent probe) was added to each well. Blank control wells were included and cell-free negative and positive (glucose oxidase) wells were used as signal controls. Each condition was carried out in triplicate. Both the metabolic incubations and the measurements were carried out at 37 °C.

Dual read TRF lifetime detection mode was performed and the change in fluorescent lifetime from 100 to 300 µs delay and a 30-µs read window (excitation 340 ± 50 nm, emission 615 ± 18 nm) was monitored over time. The two TRF intensity readings collected were used to calculate the ratio-metric fluorescence lifetimes using a data analysis software (MARS, BMG Biotech), which applies the following formula: Lifetime (μs)[τ] = (D2−D1)/ln(IW1/IW2) where IW1 and IW2 represent the two (dual) measurement windows and D1 and D2 represent the delay time prior to measurement of W1 and W2, respectively. Lifetime values (μs) were corrected based on the blank wells and represent the extracellular acidification in each individual sample, then scaled to [H+] using the same software tool.

### Combined caerulein infusion-induced pancreatitis and hyperinsulinaemic euglycaemic clamp

Mice (C57BL/6) were anesthetized with an intraperitoneal injection of sodium pentobarbital (50–60 mg/kg). The ventral side of the neck and back of the head were shaved and the skin repeatedly scrubbed with iodine and 70% ethanol. Under aseptic conditions, a small incision at the right ventral side of neck was made cephalic to the sternum at 30° to the midline, exposing the right carotid artery and jugular vein. The right jugular vein was catheterized using silicon tubing (0.025′′OD). The right carotid artery was catheterized using a two-part catheter constructed from polyurethane (0.010′′OD, with interior coated with heparin) and silastic tubing (0.025′′OD). The arterial catheter was inserted reaching the level of the aortic arch and the venous catheter was extended to the level of the right atrium. To allow free movement of the animal post-recovery, the free ends of catheters were tunnelled subcutaneously and exteriorized at the back of the neck via a stainless steel tubing connector (coated with medical silicon) that were fixed subcutaneously upon the closure of the incision. The catheters were flushed daily after surgery with saline and sealed with heparin-saline (200 U/ml). Following surgery, mice were allowed to recover and acclimatize for 5 days prior to experimentation and were housed individually and their body weight monitored daily. Animals that had the healthy appearance, normal activity, and body weight regained to or above 90% of their pre-surgery level were used for the study.

Mice were fasted for 5–6 h prior to beginning the caerulein infusion/insulin clamp. The exteriorized catheters were connected to a series of automated syringe minipumps (Pump 11 Elite, Harvard Apparatus) to allow the separate adjustment of infusion rate for saline, caerulein, insulin and glucose. A blood sample (~100 µl) was taken 10 min prior to the start of the caerulein infusion/insulin clamp (*t* = −10 min), for assessment of blood glucose (Accu-Chek glucose meter, Roche). The caerulein infusion (and corresponding saline control) and insulin clamp (insulin control) were begun at time zero (*t* = 0). For the combined caerulein infusion/insulin clamp, the insulin clamp was begun 2 h into the caerulein infusion (*t* = 2 h). Initial experiments sought to determine the optimum caerulein administration by comparing low dose infusion (10 μg/kg/h), high dose infusion (50 μg/kg/h) and hourly bolus injection (50 μg/kg), via the venous line (to mimic IP injections of caerulein used in previous well-established models, Figs. 1 and 3). The insulin clamp was begun with a prime-continuous infusion (16 mU/kg bolus, followed by 12.0 mU/kg/min or 72 pmol/kg/min) of human insulin (Novo Nordisk) and continued for either 5 h (insulin control) or the remaining 3 h (combined caerulein/insulin). Tight glycaemic control (target euglycemia of 120–130 mg/dL) was maintained during the insulin clamp by measuring blood glucose every 10 min starting at *t* = 0 and infusing 50% glucose at variable rates accordingly. Blood samples were collected every 10 min via the arterial line for assessment of plasma amylase as an early readout of pancreatic injury at the end of the 5 h infusion experiment. A continuous infusion of erythrocytes obtained from donor mice via cardiac puncture was given at 4 µl/min throughout the experiment period to prevent anaemia from the repeated blood sampling.

### Quantification and statistical analysis

For all experiments data are presented as representative trace, gels and histological image of at least 3–8 separate experiments and average data are presented as a combined scatter plot (to show individual data points) and bar chart with mean (±SEM). All data were analysed, transformed and figures generated using Graphpad prizm v7 and Microsoft Excel 2010. Statistical significance was determined using either a two-way Students *T*-test, one-way ANOVA with Sidak’s multiple comparison, repeated measures one-way ANOVA with Sidak’s multiple comparison or Kruskal–Wallis test with Dunn’s multiple comparison (if non-parametric) to determine significance. The specific tesdt used was included in legend text for each figure.

### Reporting summary

Further information on research design is available in the Nature Research Reporting Summary linked to this article.

## Supplementary information

Supplementary Information

Reporting Summary

## Data Availability

All data generated or analysed during this study are included in this published article (and its Supplementary information files). Source data are provided with this paper and contains uncropped western blot images, original histological images and the raw data with a transparent audit trail of how these data have been transformed, normalized and averaged thereby underpinning the final data shown in the final figures of the manuscript. Source data are provided with this paper.

## Source data

Source data Peer Review File
