# Supplementary material for: Insulin protects acinar cells during pancreatitis by preserving glycolytic ATP supply to calcium pumps
Source: Nat Commun. 2021 Jul 19;12:4386. doi: 10.1038/s41467-021-24506-w (PMC8289871; doi:10.1038/s41467-021-24506-w)
Supplement: Supplementary file 1 — Supplementary Information [file 41467_2021_24506_MOESM1_ESM.docx]

**Insulin protects acinar cells during pancreatitis by preserving glycolytic ATP supply to calcium pumps**

**Supplementary Information**

**Supplementary Methods**

***Ins2^Akita^ and PACIRKO mouse breeding and genotyping─*** All animal procedures and in vivo experiments were approved by the University of Michigan Institutional Animal Care and Use Committee (IACUC). The breeding of PACIRKO mice and feeder strains (Ela-Cre^ER/+^ and IR^lox/lox^) at the University of Manchester, which includes the administration of tamoxifen to induce induce insulin receptor deletion was approved by the Home Office Project licence (PPL number, P08B76E2B; PPL holder, Ian Townsend). Mice were fed standard laboratory chow and placed

in a 12:12 h light-dark cycle with free access to food and water. All experiments using Ins2^Akita^ mice were performed on male heterzygous mice at 6-7 weeks, as this was empirically determined to be the time of onset of consistent hyperglycaemia. Ins2^Akita^ mice were a kind gift from Peter Arvan (Department of Internal Medicine, Unversity of Michigan). Heterozygous males were genotyped using the following primers: oIMR1093-5’-tgctgatgccctggcctgct-3’; oIMR1094-5’-tggtcccacatatgcacatg-3’. These primers distinguish between wild type (WT) and Ins2^Akita^ alleles because they span the restriction site FNU4H1, which is absent in the Ins2^Akita^ mutation. Therefore, the PCR product directly digested by FNU4H1 yields a single 140 bp product from WT homozygotes, a longer 280 bp product from Ins2^Akita^ homozygotes and both PCR products (double band) from Ins2^Akita^ heterogygotes ([1](#_ENREF_1)).

PACIRKO mice were generated by crossing male heterozygous Elastase-Cre-ER mice (Ela-Cre^ER/+^) with female homozygous double floxed insulin receptor mice (IR^lox/lox^) ([2](#_ENREF_2)). The resultant offspring were genotyped for Cre, using the following primers: Cre-F-gcattaccggtcgatgcaacgagtgatgag; Cre-R-gagtgaacgaacctggtcgaaatcagtgcg. Cre-positive male mice (PACIRKO) were further back-crossed with female homozygous IR^lox/lox^ mice. The resultant second generation offspring were also genotyped for Cre and represented the experimental groups of PACIRKO (Cre-positive) and corresponding littermate IR^lox/lox^ control (Cre-negative). The PACIRKO line was maintained by continually backcrossing with IR^lox/lox^ and the Ela-Cre^ER/+^ line was maintained by continually backcrossing with C57BL/6 mice to maintain heterozygosity. IR^lox/lox^ mice were crossed with each other to maintain homozygosity and genotyped using the following primers: (IR-F-ctg-aat-agc-tga-gac cac-ag; IR-R- gat gtg cac ccc atg tct-g). These primers correspond to the loxP site and part of the IR which amplifies the floxed IR gene giving rise to a single larger 313bp band (homozygous IR^lox/lox^) and the non-floxed IR gene giving rise to a single smaller 279bp band (WT). Consequently, heterozygous IR^lox/lox^ gives rise to a double band (313bp and 279bp). The IR^lox/lox^ has been engineered such that exon 4 of the IR is flanked by two loxP sites which in the presence of activated Cre-recombinase results in deletion of exon 4 and a frame shift mutation and stop of translation. The final product if expressed at all, is a highly truncated non-functional N-terminal domain of the IR α-subunit lacking the insulin binding site, transmembrane and kinase domains. However, in the absence of activated Cre-recombinase the IR remains expressed normally in the offspring allowing them to survive and breed and allowing normal pancreatic development ([3](#_ENREF_3)). Crossing IR^lox/lox^ mice with mice expressing a Cre-ER fusion protein, driven by the pancreatic acinar-specific Elastase promoter, induces specific IR gene inactivation within pancreatic acinar cells. Cre-ER protein is a fusion protein between Cre-recombinase and the oestrogen receptor ([4](#_ENREF_4)) that is expressed in all pancreatic acinar cells but remains inactive until mice are fed tamoxifen which results in the Cre-recombinase activation. Both the experimental mice (PACIRKO) and age-matched littermate control mice (IR^lox/lox^) were administered tamoxifen for 4 days at 5-6 weeks to induce insulin receptor deletion in PACIRKO mice. Tamoxifen was administered either by daily oral gavage (75 mg/kg) or by tamoxifen feed (Envigo) formulated as 400 mg tamoxifen citrate per kg diet to provide ~40 mg/kg tamoxifen per day ([5](#_ENREF_5), [6](#_ENREF_6)). This was sufficient to induce maximum insulin receptor deletion and on day 7 mice were used for experimentation; either tissue was harvested and pancreatic acinar cells isolated or pancreatitis was induced *in vivo* using either caerulein or POA/ETOH. IR expression/deletion was confirmed by western blotting using an anti-IRβ antibody (Cell Signaling).

***Caerulein-induced experimental pancreatitis─*** Mice received eight hourly intraperitoneal (IP) injections of 50 μg/Kg caerulein per day over two consecutive days and were euthanized by CO_2_ asphyxiation followed by cervical dislocation 2 hours or 24 hours after the last caerulein injection. Blood was immediately collected for assessment of blood glucose and plasma amylase (Phadebas amylase test, Magle Life Sciences). Whole pancreas tissue was rapidly dissected, weighed and cut into sections for processing for histology (10 % formalin), RNA (RNA later, Thermofisher), protein (snap frozen in liquid nitrogen, prior to homogenisation in lysis buffer) and wet/dry weight ratio (weighed before and after drying in an oven at 90 ºC for 24 hours).

***Pancreatic acinar cell isolation─***Pancreatic acinar cells from IR^lox/lox^, PACIRKO and Ela-Cre^ER/+^ mice were isolated by collagenase-digestion ([7](#_ENREF_7), [8](#_ENREF_8)). Mice were humanely killed by an approved method as set out in Schedule 1 of the UK Animals Scientific Procedures Act 1986 (Certificate of Designation No 50/2506). The pancreas was quickly dissected and placed into ice cold HEPES-buffered physiological saline solution (HEPES-PSS; composition in mM: 137 Na^+^, 4.7 K^+^ 0.56 Mg^2+^, 1.28 Ca^2+^, 143.5 Cl^-^, 1 HPO_4_^2-^ and 10 HEPES, with a pH of 7.4), chopped with Noyes scissors and a scalpel and centrifuged for 2 minutes at 450 x g at 4°C. The supernatant was removed and the pelleted pancreas tissue sections were re-suspended in 10 ml of HEPES-PSS containing 0.15 mg.ml collagenase P (~200 U/ml) and 0.15 mg/ml of soybean trypsin inhibitor (Sigma) and incubated for 25 minutes with mechanical trituration every 5 minutes to break up the tissue into small cell clusters. Enzymatic dissociation was halted by the addition of 10ml of cold buffered washing solution (HEPES-PSS containing 5 % Fetal Bovine Serum (FBS)) and centrifuged. The pelleted cell clusters were resuspended in 7 ml of Dulbecco's Modified Eagle Medium (DMEM) containing 2.5 % FBS, and 0.15 mg/ml of trypsin inhibitor and filtered through a nylon mesh and cells were left to rest for 30 minutes at 37°C prior to any experimentation.

***RNA extraction and quantitative real time PCR─*** Pancreatic tissue stored at 4ºC in RNA later was transferred to TRIzol reagent (Ambion Life Technology) and homogenised using a polytron for 5-10 seconds. RNA was isolated using chloroform/isopropanol extraction and RNeasy spin column kit (Qiagen). Following quantification and assessment of purity using the Nanodrop 280/260nm optical density ratio (OD_280_/OD_260_ ratios), isolated RNA (200 ng) was reverse transcribed into cDNA using *Taq*Man reverse transcription reagents (Thermofisher) with random hexamers as primers ([9](#_ENREF_9)). Quantitative PCR reactions were carried out using the Absolute Blue SYBR Green ROX reagent (Thermo Scientific, Waltham, MA) with specific primers as previously described ([9](#_ENREF_9)).

***SDS PAGE and western blotting─*** Frozen tissue that had been snap-frozen in liquid nitrogen following dissection was homogenized and isolated pancreatic acinar cells were centrifuged (300 g for 5 minutes) and resuspended in lysis buffer (in mM: 50, Tris-HCl; 50, NaCl; 5, EDTA, 0.2 % triton X-100; 10mM NaF; 10, Na_4_P_2_O_7_; 25, glycerophosphate, 1, DTT; 1, PMSF; 0.2, Na_3_VO_4_; 10 µg/ml leupeptin; 10 µg/ml aprotinin). Lysates were allowed to solubilize at 4 °C for 30 minutes followed by centrifugation at 13,000 rpm for 10 minutes to remove insoluble debris (pellet). Sample protein was determined (Bradford assay, Bio-Rad Laboratories), denatured by boiling in SDS-Laemmli buffer for 5 minutes, separated using sodium dodecyl sulphate electrophoresis-polyacrylamide gel elctrophoresis (SDS-PAGE), transferred to nitrocellulose and western blotted using specific antibodies to insulin receptor (IRβ mAB; Cell Signalling) and amylase (rabbit anti-α-amylase; Sigma) phospho-Akt (p-Akt(ser473); Cell Signalling #9271) and the phosphor-PFKFB2 (p-PFKFB2(ser483) rabbit mAb; Cell Signalling #13064).

***Plasma amylase─*** Plasma was diluted 1:10 to 1:100 (adjusted to give a non-saturated absorbance reading) in assay buffer (0.9 % NaCl, 0.2 % BSA and 20 mM CaCl_2_) giving a final volume of 200 µl, which is then further diluted (1:20) in 4 ml of distilled water and brought to 37 °C in a shaking water bath (5 min). A single Phadebas reagent tablet was added to each sample tube, rapidly vortexed and incubated for a further 15 minutes at 37 °C prior to addition of 1 ml 0.5 M NaOH to terminate the reaction. Following centrifugation at 300 rpm for 5 minutes to pellet the insoluble Phadebas sediment, the resultant supernatant was transferred to glass tubes and the absorbance read at 620 nm on a spectrophotometer. Absorbance readings were converted to amylase concentration from a pre-determined standard curve.

***Histological assessment of pancreatitis─*** Pancreatic tissue was immersed in 10 % formalin immediately following dissection and incubated at 4ºC for 24 hours to allow tissue fixation. Fixed tissue was then immersed in 70 % ethanol prior to paraffin embedding, cutting into 5 μm sections and mounting onto slides. Tissue sections were stained with haemotoxylin and eosin (H&E) using a standard protocol by the University of Michigan Cancer Center Histology Core Facility. All pancreatic tissue sections were imaged, archived and analysed using the 3D-Histech Pannoramic-250 microscope slide-scanner (University of Manchester Bioimaging Facility) using a 40x/0.30 Plan Achromat objective (Zeiss) and CIS VCC FC60FR19CL colour camera. Snapshot images of the slide-scans were taken and processed using the Case Viewer software (3D-Histech). This allowed histological assessment of pancreatitis (pancreatic tissue injury) to be performed on whole tissue sections mounted on slides, rather than on selected “cherry-picked” magnified images. The entire sample region on each slide is imaged at 40x magnification and the individual images are stitched together so that different parts of tissue section can be zoomed in and out of in a "Google-Earth" like manner. Slides from all animal groups were graded by two independent, blinded observers according to severity and extent of oedema, inflammatory cell infiltration and acinar necrosis using a well validated histology injury score on a scale of 0-3 (where 3 was the most severe). The total score was the sum of the oedema, inflammation and necrosis scores for each slide (maximum score of 9). A more detailed description of each of these criteria and how they were graded have been previously described ([10-12](#_ENREF_10)).

***Immunohistochemistry of CD45*—**To further assess pancreatic tissue inflammation, immunohistochemistry using anti-CD45 antibody was perfromed on formalin-fixed parafin-embedded (FFPE) pancreatic tissue sections. Pancreatic FFPE tissue sections mounted on slides were heated at 55-60 ºC for 10 minutes followed by rehydration with xylene and then ethanol (95-100%). Antigen retrieval was achieved by sequecial heating/cooling with CITRA solution for 5 and then 3 minutes in a microwave followed by washing with water. Endogenous peroxidase was quenched by incubating in 0.6 % H_2_O_2_ in methanol for 15 minutes at room temperature. Tissue sections were then blocked in 2.5 % BSA/2 % normal goat serum in PBS for 30 minutes prior to incubation with the anti-CD45 antibody (1:50 dilution in blocking solution) at 4 ºC overnight, followed by sequential washes (in blocking solution) and subsequent incubation with immunoperoxidase-conjugated secondary antibody. Sections were then incubated with 3,3’-diaminobenzimide for 2-5 minutes, washed with water and then couterstained with heamatoxylin (30-45 minutes) prior to dehydration with ethanol and xylene and mounting with Permount mounting medium (Fisher Scientific).

***Imaging of fura-2 fluorescence─***Pancreatic acinar cells were loaded with 4 μM fura-2-AM (Invitrogen, Paisley UK) for 30 minutes at room temperature in HEPES-PSS as previously described ([7](#_ENREF_7)). Fura-2-loaded cells were imaged using an identical microscope/imaging system to previous studies ([7](#_ENREF_7)), which included a Nikon TE2000S microscope with 40x oil immersion SFluor objective lens, CoolSNAP HQ CCD camera (Photometrics, Tucson, AZ) and Cairn monochromator (Cairn Research, Kent, UK). MetaFluor imaging software (Molecular Devices, Downington, PA) controlled both image acquisition and analysis and background-subtracted fluorescence images were captured with 50 ms exposure and 5x5 binning every 5 s. The fura-2 fluorescence was calibrated into [Ca^2+^]_i_ using the well established method as previously described ([8](#_ENREF_8)). All experiments were carried out at room temperature (20-22^o^C).

***In situ Ca^2+^ clearance assay─***Cells were treated with the sarco/endoplasmic reticulum Ca^2+^-ATPase (SERCA) inhibitor, cyclopiazonic acid (CPA) in zero external Ca^2+^. This induces ER Ca^2+^ depletion and activation of store-operated Ca^2+^ entry (SOCE), such that addition of 20 mM external Ca^2+^ results in a rapid increase in [Ca^2+^]_i_. The subsequent removal of external Ca^2+^ (0 Ca^2+^, 1 mM EGTA) causes a rapid [Ca^2+^]_i_ clearance due predominantly to PMCA activity. Repeated Ca^2+^ influx-efflux phases allow POA to be applied during the second phase and [Ca^2+^]_i_ clearance rate (and thus PMCA activity) was quantified by measuring the linear rate from a standardised value of [Ca^2+^]_i_ during the second clearance phase and normalised to the corresponding linear rate during the first clearance phase ([7](#_ENREF_7)). This also circumvented the need to calibrate ratio into [Ca^2+^]_i_, which was not always possible due to the very high ratio vales reached following treatment with CPA and 20 mM Ca^2+^, which often exceeded the Rmax. The POA-induced change in this normalised rate was compared to corresponding time-matched control experiments. Under these conditions [Ca^2+^]_i_ clearance is almost exclusively due to PMCA activity. The paired experimental design controls for both cell-to-cell, and time-dependent, differences in PMCA activity (see Figure 4).

***Imaging magnesium green (MgGreen) fluorescence***—Pancreatic acinar cells were loaded with 4 µM MgGreen acetoxymethyl ester (AM) for 30 minutes at room temperature, simlar to previous studies ([13](#_ENREF_13), [14](#_ENREF_14)). MgGreen-loaded cells were imaged using the same fluorescence microscope used for assessing fura-2 and NADH autofluorescence, except cells were excited with light at 488 nm and emmitted light collected through a FITC dichroic. Background-subtracted images were acquired every 5 seconds and MgGreen fluorescence was quantified as raw fluorescence grey levels. Cells were first treated with the protonophore and mitochondrial uncoupler, CCCP (4 μM), to induce mitrochondrial ATP depletion. This was followed by treatment with iodoacetate (IAA, 2 mM), an inhibitor of the glycolytic enzyme, glyceraldehyde phosphate dehydrogenase (GAPDH), to induce glycolytic ATP depletion. Relative mitochondrial ATP depletion vs glycolytic ATP depletion was quantified as the CCCP-induced increase in MgGreen vs IAA-induced increase in MgGreen fluorescence, each normalised to the maximum change in MgGreen fluorescence.

***Measurement of glycolysis using the pH-Xtra Glycolysis Assay***— Acinar cell glycolysis was assessed by real-time, kinetic analysis of extracelullar acidification rate (ECAR) using a pH-Xtra Glycolysis Assay kit (Agilent) and a plate reader with time-resolved fluorescence (TRF) capability (CLARIOstar, BMG Labtech). Extracellular acidification is mainly due to lactic acid efflux, which results in a reduction in assay buffer pH. The pH Xtra probe sensitively detects this reduction in pH as an increase in sensor signal. Briefly, isolated mouse pancreatic acinar cells were washed three times with respiration buffer and pre-incubated in the presence or absence of 10 nM insulin for 30 min followed by the addition of 30 μM POA. After 15 minutes, 40 μl of cell suspension per condition was transferred to a 96 half area well plate and 10 μl of pH-Xtra reagent (fluorescent probe) was added to each well. Blank control wells were included and cell-free negative and positive (glucose oxidase) wells were used as signal controls. Each condition was carried out in triplicate. Both the metabolic incubations and the measurements were carried out at 37°C.

Dual read TRF lifetime detection mode was performed and the change in fluorescent lifetime from 100 to 300 µs delay and a 30-µs read window (excitation 340 ± 50 nm, emission 615 ± 18 nm) was monitored over time. The two TRF intensity readings collected were used to calculate the ratio-metric fluorescence lifetimes using a data analysis software (MARS, BMG Biotech), which applies the following formula: Lifetime (μs)[τ] = (D2-D1)/ln(IW1/IW2) where IW1 and IW2 represent the two (dual) measurement windows and D1 and D2 represent the delay time prior to measurement of W1 and W2, respectively. Lifetime values (μs) were corrected based on the blank wells and represent the extracellular acidification in each individual sample, then scaled to [H^+^] using the same software tool.

| **REAGENT or RESOURCE** | **SOURCE** | **IDENTIFIER** |
| --- | --- | --- |
| **Antibodies** | | |
| Anti-IRβ antibody (4B8) rabbit mAB | Cell Signaling Technology | Cat#3025 |
| Anti-CD45 (Rat anti-mouse) | BD Biosciences | Cat#550539 |
| Rabbit anti-α-amylase | Sigma-Aldrich | Cat#A8273 |
| Phospho-Akt (Ser473) | Cell Signaling Technology | Cat#9271 |
| Anti-Akt1 (C73H10) rabbit mAb | Cell Signaling Technology | Cat#2938 |
| Phospho-PFKFB2 (Ser483) rabbit mAb | Cell Signaling Technology | Cat#13064 |
| Anti-Actin mAB | Sigma-Aldrich | Cat#A3854-200UL |
| Anti-Cyclophilin-A | Cell Signaling Technology | Cat#2175 |
| Immobilized Phospho-Akt Substrate mAB | Cell Signaling Technology | Cat#9646 |
| Phospho-Akt Substrate mAb (HRP) | Cell Signaling Technology | Cat#6950 |
| Phospho-(Ser/Thr) Akt Substrate Antibody | Cell Signaling Technology | Cat#9611 |
| PKM2 (D78A4) XP® Rabbit mAb | Cell Signaling Technology | Cat#4053 |
| Pyruvate Dehydrogenase Rabbit mAb | Cell Signaling Technology | Cat#3205 |
| Hexokinase I (C35C4) Rabbit mAb | Cell Signaling Technology | Cat#2024 |
| LDHA (C4B5) Rabbit mAb | Cell Signaling Technology | Cat#3582 |
| PKM1/2 (C103A3) Rabbit mAb | Cell Signaling Technology | Cat#3190 |
| PFKP (D4B2) Rabbit mAb | Cell Signaling Technology | Cat#8164 |
| Anti-rabbit IgG, HRP-linked Antibody | Cell Signaling Technology | Cat#7074 |
| Anti-IRS1 Rabbit mAB | Invitrogen | Cat#MA5-15068 |
| Anti-IRS2 Mouse mAB | Millipore | Cat#MABS15 |
| Anti-IRS2 Rabbit mAB | Abcam | Cat#ab13410 |
| Anti-Phosphotyrosine Antibody clone 4G10 | Sigma-Aldrich | Cat#05-321 |
| Anti-rabbit IgG Antibody | Cell Signaling Technology | Cat#2729 |
| Dynabeads Protein G | Invitrogen | Cat#10003D |
| **Chemicals, Peptides, and Recombinant Proteins** | | |
| Tamoxifen | Sigma-Aldrich | Cat#T5648 |
| Tamoxifen diet (TAM Diet, 400 TC, 2016) | Envigo Teklad Diets | Cat#TD.130859 |
| Caerulein | Sigma-Aldrich | Cat#C9026 |
| Phadebas amylase test | Fisher Scientific | Cat#10121135 |
| RNA later | Thermofisher | Cat#AM7020 |
| Palmitoleic Acid | Sigma-Aldrich | Cat#P9417-1G |
| Collagenase-P | Roche Diagnostics | Cat#11213857001 |
| Soybean trypsin inhibitor | Sigma-Aldrich | Cat#Cat#T9128-1G |
| Bovine serum albumin (BSA, Fraction V) | Sigma-Aldrich | Cat#A3059-10G |
| BioRad protein assay reagent | BioRad | Cat#500-0006 |
| Fura-2-AM | Invitrogen | Cat#F1221 |
| Magnesium Green-AM | Invitrogen | Cat#M3735 |
| Cyclopiazonic acid (CPA) | Calbiochem | Cat#18172-33-3 |
| CCCP | Sigma-Aldrich | Cat#C2759 |
| Oligomycin | Sigma-Aldrich | Cat#75351-5MG |
| Bromopyruvate | Sigma-Aldrich | Cat#16490 |
| Iodoacetate | Sigma-Aldrich | Cat#I2512-25G |
| LY294002 (PI3 Kinase Inhibitor) | Cell Signaling Technology | Cat#9901 |
| Clear Bottom, White Walled 96-Well Plates | Corning Costar | Cat#3610 |
| Tris-HCl | Sigma-Aldrich | Cat#T5941-100G |
| Triton X-100 | Sigma-Aldrich | Cat#X100-500ML |
| DTT | Sigma-Aldrich | Cat#D9779 |
| PMSF | Sigma-Aldrich | Cat#P7626 |
| Leupeptin | Sigma-Aldrich | Cat#EI8 |
| Aprotinin | Sigma-Aldrich | Cat#A3428 |
| PhosSTOP | Roche | Cat#1873580001 |
| Complete EDTA Protease Inhibitor | Roche | Cat#104906837001 |
| AR Citra Plus Sol. PH-6.2 | X BioGenex | Cat#HK080-5K |
| Permount mounting medium | Fisher Scientific | Cat#15820100 |
| 3,3’-diaminobenzimide | Merck | Cat#HC867214 |
| TRIzol reagent (Ambion Life Technology) | Fisher Scientific | Cat#15-596-018 |
| RNase Away | Thermofisher | Cat#10328-011 |
| Chloroform | Sigma-Aldrich | Cat#C2432 |
| Isopropanol | Sigma-Aldrich | Cat#I-9516 |
| β-mercaptoethanol | Sigma-Aldrich | Cat#M-3148 |
| Critical Commercial Assays | | |
| ViaLight® Plus ATP kit (fire-fly luciferase | Lonza | Cat#LT17-221 |
| RNeasy spin column kit | Qiagen | Cat#74104 |
| TaqMan reverse transcription reagents | Thermofisher | Cat#N8080234 |
| RNase-free DNase set | Qiagen | Cat#79254 |
| Random hexamers | Thermofisher | Cat#N8080127 |
| ABsolute Blue SYBR Green ROX reagent | Thermo Scientific | Cat#AB4162B |
| pH-Xtra Glycolysis Assay | Agilent | Cat#PH200-4 |
| **Experimental Models: Organisms/Strains** | | |
| Ela-Cre^ER/+^ mouse (C57BL/6 background feeder strain, male) | Kind gift from Craig D Logsdon | University of Texas, MD Anderson Cancer Center |
| IR^lox/lox^ mouse (C57BL/6 background feeder strain, male) | Kind gift from C. Ronald Kahn | Joslin Diabetes Center, Harvard |
| PACIRKO mouse (C57BL/6 background strain, male) | In house breeding |  |
| Ins2^Akita^ mouse (C57BL/6 background, male) | Kind gift from Peter Arvan | University of Michigan |
| **Oligonucleotide Primers (Mouse genotyping)** | | |
| ***Ins2^Akita^ mutation***  oIMR1093-5’-tgctgatgccctggcctgct-3’;  oIMR1094-5’-tggtcccacatatgcacatg-3’. | Eurofins | N/A |
| ***Cre (identify Ela-Cre^ER/+^)***  Cre-F-gcattaccggtcgatgcaacgagtgatgag;  Cre-R-gagtgaacgaacctggtcgaaatcagtgcg | Eurofins | N/A |
| ***Floxed-IR (identify IR^lox/lox^ and PACIRKO)***  IR-F-ctg-aat-agc-tga-gac cac-ag  IR-R- gat gtg cac ccc atg tct-g | Eurofins | N/A |
| **Oligonucleotide Primers (qPCR)** | | |
| ***TNFα (pancreas tissue cytokine)***  TNFα-F-ctg-tag-ccc-acg-tcg-tag-c  TNFα-R-ttg-aga-tcc-atg-ccg-ttg | Invitrogen | Cat No: 10336022  275778D06 (primer#)  275778D07 (primer#) |
| ***IL-6 (pancreas tissue cytokine)***  IL-6-F-tcc-agt-tgc-ctt-ctt-ggg-ac  IL-6-R-gta-ctc-cag-aag-acc-aga-gg | Invitrogen | Cat No: 10336022  275778E06 (primer#)  275778E07 (primer#) |
| ***IL-1β (pancreas tissue cytokine)***  IL-1β-F-cca-gct-tca-aat-ctc-aca-gca-g  IL-1β-R-ctt-ctt-tgg-gta-ttg-ctt-ggg-atc | Invitrogen | Cat No: 10336022  275778E08 (primer#)  275778E09 (primer#) |
| ***Amylase-2 (pancreas tissue)***  Amylase-2-F-cct-tct-gac-aga-gcc-ctt-gtg  Amylase-2-R-gga-tga-tcc-tcc-agc-acc-at | Invitrogen | Cat No: 10336022  275778F05 (primer#)  275778F06 (primer#) |
| ***Elastase-1 (pancreas tissue)***  Elastase-1-F-ctg-aag-ccc-gga-gga-act-c  Elastase-1-R-tgg-tgc-cat-gat-cct-cca-ta | Invitrogen | Cat No: 10336022  275778E08 (primer#)  275778E09 (primer#) |

**Supplementary Results**

***Optimization of POA/ETOH-induced experimental pancreatitis***—The recently characterized palmitoleic acid/ethanol (POA/ETOH) model mimics pancreatitis induced by excessive alcohol and fat consumption in humans and mimics the downstream pathology ([10](#_ENREF_10)). In the original study that characterized this model, the combination of POA and ethanol was necessary as it was subsequently shown to be due to ethanol/fatty acid metabolite, POAEE ([10](#_ENREF_10)). In the current study we used two hourly injections of a cocktail of 100 mg/kg POA and 0.8 g/kg ethanol (60% ETOH:40% PBS ratio), which was a slightly lower dose of POA and ETOH used in the study that originally characterised this model ([10](#_ENREF_10)). In our hands we found that the “pure” ethanol that the POA was dissolved in produced considerable intraperitoneal fluid exudation and collatoral organ injury, including the necrosis of the intestines and liver, that we suspect was due to the direct toxicty of the ethanol, rather than the secondary consequence of pancreatitis. We therefore chose to reduce the ETOH concentration by mixing with PBS at a ratio 60% ETOH:40% PBS. This was the lowest concentration of ETOH to maintain the solubility of POA while producing minimal intraperitoneal fluid exudation and collateral organ injury and also produced minimal pancreatic injury. Accordingly, we tested the effect of IP injections of “pure” ethanol (1.35 g/Kg) and the “diluted” ETOH (0.8 g/Kg; 60% ETOH:40% PBS), both without POA, on histology and plasma amylase and compared responses to the corresponding cocktail with POA (Figure S1). “Pure” ethanol (1.35 g/Kg) induced some oedema and minor pancreatic tissue injury and significantly increased plasma amylase (Figure S1b), whereas injection of the lower dose of ethanol mixed with PBS (0.8 g/Kg; 60% ETOH:40% PBS) without POA produced minimal tissue injury (Figure S1c), compared to the combination of this lower dose of ETOH and POA (Figure S1d) and had no effect on plasma amylase (Figure S1f). This suggests that the adjusted dose regime of POA/ETOH (100 mg/Kg POA/0.8 g/Kg ethanol; 60:40% ETOH:PBS) produced a more specific pancreatitis, rather than ethanol-induced systemic inflammation due to peripheral organ injury independent of pancreatitis. Therefore, in all subsequent experiments utilizing this model mice received one IP injection containing PBS followed by two hourly IP injections of 100 µg/Kg POA, 0.8 g/Kg ethanol (ETOH).

***Pancreatic acinar cell amylase expression and secretion was reduced and pancreatitis-induced plasma amylase was blunted in PACIRKO mice—***Pancreatic tissue amylase mRNA expression was reduced, whereas Elastase mRNA expression was unaltered in PACIRKO mice (qPCR; Figure S2a). Similarly, at the protein level PACIRKO mouse pancreatic tissue amylase was significantly reduced to 57 ± 7 % compared to IR^lox/lox^ mouse pancreas (western blot; Figure S2b). This is consistent with previous studies, which also show that amylase is reduced in diabetic mice/rats ([15](#_ENREF_15), [16](#_ENREF_16)). Consequently, the caerulein-induced plasma amylase decreased from 20.2 ± 4.4 Uml^-1^ in IR^lox/lox^ mice to 8.6 ± 1.6 Uml^-1^ in PACIRKO mice (Figure S2d). Likewise the POA/ETOH-induced amylase decreased from 24.0 ± 3.9 Uml^-1^ in IR^lox/lox^ mice to 8.2 ± 0.7 Uml^-1^ in PACIRKO mice (Figure S2e).

**Justification for the use of PACIRKO vs IR^lox/lox^ mice for *in vivo* pancreatitis models*─***It is generally accepted that the expression of Cre-recombinase in any tissue may give rise to toxicity/injury, independent of the expresion of a floxed gene, due to non-specific excision of genomic DNA and thus DNA damage ([17](#_ENREF_17)). However, this was minimised in pancreatic acinar cells of PACIRKO mice by maintaining heterozygous Cre in the feeder Ela-Cre^ER+/^ mouse line, by continuously back-crossing with Cre-negative homozygous wildtype mice, and in the subsequent PACIRKO line by continuously back-crossing with Cre-negative homozygous IR^lox/lox^ mice. Nevertheless, it could be argued that even the heterozygous expression of Cre within the pancreas of PACIRKO mice could result in enhanced basal toxicity/injury or sensitize pancreatitis-induced acinar injury, independent of IR deletion.

***Ela-Cre^ER/+^ mice exhibit no overt pancreatic tissue/acinar cell injury and acinar cell signalling remains normal***—Based on the potential issues described above a number of additional control experiments were carried out to determine if the expression of Cre^ER^ in pancreatic acinar cells of Ela-Cre^ER/+^ mice induced any signs of pancreatic tissue injury or whether physiological signalling was altered or stress-induced responses were excacerbated in pancreatic acinar cells vs control mice. Histological analysis of pancreatic tissue, using H&E (Figure S3), revealed that there were no overt signs of injury in the pancreas of PACIRKO (Figure 3j & 4j) or Ela-Cre^ER/+^ mice (Figure S3b and S3d) and pancreas tissue appeared morphologically indistinguishable from the Cre-negative littermate (Ela-Cre^ER-/-^, C57 BL/6 background strain; Figure S3a and S3c) either with tamoxifen (Figure S3a and b) or without tamoxifen (Figure S3c and d). Moreover, CCK-evoked Ca^2+^ signalling (Figure S4) and downstream insulin-mediated signalling (Akt phosphorylation) (Figure 8) were also indistinguishable between acinar cells from of Ela-Cre^ER/+^ and IR^lox/lox^. Importantly however, using cellular models of pancreatitis, POA-induced cytotoxic Ca^2+^ overload (Figure S5) was indistinguishable between acinar cells from of Ela-Cre^ER+/^ (Figure S5a, e and f), IR^lox/lox^ (Figure S5c, e and f) and PACIRKO mice (Figure 5c, e and f). Furthermore, the insulin-induced protection that was observed in acinar cells from IR^lox/lox^ mice (Figure S5d, e and f) was also indistinguishable to that observed in acinar cells from of Ela-Cre^ER/+^ (Figure S5b, e and f) and only in cells from PACIRKO mice was this insulin-mediated protection abolished (Figure 5d, e and f). This therefore supports the central tenet that the more severe pancreatitis observed in PACIRKO mice was due to the deletion of IRs and the loss of endogenous insulin protection of acinar cells, rather than the non-specifc potentiation of cellular injury induced by Cre expression. Based on these results and in the interest of reducing the number of animals, experimental pancreatitis was only induced in PACIRKO and IR^lox/lox^ mice.

***Insulin-mediates a switch from mitochondrial ATP to glyolytic ATP as assessed using MgGreen****—*Fluorescence imaging of magnesium green (MgGreen)-loaded pancreatic acinar cells was used as an indirect measure of cellular ATP. This is because most cellular ATP exists as MgATP, therefore metabolic inhibitor induced ATP depletion causes an increase in free Mg^2+^ concentration and thus MgGreen fluorescence (Figure S6). Cells were first treated with the protonophore and mitochondrial uncoupler, CCCP (4 μM), to induce mitrochondrial ATP depletion. This was followed by treatment with iodoacetate (IAA, 2 mM), an inhibitor of the glycolytic enzyme, glyceraldehyde phosphate dehydrogenase (GAPDH), to induce glycolytic ATP depletion. As expected CCCP induced a 70 ± 3.7 % increase in MgGreen fluorescence, whereas IAA induced a 30 ± 3.7 % (n=5; Figure S6), consistent with a predomnant mitochondrial ATP production. However, insulin pre-treatment (10 nM for 15 minutes) reduced the CCCP-induced increase in MgGreen fluorescence to 32 ± 1.6 % (n=4; Figure S6), and thus mitochondrial ATP depletion, but enhanced the IAA-induced increase in MgGreen fluorescence to 68 ± 1.5 % (n=4; Figure S6), and thus glycolytic ATP depetion. These data show that insulin treatment switches pancreatic acinar cell metabolism towards glycolysis, which is sufficient to preserve cellular ATP to fuel the PMCA and maintain cytosolic [Ca^2+^]_i_ homeostasis.

***Insulin protects against the POA-induced inhibition of glycolysis****—*Glycolysis was assessed in pancreatic acinar cells using the pHXtra glycolytic flux assay (Agilent), a 96-well plate based time-resolved fluorescence assay of extracellular acidification rate (ECAR), due to lactic acid efflux, from isolated mouse pancreatic acinar cells in response to the pancreatitis-inducing agent POA with or without insulin treatment. The pH-Xtra reagent is a stable cell impermeant fluorescent reagent, added to suspensions of acinar cells, whose fluorescence increases with acidification over the biologically relevant range (pH 7.5-6.0). The pH-Xtra assay utilises a dual-read ratiometric time-resolved fluorescence lifetime measurement, which increases with acidification (Figure S7a), that is more stable with greater signal-to-noise ratio and dynamic range and is independent of light source brightness, background fluorescence photobleaching. Fluorescence lifetime can then be converted to pH (Figure S7b) using the MARS data analysis software (See Methods above) from which ECAR can be determined over the linear range using the same software tool, which is then normalized to control for each experiment (Figure S7c). Results show that insulin (10 nM) alone caused a marginal, but insignificant increase in ECAR (127 ± 14 % of untreated control). However, POA (30 μM) markedly reduced ECAR to 54 ± 6 % of untreated control cells (Figure S7), which was restored to simlar levels of control (96 ± 5 %) by pre-incubation with insulin (10 nM). These data further support the notion that insulin maintains glycolytic flux and thus ATP production to fuel the PMCA, even in the face of POA-induced metabolic crisis.

***PACIRKO mouse acinar cells exhibit marked over-expression and hyperphosphorylation of insulin receptor substrate (IRS) proteins—***PACIRKO mouse acinar cells exhibited a consistently high basal Akt and PFKFB2 phosphorylation. This was investigated further by western blotting and immunoprecipitation using IRS1 and IRS2 antibodies and phsophorylation assays using pan-phosphotyrosine antobody (Phospo-Tyr Ab). Western blotting revealed that both IRS1 and IRS2 expression was upregulated in PACIRKO (Figure S9a and S9b). In addition, immunoprecipitation of IRS1 or IRS2 followed by western blotting with phospho-Tyr Ab, revealed that IRS1 (Figure S9c) and IRS2 (Figure S9d) were both hyperphosphorylated. Furthermore, treatment of acinar cells isolated from IR^lox/lox^ mice with insulin (10 nM for as little as 1 minute), induced phosphorylation of IRS-1 (Figure S9c), although not IRS-2 (Figure S9d). Nevertheless, acinar cells isolated from PACIRKO mice remained insensitive to insulin despite the high basal IRS1 and IRS2 phosphorylation (Figure S9c and S9d). These data suggests that the high basal Akt and PFKFB2 were likely due to an upregulation of IRS-1 and IRS-2 expression and hyperphosphorylation upstream of Akt and PI3K.

**References**

1. Wang J, Takeuchi T, Tanaka S, Kubo SK, Kayo T, Lu D, Takata K, Koizumi A, and Izumi T. A mutation in the insulin 2 gene induces diabetes with severe pancreatic beta-cell dysfunction in the Mody mouse. *J Clin Invest.* 1999;103(1):27-37.

2. Sans MD AR, Vogel NL, D’Alecy LG, Kahn CR, Williams JA. . Specific deletion of insulin receptors on pancreatic acinar cells defines the insulin-acinar axis: implications for pancreatic insufficiency in diabetes. *Gastroenterology* 2011;140 (5):A233.

3. Kitamura T, Kahn CR, and Accili D. Insulin receptor knockout mice. *Annual review of physiology.* 2003;65(313-32.

4. Ji B, Song J, Tsou L, Bi Y, Gaiser S, Mortensen R, and Logsdon C. Robust acinar cell transgene expression of CreErT via BAC recombineering. *Genesis.* 2008;46(8):390-5.

5. Andersson KB, Winer LH, Mork HK, Molkentin JD, and Jaisser F. Tamoxifen administration routes and dosage for inducible Cre-mediated gene disruption in mouse hearts. *Transgenic research.* 2010;19(4):715-25.

6. Kiermayer C, Conrad M, Schneider M, Schmidt J, and Brielmeier M. Optimization of spatiotemporal gene inactivation in mouse heart by oral application of tamoxifen citrate. *Genesis.* 2007;45(1):11-6.

7. Baggaley EM, Elliott AC, and Bruce JI. Oxidant-induced inhibition of the plasma membrane Ca2+-ATPase in pancreatic acinar cells: role of the mitochondria. *Am J Physiol Cell Physiol.* 2008;295(5):C1247-60.

8. Bruce JI, and Elliott AC. Oxidant-impaired intracellular Ca2+ signaling in pancreatic acinar cells: role of the plasma membrane Ca2+-ATPase. *Am J Physiol Cell Physiol.* 2007;293(3):C938-50.

9. Sans MD, Lee SH, D'Alecy LG, and Williams JA. Feeding activates protein synthesis in mouse pancreas at the translational level without increase in mRNA. *Am J Physiol Gastrointest Liver Physiol.* 2004;287(3):G667-75.

10. Huang W, Booth DM, Cane MC, Chvanov M, Javed MA, Elliott VL, Armstrong JA, Dingsdale H, Cash N, Li Y, et al. Fatty acid ethyl ester synthase inhibition ameliorates ethanol-induced Ca2+-dependent mitochondrial dysfunction and acute pancreatitis. *Gut.* 2014;63(8):1313-24.

11. Van Laethem JL, Robberecht P, Resibois A, and Deviere J. Transforming growth factor beta promotes development of fibrosis after repeated courses of acute pancreatitis in mice. *Gastroenterology.* 1996;110(2):576-82.

12. Wildi S, Kleeff J, Mayerle J, Zimmermann A, Bottinger EP, Wakefield L, Buchler MW, Friess H, and Korc M. Suppression of transforming growth factor beta signalling aborts caerulein induced pancreatitis and eliminates restricted stimulation at high caerulein concentrations. *Gut.* 2007;56(5):685-92.

13. Mankad P, James A, Siriwardena AK, Elliott AC, and Bruce JI. Insulin protects pancreatic acinar cells from cytosolic calcium overload and inhibition of the plasma membrane calcium pump. *J Biol Chem.* 2012;287(3):1823-36.

14. Samad A, James A, Wong J, Mankad P, Whitehouse J, Patel W, Alves-Simoes M, Siriwardena AK, and Bruce JI. Insulin protects pancreatic acinar cells from palmitoleic acid-induced cellular injury. *J Biol Chem.* 2014;289(34):23582-95.

15. Korc M, Iwamoto Y, Sankaran H, Williams JA, and Goldfine ID. Insulin action in pancreatic acini from streptozotocin-treated rats. I. Stimulation of protein synthesis. *Am J Physiol.* 1981;240(1):G56-62.

16. Korc M, Owerbach D, Quinto C, and Rutter WJ. Pancreatic islet-acinar cell interaction: amylase messenger RNA levels ar determined by insulin. *Science.* 1981;213(4505):351-3.

17. Schmidt-Supprian M, and Rajewsky K. Vagaries of conditional gene targeting. *Nature immunology.* 2007;8(7):665-8.

**Supplementary Figures**

**
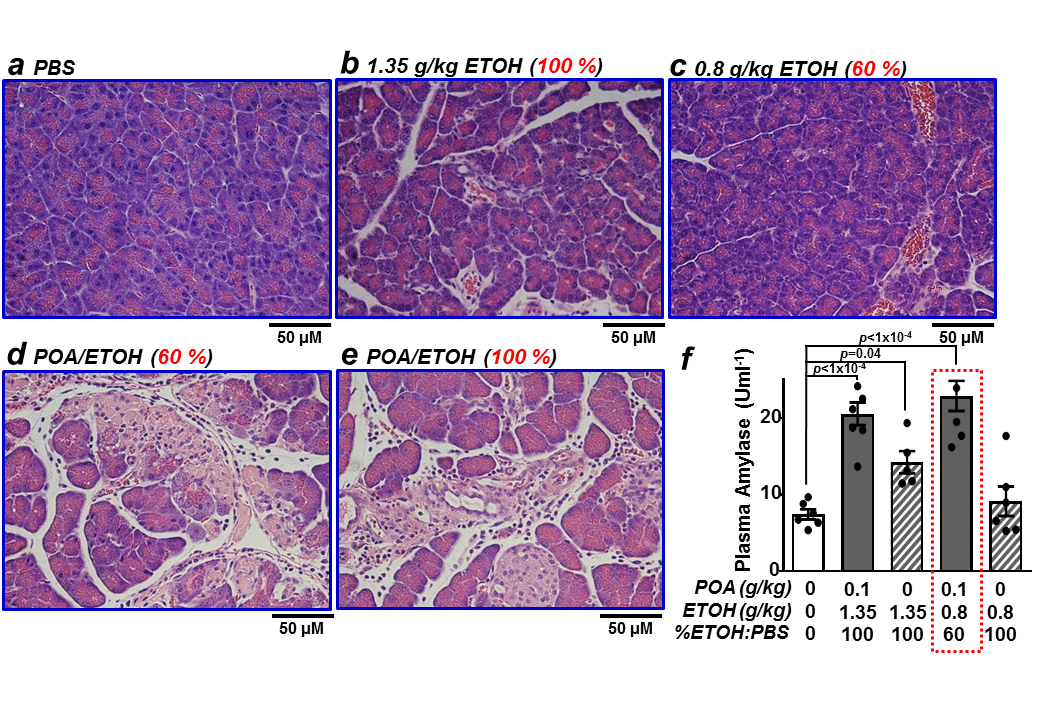
**

**Figure S1. Optimization of POA/ethanol (ETOH) dose regime for inducing acute pancreatitis. *a-e***. Haematoxylin and eosin (H&E) stained pancreatic tissue sections from C57BL/6 mice prepared 24 hours after receiving either 2 hourly intraperitoneal (IP) injections of phosphate-buffered saline (***a***., PBS), “pure” (100 %) ethanol at a dose of 1.35 g/kg ethanol (***b***., normally used as a vehicle for POA), “reduced” ethanol (60 % ETOH diluted with 40 % PBS) at a dose of 0.8 g/kg (***c***. to reduce collateral intraperitoneal organ damage) and a combination of 100 mg/kg POA with “pure” ethanol (***d***) or “reduced” ethanol (**e**). ***f***. Corresponding plasma amylase 2 hours after the last injection following each dose regime of POA/ETOH. Red dashed box represents the optimized dose regime (100 mg/kg POA: 0.8 g/kg ETOH) which produced a more specific pancreatic injury that was independent of ethanol-induced toxicity and any confounding effects of collateral peritoneal organ injury. Group sizes were: PBS, n=6; 100 mg/kg POA in “pure” ETOH (1.35 g/kg), n=7; “pure” ETOH (1.35 g/kg) alone, n=5; 100 mg/kg POA in “reduced” ETOH (0.8 g/kg), n=7; “reduced” ETOH (0.8 g/kg) alone, n=6. Significance determined by one-way ANOVA with Sidak’s multiple comparisons.


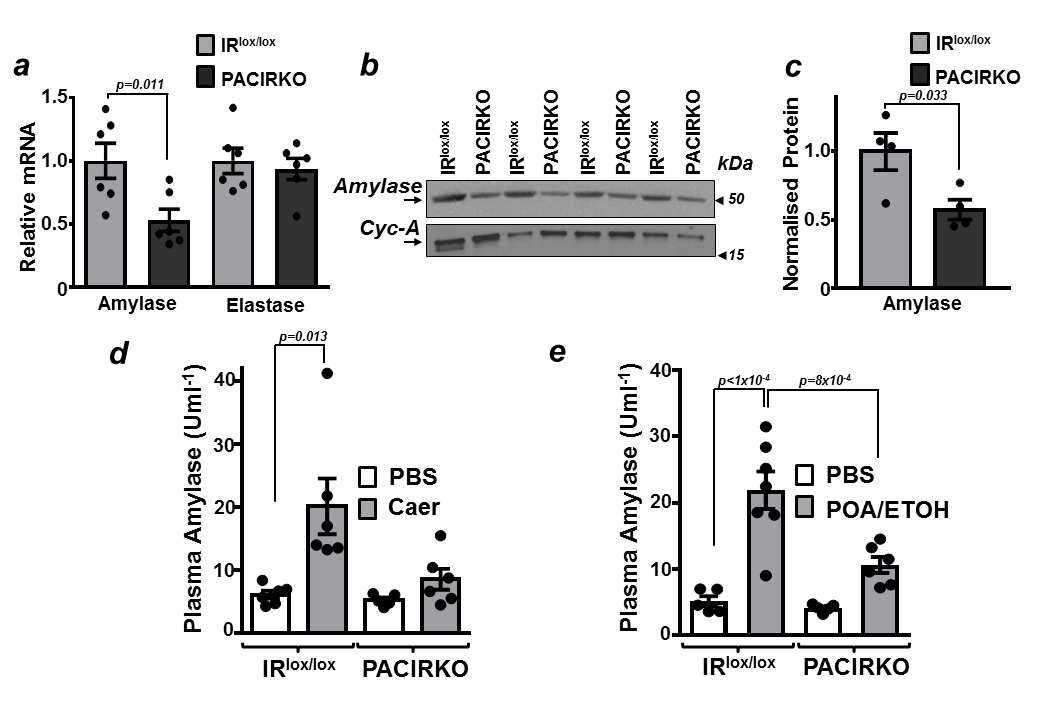


**Figure S2. Pancreatic amylase expression is reduced and caerulein and POA/ethanol-induced increase in plasma amylase is blunted in PACIRKO mice. *a.*** pancreatic amylase expression was reduced at the mRNA level (assessed by qPCR) in PACIRKO vs IR^lox/lox^ mice, whereas elastase mRNA was expression was the same in PACIRKO and IR^lox/lox^ mice (***a***., mean relative mRNA (± SEM), n=6 for all groups). Significance determined by ANOVA with Sidak’s multiple comparisons. Pancreatic amylase expression was also reduced at the protein level in PACIRKO vs IR^lox/lox^ mice (assessed by western blot with cyclophilin-A used as a loading control, ***b***., mean relative protein (± SEM), n=4 for each group). This was quantified by normalising the amylase band intensity to its corresponsing cyclophilin-A band intensity, which was then further normalised to the average value for IR^lox/lox^ mice (***c***; n=4 for PACIRKO and IR, significance determined by two-tailed t-test. The western blot for amylase and corresponding loading control (cyclophilin-A) was from the same gel (membrane cut and incubated with corresponding antibody). Measurement of plasma amylase 2 hours after the last IP injection of either caerulein (***d,*** Caer) or POA/ethanol (***e,*** POA/ETOH) vs PBS alone in either IR^lox/lox^ mice or PACIRKO mice. Group sizes were: IR^lox/lox^ PBS, n=6; IR^lox/lox^ Caer, n=6; PACIRKO PBS, n=5; PACIRKO Caer, n=6; IR^lox/lox^ PBS, n=5; IR^lox/lox^ POA/ETOH, n=7; PACIRKO PBS, n=5; PACIRKO POA/ETOH, n=6; Significance determined by either Kruskal Wallis with Dunn’s multiple comparisons (***d,*** Caer) or one way ANOVA with Sidak’s multiple comparisons (***e,*** POA/ETOH).


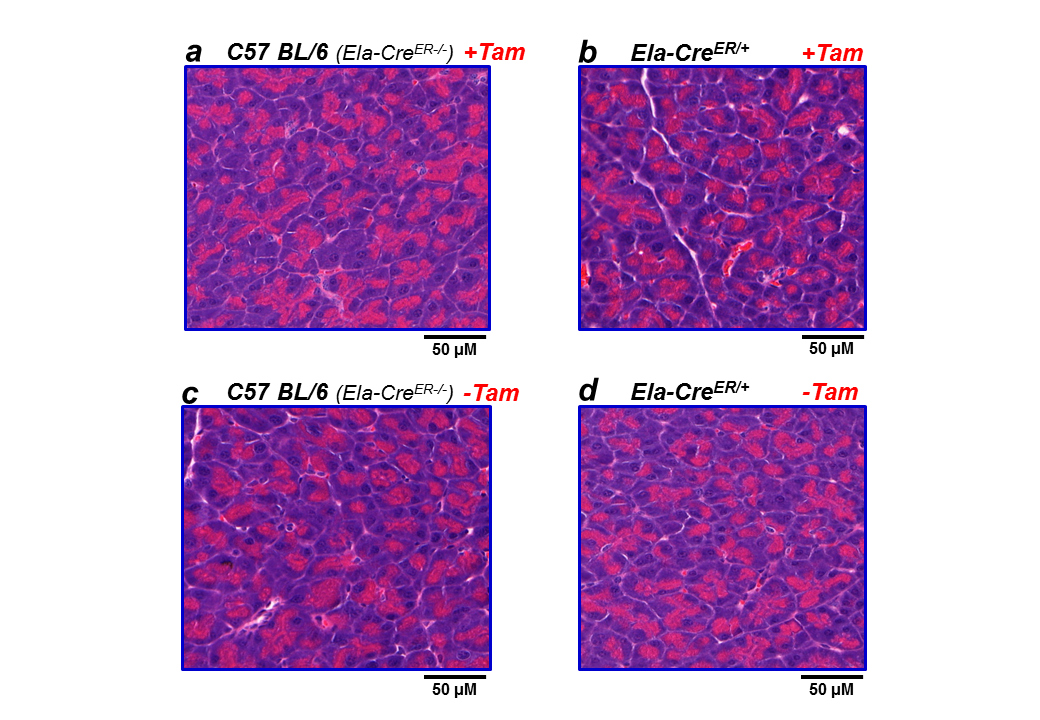


**Figure S3. Ela-Cre^ER/+^ mice exhibit no overt histological evidence of pancreatic tissue injury and is indistinguishable from Cre-negative littermate C57 BL/6 mice.** Haematoxylin and eosin (H&E) stained pancreatic tissue sections from C57BL/6 mice (**a** and **c**) and Ela-Cre^ER/+^ mice (**c** and **d**) following treatment with (**a** and **b**) or without tamixifen (**c** and **d**) to induce activation of Cre-recombinase. Histological images are representative of at least 4 mice for each group.


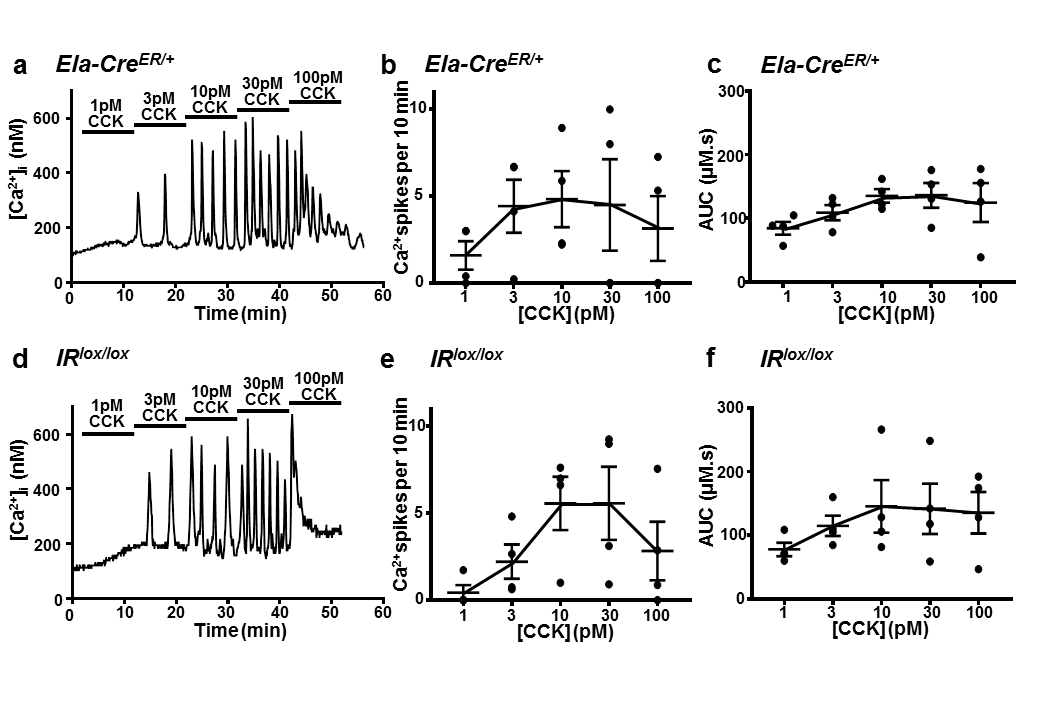


**Figure S4. CCK-evoked [Ca^2+^]_i_ signalling is indistinguishable between acinar cells from Ela-Cre^ER+/^ vs IR^lox/lox^ mice.** Representative traces of CCK-evoked [Ca^2+^]_i_ oscillation from acinar cells from Elas-Cre^ER/+^ (***a***) and IR^lox/lox^ mice (***d***) treated with increasing concentrations of CCK (1-100 pM). Corresponding average concentration-response graphs for frequency of CCK-evoked [Ca^2+^]_i_ oscillations (***b*** and ***e***) and area under the curve (AUC; ***c*** and ***f***). Data were derived from individual values from multiple cells (9-16) in the filed of view for each experiment. These values were averaged giving the experimental mean, that were in turn averaged across 4 separate experiments for each condition giving the true mean ± SEM as indicated in ***b*** and ***c*** (Ela-Cre^ER+/^ mice) and ***e*** and ***f*** (IR^lox/lox^ mice).


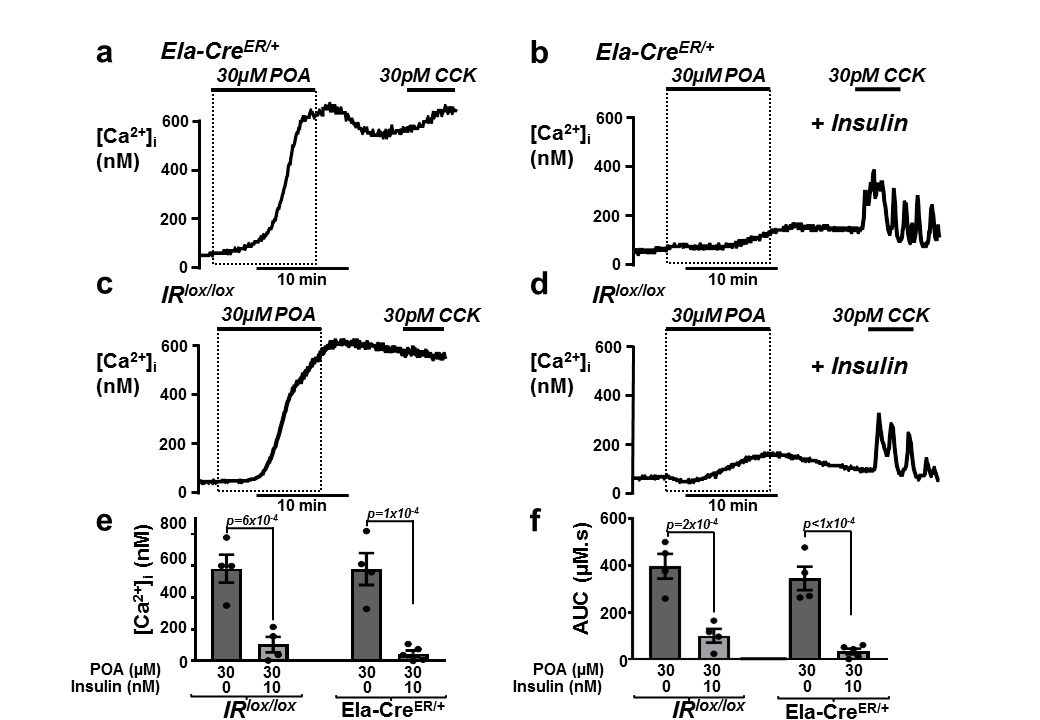


**Figure S5.** **Palmitoleic-induced Ca^2+^** **overload and insulin-mediated protection is indistinguishable between pancreatic acinar cells from Ela-Cre^ER/+^ and IR^lox/lox^ mice**. Representative traces showing POA-induced [Ca^2+^]_i_ responses (***a***–***d***) in untreated fura-2-loaded pancreatic acinar cells (***a*** and ***c***) and following pre-treatment with 10 nM insulin for 15 min (***b*** and ***d***) from Ela-Cre^ER/+^ (***a*** and ***b***) and IR^lox/lox^ mice (***c*** and ***d***). Cells were also subsequently treated with 30 pM CCK to test for recoverability and thus cell viability post-POA treatment. Mean (±SEM) maximum increase in resting [Ca^2+^]_i_ above baseline (***e***) and mean (±SEM) area under the curve (AUC; ***f***) over the treatment and recovery period in the absence (*dark grey box*) or following treatment with 10 nM insulin (*light grey box*). Significance (specifc p values as indicated) was determined by one way ANOVA with Sidak’s multiple comparisons (n=4 separate experiments for each experimental condition, except for Ela-Cre^ER/+^ with POA and Insulin, n=5). Data were derived from individual values from multiple cells (6-36) in the filed of view for each experiment. These values were averaged giving the experimental mean, that were in turn averaged across multiple experiments giving the true mean ± SEM as indicated in ***e*** and ***f***.


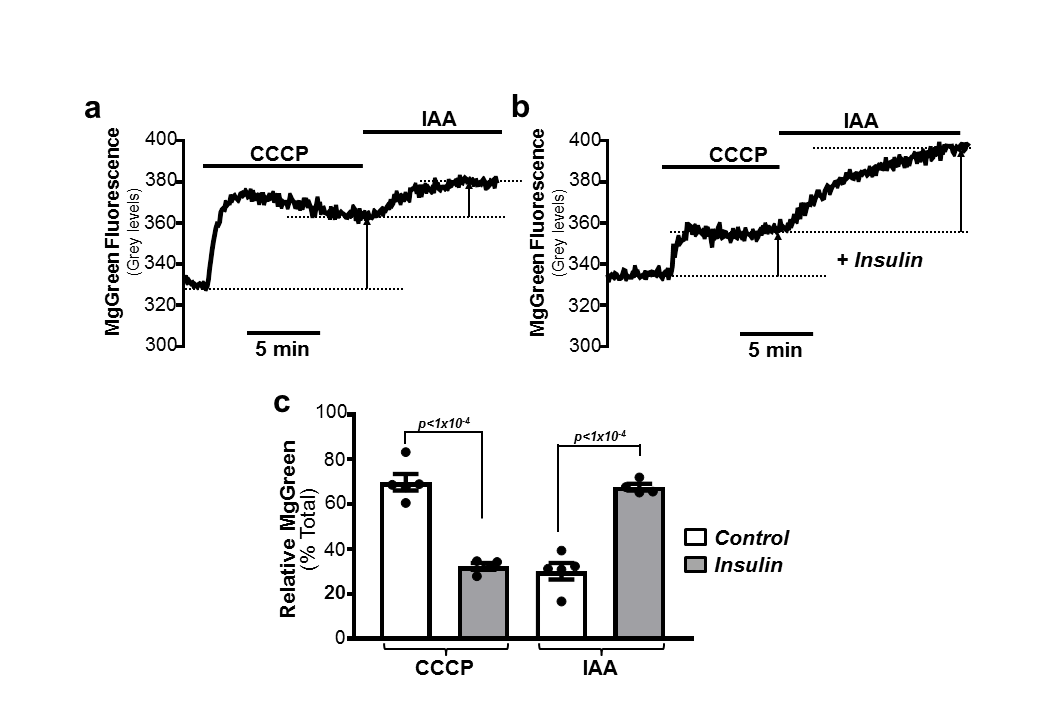


**Figure S6. Insulin-mediated switch from mitochondrial metabolism to glycolysis as assessed by MgGreen fluorescence.** MgGreen-loaded pancreatic acinar cells were treated with the protonophore and mitochondrial uncoupler, CCCP (4 μM), to induce mitochondrial ATP depletion (and corresponding increase in MgGreen fluorescence) followed by the GAPDH inhibitor, IAA (2mM), to induce glycolytic ATP depletion (and thus a further increase in MgGreen fluorescence) in untreated control cells (**a**) and insulin-treated cells (**b**, 10 nM for 15 minutes). The relative mitochondrial vs glycolytic ATP was quantified by normalising the CCCP vs IAA-induced increase in MgGreen fluorescence to the maximum increase in MgGreen fluorescence, respectively (**c.,** mean relative MgGreen fluoresence ± SEM). Significance (specifc p values as indicated) was determined by one way ANOVA with Sidak’s multiple comparisons from 5 separate experiments from control acinar cells (**a**) and 4 separate experiments from insulin-treated acinar cells (**b**). Data were derived from individual values from multiple cells (3-17) in the filed of view for each experiment. These values were averaged giving the experimental mean, that were in turn averaged across multiple experiments giving the true mean ± SEM as indicated in ***c***.


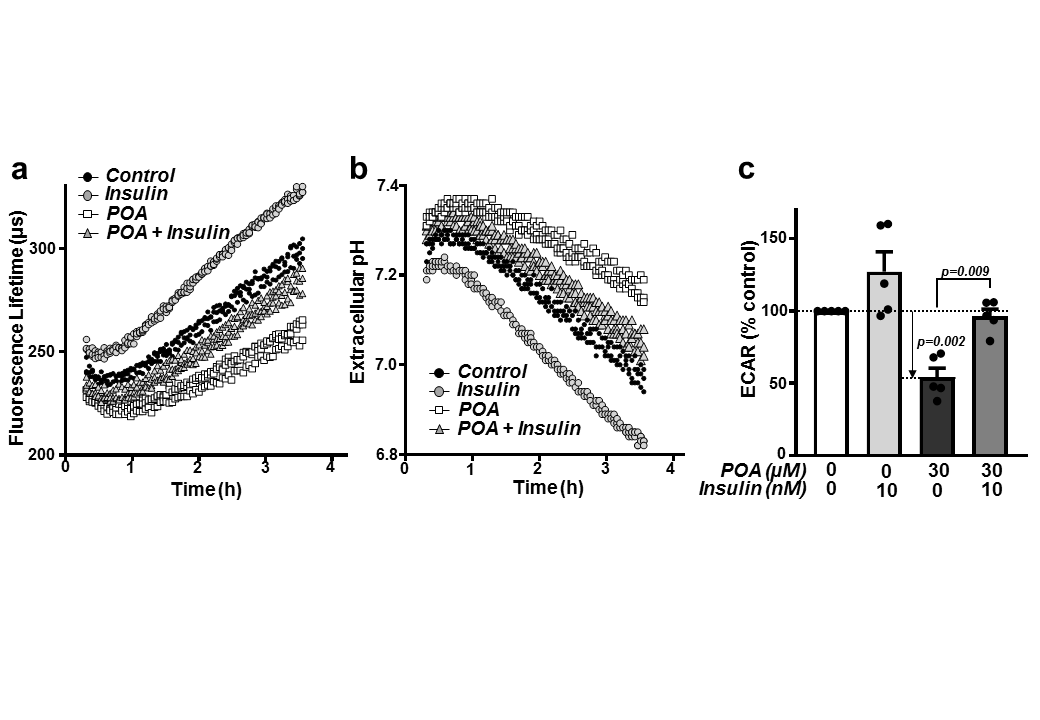


**Figure S7.** **Insulin protects against the POA-induced decrease in glycolytic flux.** Glycolysis was assessed using the pH-Xtra Glycolysis assay. **a**., Representative Extracellular Acidification (ECA) profile (3 replicates) showing lifetime signal curves calculated from dual-read time-resolved fluorescence measurements over 3 hours from cells treated with insulin (10 nM), POA (30 μM) or a combination of insulin and POA vs untreated control acinar cells. **b**, Representative experimental trace (3 replicates) showing lifetime values converted to pH scale using the data analysis software MARS. **c**., Mean extracellular acidification rate (ECAR ± SEM) from 5 separate experiments performed in triplicate for each condition; control, insulin alone, POA alone and a combination of insulin and POA. Replicate values were averaged to give the “experimental mean”, which were further averaged across experimental repeats to give the true mean ECAR (± SEM) reported in **c.** Lifetime values were converted to H^+^ scale using the data analysis software MARS and the slope was calculated using the linear portion of the curves and normalised to the control for each experiment. Significance was assessed by a two-tailed one sample t-test to determine differences relative to control (POA, p=0.002) and one-way ANOVA with Tukey’s multiple comparisons test (POA vs POA with insulin; p=0.009).


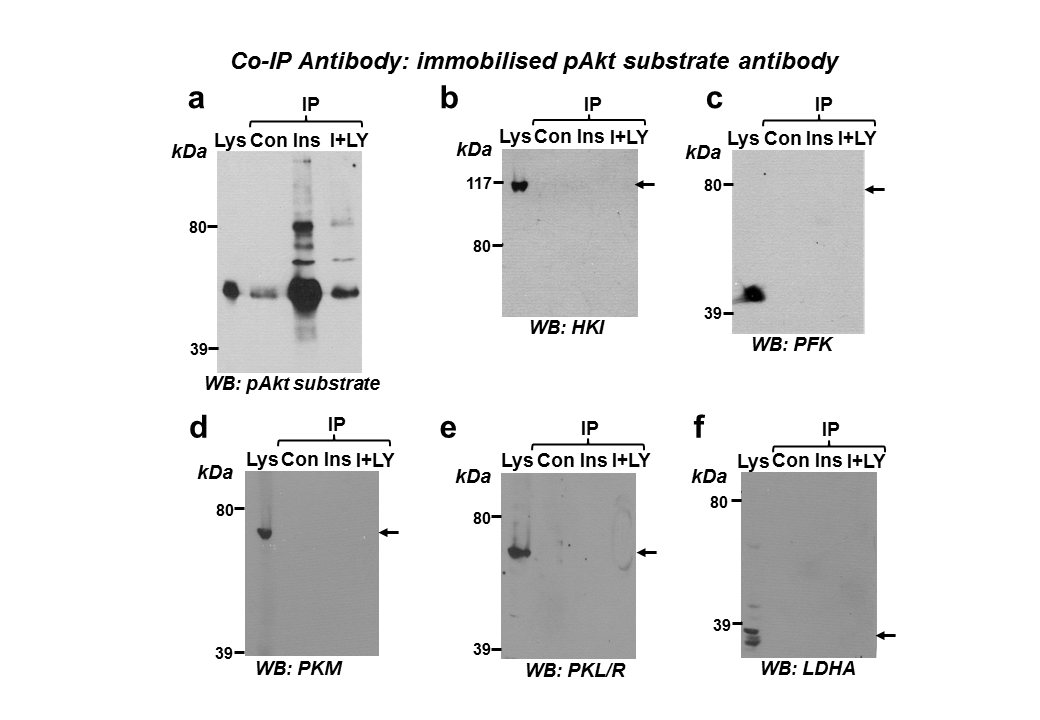


**Figure S8.** **Effect of insulin treatment on Akt-mediated phosphorylation of numerous glycolytic enzymes in pancreatic acinar cells.** Acinar cells were treated with and without insulin (10 nM) and/or the PI3K inhibitor, LY294002 (10 μM) for 15 minutes, followed by cell lysis. All Akt-mediated phosphorylated proteins were immunoprecipitated using immobilised pAkt substrate antibody, separated by SDS-PAGE and western blotted using either pAkt substrate antibody (***a***, positive control) or numerous glycolytic enzymes including, pyruvate kinase-M (PKM, ***b***), pyruvate kinase-L/R (PKL/R, ***c***), phosphofructokinase-1 (PFK-1, ***d***) hexokinase-1 (HK-1, ***e***) and lactate dehydrogenase-A (LDHA, ***f***). Blots are representative of three experimental repeats.

**
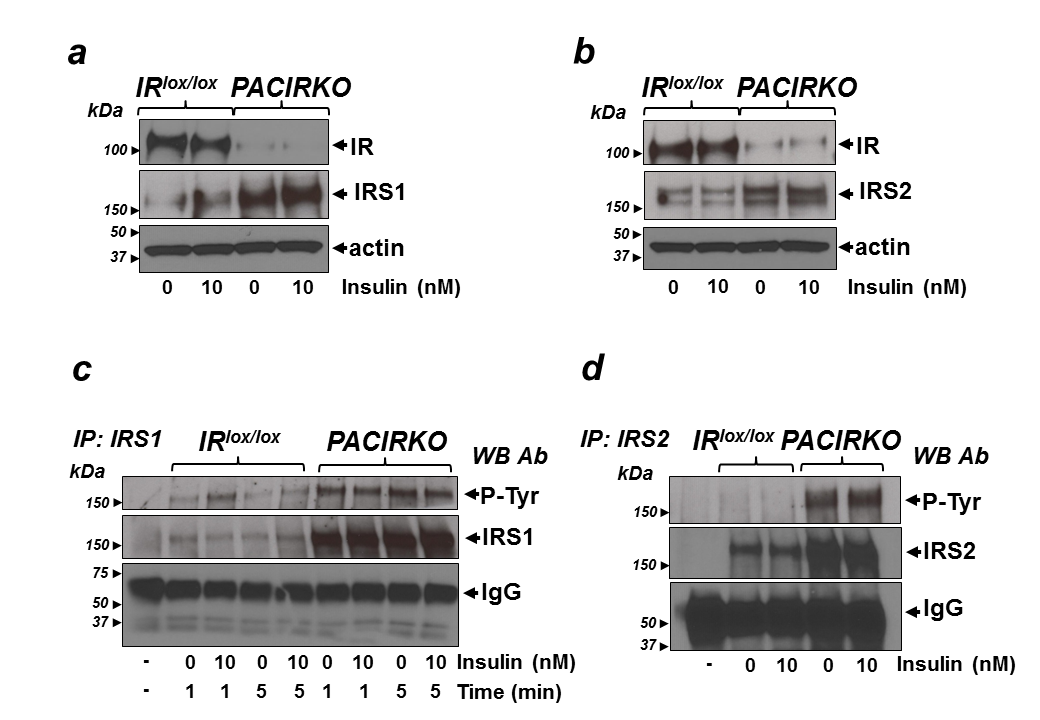
**

**Figure S9. Insulin receptor substrate (IRS) proteins are overexpressed and hyperphosphorylated in PACIRKO mouse acinar cells.** Pancreatic acinar cells from IR^lox/lox^ or PACIRKO mice were treated with or without 10 nM insulin for 1-5 minutes (**c**) or 5 minutes (**a, b** and **d**) followed by cell lysis. Protein lysates were either separated by SDS-PAGE and western blotted using antibodies for the insulin receptor (IR), insulin receptor substrate-1 (IRS1, **a**), insulin receptor substrate-2 (IRS2, ***b***), or actin as a loading control. Protein lysates were incubated with immunoprecipitating antibodies (IRS1, **c**; IRS2, **d**) and western blotted using antibodies for pan-phospho-tyrosine (P-Tyr) and IRS1 (**c**) or IRS2 (**d**). IgG heavy chain was used as a loading control. For each representative experiment shown (***a***-***d***) separate gels were run and each membrane cut and incubated with each corresponding antibody either in parallel or in series, including IR, IRS1 or IRS2 and the loading control actin (***a*** and ***b***) or p-Tyr, IRS1 or IRS2 with IgG heavy chain acting as the loading control (***c*** and ***d***). These were all sufficiently separated so that they could be resolved on the same gel. Each experiment shown (***a***-***d***) is representative of at least three independent experiments.


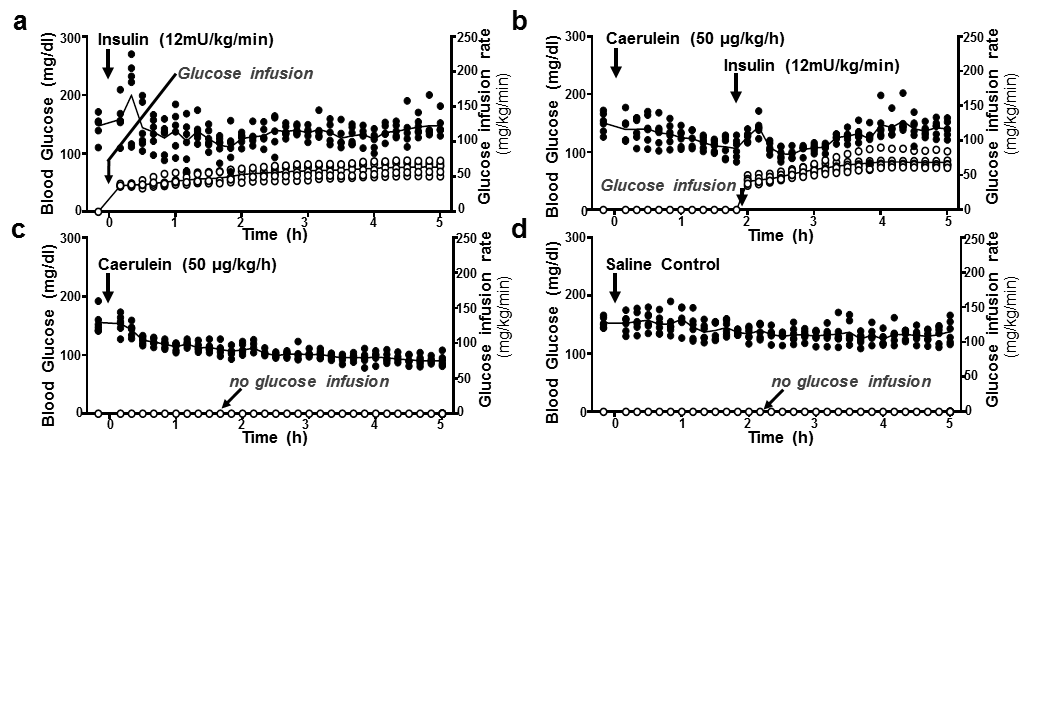


**Figure S10. Blood glucose and glucose infusion during caerulein infusion-induced pancreatitis combined with the hyperinsulinaemic euglycaemic clamp.** C57BL/6 mice were catherized via the carotid artery and jugular vein under recover anaesthesia to allow the continuous infusion of caerulein (to induce acute pancreatic injury), insulin (12 mU/kg/min) and glucose (variable rate) to maintain euglycaemia. Caerulein-induced acute pancreatic injury was experimentally induced by continuous infusion of caerulein (50 μg/kg/h) over 5 hours and plasma amylase was assessed as an early and immediate readout of pancreatic injury during the course of the experiment and at the end (figure 9). Mice were separated into 4 groups of 6 mice receiving insulin alone (**a**), caerulein and insulin (**b**, insulin added 2 hours into the caerulein infusion), caerulein alone (**c**) or saline control (**d**). Moment-to-moment monnitoring of blood gluocse and the glucose infusion rate required to ensure euglycaemia under each experimental condition. These data are identical to figure 9a-d except individual data points are presented as a dot plot with lines connecting the mean.
