# Supplementary material for: Insulin protects acinar cells during pancreatitis by preserving glycolytic ATP supply to calcium pumps
Source: Nat Commun. 2021 Jul 19;12:4386. doi: 10.1038/s41467-021-24506-w (PMC8289871; doi:10.1038/s41467-021-24506-w)
Supplement: Supplementary file 2 — Reporting Summary [file 41467_2021_24506_MOESM2_ESM.pdf]

nature research

Corresponding author(s): Jason Bruce

Last updated by author(s): May 18, 2021

Reporting Summary

Nature Research wishes to improve the reproducibility of the work that we publish. This form provides structure for consistency and transparency in reporting. For further information on Nature Research policies, see Authors & Referees and the Editorial Policy Checklist .

Statistics

For all statistical analyses, confirm that the following items are present in the figure legend, table legend, main text, or Methods section.

n/a	Confirmed	
☐	☒	The exact sample size (*n*) for each experimental group/condition, given as a discrete number and unit of measurement
☐	☒	A statement on whether measurements were taken from distinct samples or whether the same sample was measured repeatedly
☐	☒	The statistical test(s) used AND whether they are one- or two-sided<br>*Only common tests should be described solely by name; describe more complex techniques in the Methods section.*
☐	☒	A description of all covariates tested
☐	☒	A description of any assumptions or corrections, such as tests of normality and adjustment for multiple comparisons
☐	☒	A full description of the statistical parameters including central tendency (e.g. means) or other basic estimates (e.g. regression coefficient) AND variation (e.g. standard deviation) or associated estimates of uncertainty (e.g. confidence intervals)
☐	☒	For null hypothesis testing, the test statistic (e.g. *F*, *t*, *r*) with confidence intervals, effect sizes, degrees of freedom and *P* value noted<br>*Give P values as exact values whenever suitable.*
☒	☐	For Bayesian analysis, information on the choice of priors and Markov chain Monte Carlo settings
☒	☐	For hierarchical and complex designs, identification of the appropriate level for tests and full reporting of outcomes
☒	☐	Estimates of effect sizes (e.g. Cohen's *d*, Pearson's *r*), indicating how they were calculated

Our web collection on statistics for biologists contains articles on many of the points above.

Software and code

Policy information about availability of computer code

Data collection	All qPCR data were acquired using the Bio-Rad CFX96 Real Time Thermal Cycler System with Bio-Rad CFX Manager 3.1. All histological images were acquired using the 3D-Histech Pannoramic-250 microscope slide-scanner with 3D-Histech Case Viewer software (version 2.4). All fluorescence imaging data were acquired and analysed using MetaFluor imaging software (version 6.3r1, Molecular Devices, Downington, PA). ATP-based luminescence data were acquired using the BioTek® Synergy HT plate reader with Gen 5 software. Fluorescence lifetime and ECAR data were acquired, processed and analyzed by MARS software (BMG Biotech).
Data analysis	All data were analyzed, transformed and figures generated using Graphpad prizm v7 and Microsoft Excel 2010.

For manuscripts utilizing custom algorithms or software that are central to the research but not yet described in published literature, software must be made available to editors/reviewers. We strongly encourage code deposition in a community repository (e.g. GitHub). See the Nature Research guidelines for submitting code & software for further information.

Data

Policy information about availability of data

All manuscripts must include a data availability statement. This statement should provide the following information, where applicable:

- Accession codes, unique identifiers, or web links for publicly available datasets
- A list of figures that have associated raw data
- A description of any restrictions on data availability

All raw data pertaining to the figures shown in the manuscript (including supplementary data) have been included as a supplementary Source Data file (Excel). This file contains uncropped western blot images, original histological images and the raw data with a transparent audit trail of how these data have been transformed, normalized and averaged thereby underpinning the final data shown in the final figures of the manuscript.

Field-specific reporting

Please select the one below that is the best fit for your research. If you are not sure, read the appropriate sections before making your selection.

☒ Life sciences ☐ Behavioural & social sciences ☐ Ecological, evolutionary & environmental sciences

For a reference copy of the document with all sections, see nature.com/documents/nr-reporting-summary-flat.pdf

Life sciences study design

All studies must disclose on these points even when the disclosure is negative.

Sample size	Sample sizes were not predetermined by statistical methods of power calculation. For pancreatitis experiments, sample size was determined by availability of genetically modified mice and taking into account local ethical and reductionist animal use. For these experiments, group size was 3-7 mice and for most readouts of pancreatitis this was sufficient to reach statistical significance. For in vitro experiments pancreatic acinar cells were isolated from both PACIRKO and control mice (IRlox/lox and/or Ela-CreER/+ or C57BL/6 mice) on any given day. For each day, experiments included where possible time-matched controls, test treatments (POA) with and without insulin (POA and insulin). This was repeated for at least 4 and as high as 7 independently repeated experiments on 4 separate cell preparations for each condition to give a full balanced experimental design and to reduce as much as logistically possible any covariation. For these in vitro experiments we followed standard practices in the field, and we believe this to be sufficient, as our estimates of the mean and SEM did not deviate by incorporating more experiments. The sample size for each in vivo experiment and the number of individual experimental repeats for the in vitro cellular based experiments, including numbers of cells per experiment (technical replicates), have been listed in the corresponding figure legends.
Data exclusions	No data were excluded from analyses
Replication	All attempts at replication succeeded. In general, our data show mean +/- SEM from 3-7 independent experiments (biological replicates) sometimes performed on the same day but only when every other condition has been satisfied from that given cell preparation/mouse. For any given imaging experiment there might be between 2-20 cells in the field of view and therefore individual cell responses were averaged across that field of view and averaged to give the experimental mean. These means were further averaged to give the real mean (±SEM). All figures show individual data points scattered around the mean with error bars representing SEM.
Randomization	For all in vivo experiments animals were randomly allocated into either treatment or control group but also based on their genotype from any given litter. Similarly, for in vitro cellular-based experiments it was not possible to completely randomise group allocations as genotyping of animals was always predetermined. This was always necessary, particularly for in vitro cellular-based experiments to ensure that on any given day sufficient numbers of experiments could be performed on cell preparations from at least one PACIRKO mouse and one corresponding littermate control IRlox/lox mouse. Where it was logistically possible on any given day, control experiments (both negative, positive or time-matched controls), experimental manoeuvre (e.g. POA treatment), with and without insulin treatment, were performed on cells from both PACIRKO mice and IRlox/lox mice. This minimized as much as possible any co-variation.
Blinding	For histological analysis slides from all animal groups were graded by two independent, blinded observers. For all other experiments, similar to randomization, it was not possible for group allocation to be completely blinded as genotyping of animals was always predetermined (see above).

Reporting for specific materials, systems and methods

We require information from authors about some types of materials, experimental systems and methods used in many studies. Here, indicate whether each material, system or method listed is relevant to your study. If you are not sure if a list item applies to your research, read the appropriate section before selecting a response.

Materials & experimental systems

n/a	Involved in the study	
☐	☒	Antibodies
☒	☐	Eukaryotic cell lines
☒	☐	Palaeontology
☐	☒	Animals and other organisms
☒	☐	Human research participants
☒	☐	Clinical data

Methods

n/a	Involved in the study	
☒	☐	ChIP-seq
☒	☐	Flow cytometry
☒	☐	MRI-based neuroimaging

Antibodies

Antibodies used	All antibodies were from commercial suppliers (Cell Signaling Technology, unless otherwise stated) and used at a dilution of 1:1000 (unless otherwise stated). These include; Anti-IRβ antibody (4B8) rabbit monoclonal antibody (mAB) (Cat#3025), Anti-CD45 (Rat anti-mouse; used at 1:50 dilution for immunohistochemistry) from BD Biosciences (Cat#550539), Rabbit anti-α-amylase from Sigma-Aldrich used at 1:15,000 dilution (Cat#A8273), Anti-Akt1 (C73H10) rabbit mAb used at 1:14,000 (Cat#2938) Phospho-Akt (Ser473) used at 1:4,000 dilution (Cat#9271), Phospho-PFKFB2 (Ser483) rabbit mAb used at 1:5,000 dilution

	(Cat#13064), Anti-Actin mAB used at 1:100,000 dilution from Sigma-Aldrich (Cat#A3854-200UL), Anti-Cyclophilin-A from (Cat#2175), Immobilized Phospho-Akt Substrate mAB used at a dilution of 1:20 during immunioprecipitation (Cat#9646), Phospho-Akt Substrate mAb (HRP) from (Cat#6950), Phospho-(Ser/Thr) Akt Substrate Antibody (Cat#9611), PKM2 (D78A4) XP® Rabbit mAb (Cat#4053), Pyruvate Dehydrogenase Rabbit mAb (Cat#3205), Hexokinase I (C35C4) Rabbit mAb (Cat#2024), LDHA (C4B5) Rabbit mAb (Cat#3582), PKM1/2 (C103A3) Rabbit mAb (Cat#3190), PFKP (D4B2) Rabbit mAb (Cat#8164), Anti-rabbit IgG, HRP-linked Antibody (Cat#7074), Anti-IRS1 Rabbit mAB from Invitrogen (Cat#MA5-15068), Anti-IRS2 Mouse mAB from Millipore (Cat#MABS15), Anti-IRS2 Rabbit mAB from Abcam (Cat#ab13410), Anti-Phosphotyrosine Antibody clone 4G10 used at 1:8,000 from Sigma-Aldrich (Cat#05-321), Anti-rabbit IgG Antibody used at 1:5,000 dilution (Cat# 2729). Dynabeads Protein G for immunoprecipitation were from Invitrogen (Cat#10003D).These are listed along with their corresponding commercial supplier and catalogue numbers in the table in Supplementary Information.
Validation	All primary antibodies have been validated by their respective commercial supplier with citations to multiple published studies that further validate the antibodies that can be found on their respective websites.

Animals and other organisms

Policy information about studies involving animals; ARRIVE guidelines recommended for reporting animal research

Laboratory animals	All in vivo pancreatitis experiments were performed on male mice either PACIRKO mice, or their littermate control mice (IRlox/lox), and diabetic Ins2Akita mice, and their littermate control mice (C57BL/6 wildtype mice) at age 6-9 weeks. Similarly most in vitro-based experiments were also performed on cells/tissue isolated/harvested from PACIRKO vs IRlox/lox mice with some additional experiments performed on the cells/tissue isolated/harvested from Ela-CreER/+ or the genetic background C57BL/6 wildtype strain of mice. All mice are kept in individually ventilated cages with 12h light : 12h dark cycles maintained at 22°C +/- 2 °C and between 45 and 65 relative humidity with free access to food and water. These conditions are in accordance with the Codes of Practice for the care and accommodation of animals under section 21 of the Animals (Scientific Procedures) Act 1986 as amended in 2012 ("ASPA").
Wild animals	The study did not involve wild animals
Field-collected samples	The study did not involve samples taken from the field
Ethics oversight	All animal procedures and in vivo experiments were approved by the University of Michigan Institutional Animal Care and Use Committee (IACUC). The breeding of PACIRKO mice and feeder strains (Ela-CreER/+ and IRlox/lox) at the University of Manchester, which includes the administration of tamoxifen to induce induce insulin receptor deletion was approved by the Home Office Project licence (PPL number, P08B76E2B; PPL holder, Michael Simonson-Jackson).

Note that full information on the approval of the study protocol must also be provided in the manuscript.
