# Supplementary material for: Insulin protects acinar cells during pancreatitis by preserving glycolytic ATP supply to calcium pumps
Source: Nat Commun. 2021 Jul 19;12:4386. doi: 10.1038/s41467-021-24506-w (PMC8289871; doi:10.1038/s41467-021-24506-w)
Supplement: Supplementary file 3 — Source data [file 41467_2021_24506_MOESM3_ESM.pdf]

Reviewers' Comments:

Reviewer #1:
Remarks to the Author:
The study demonstrates the importance of insulin and insulin receptors on pancreatic acinar cells in reducing the severity of acute pancreatitis. This is an important finding. The primary phenomenon was clearly demonstrated, the authors provided convincing evidence of the cellular mechanism responsible for the insulin protection (increased efficiency of PMCA and consequent reduction in Ca2+ toxicity) and made a reasonable and systematic attempt to define the molecular mechanism. I have a few minor points:
The graphical abstract is somewhat overloaded and can be simplified.
The described effect of insulin on NAD(P)H fluorescence in cells treated by CCCP and IAA (in presence / absence of insulin receptors) is an interesting observation. It would be useful to discuss how this relates to expected ATP changes.
It would be useful to further discuss the observed high level of pPFKFB2 in PACIRKO mice.
This study provides sufficient evidence for the significant changes in the severity of acute pancreatitis but in the further studies (not this paper) it would be perhaps useful to characterise the effect on the early stages of pancreatic damage (e.g. pancreatic trypsin activity).

Reviewer #2:
Remarks to the Author:
There has been speculation as to the role of islet function and especially insulin secretion in acute and chronic pancreatitis, not least because of the close anatomical relationship between pancreatic endocrine and exocrine tissue, with arterial blood feeding first into islets and a portal circulation within the pancreas that then supplies acinar tissue. This study specifically addresses the potential role of insulin in pancreatic acinar cell injury driving acute pancreatitis. The authors provide substantial evidence obtained in a number of different and corroborative ways in multiple murine models for a direct effect of insulin via insulin receptors in driving anaerobic glycolysis that contributes to pancreatic acinar cellular ATP supply, known to be critically impared by mitochondrial injury in acute pancreatitis. In this study of Bruce et al the Pancreatic Acinar Conditional Insulin Receptor Knockout (PACIRKO) model is particularly informative. As a result of the action of insulin, shown to be through the insulin receptor of pancreatic acinar cells, there is protection from the intracellular calcium overload resulting from pancreatitis toxins in pancreatic acinar cells. There is corresponding protection in vivo, shown in two representative models of acute pancreatitis that are thoroughly and appropriately characterised. While it would have been desirable to have evidence of systemic protection e.g. lung injury this is not necessary for the present manuscript as the pancreatic injury of acute pancreatitis drives systemic injury, and the extent of pancreatic injury was such that it can reasonably be assumed that systemic injury will simply reflect the findings made. Similarly it would have been desirable to have data for such mechanisms in and potential for protection of human pancreatic acinar cells, but it is not necessary for the present manuscript, but could be the content of a further manuscript.
There are several textual issues:
1) line 58 would read better as 'reduced insulin sensitivity (type-2 diabetes)' not 'reduced insulin effectivenes (spelling) (type-1 diabetes)'
2) line 240 remove 'similar' as this appears later in the sentence
3) line 280 there are a number of other mechanisms likely to account for the association of a raised mortality in chronic pancreatitis with diabetes mellitus
4) line 282 and 283 switch to past tense inappropriate
5) line 316 'collatoral' (spelling)
6) line 336 'priveleged' (spelling)
7) line 338 'globl' (spelling)
8) line 361 suggest (and likely insulin receptors within acinar cells)
The 'conclusion' is repetitive and first section unnecessary so it is suggested that lines 387-391 are removed.
Concerning the figures:
1) figure 3biv the ordinate label should be Relative 18S rRNA, if the legend is correct
2) figure 4biv the ordinate label should be Relative 18S rRNA, if the legend is correct

3) figure 6g POA concentration should be 0 microM in the first PACIRKO column, if the legend is correct
Reviewed by Professor Robert Sutton

Reviewer #3:
Remarks to the Author:
Acute pancreatitis (AP) is serious inflammatory disease of the pancreas. Accumulating evidence links diabetes with severity of AP, suggesting that endogenous insulin may be protective. In the present study, the authors investigated this putative protective effect of insulin during cellular and in vivo models of AP in diabetic mice (Ins2Akita) and Pancreatric Acinar cell-specific Conditional Insulin Receptor Knock Out mice (PACIRKO). Caerulein and palmitoleic acid (POA)/ethanol-induced pancreatitis was more severe in both Ins2Akita and PACIRKO versus control mice, suggesting that endogenous insulin directly protects acinar cells in vivo. In isolated pancreatic acinar cells, insulin induced Akt-mediated phosphorylation of 6-phosphofructo-2-kinase/fructose-2,6-biphosphatase 2 (PFKFB2) which upregulated glycolysis thereby preventing POA-induced ATP depletion, inhibition of the ATP-dependent plasma membrane Ca2+ ATPase (PMCA) and cytotoxic Ca2+ overload. The authors concluded that these data provided the first mechanistic link between diabetes and severity of AP and suggested that phosphorylation of PFKFB2 may represent a novel therapeutic strategy for treatment of AP.

Major Criticisms

1. There was a high basal Akt and PFKFB2 phosphorylation in untreated acinar cells from PACIRKO mice compared to acinar cells from IRlox/lox mice. There was no further increase in Akt or PFKFB2 phosphorylation following insulin treatment of acinar cells from PACIRKO mice. The authors speculate that the loss of insulin receptors was responsible for this insulin insensitivity. This reviewer requests the authors to examine the expression and phosphorylation levels of insulin receptor substrates, such as IRS-1 and IRS-2, because there are another up-stream signals for these molecules. For example, incretin, such as GLP-1, increases cAMP, thereby stimulating phosphorylation of CREB and up-regulating IRS-2. Information on these molecules may be needed to access the high basal Akt level in untreated acinar cells from PACIRKO mice.
2. In isolated pancreatic acinar cells, insulin induced Akt-mediated phosphorylation of 6-phosphofructo-2-kinase/fructose-2,6-biphosphatase 2 (PFKFB2) which upregulated glycolysis thereby preventing POA-induced ATP depletion, inhibition of the ATP-dependent plasma membrane Ca2+ ATPase (PMCA) and cytotoxic Ca2+ overload. The authors speculate that phosphorylation of PFKFB2 acts as a “volume control” for glycolytic flux and thus ATP production and is thus likely to be the major molecular mechanism for the protective effects of insulin during pancreatitis. Then, the authors may provide with the results on glycolytic flux in acinar cells under normal and AP conditions.
3. In the present study, the authors characterize mouse models whose insulin signaling is impaired in acinar cells. On the other hand, there are many models whose insulin signaling may be up-regulated due to hyperinsulinemia to compensate for systemic insulin resistance. Given the role of insulin signaling in acinar cells, such models may be protected against caerulein and POA/ethanol-induced pancreatitis. Is this true?
4. The authors propose that insulin infusion with the aim of reducing pancreatic injury may prove effective for the treatment of severe AP, as long as there is tight moment-to-moment glycaemic control (hyperinsulinaemic euglycaemic clamp). This reviewer asks the authors to examine the amount of infused insulin to sufficiently reduce pancreatic injury. Is it possible to protect against pancreatic injury and simultaneously avoid hypoglycemia?

Authors Responses to Reviewers

Reviewer #1 (Remarks to the Author):
The study demonstrates the importance of insulin and insulin receptors on pancreatic acinar cells in reducing the severity of acute pancreatitis. This is an important finding. The primary phenomenon was clearly demonstrated, the authors provided convincing evidence of the cellular mechanism responsible for the insulin protection (increased efficiency of PMCA and consequent reduction in Ca2+ toxicity) and made a reasonable and systematic attempt to define the molecular mechanism.

Authors Response: We would like to thank reviewer #1 for these comments and are pleased that they found merit in our study

Minor points*:*
The graphical abstract is somewhat overloaded and can be simplified.

Authors Response: This has now been modified by removing the additional data and simplifying the cartoon

The described effect of insulin on NAD(P)H fluorescence in cells treated by CCCP and IAA (in presence / absence of insulin receptors) is an interesting observation. It would be useful to discuss how this relates to expected ATP changes.

Authors Response: ATP was actually measured directly using the firefly luciferase assay, which is summarised in Figure 7a. These data show that insulin causes a rightward shift in the palmitoleic acid (POA) concentration-ATP response curve. This reaffirms the notion that insulin preserves ATP production and thus prevents POA-induced ATP depletion. However, in the context of the NADH autofluorescence experiments we used CCCP-induced NADH depletion as a surrogate for mitochondrial bioenergetics and IAA-induced NADH depletion as a surrogate for glycolytic bioenergetics. This is because CCCP is a protonophore and mitochondrial uncoupler that dissipates the mitochondrial proton gradient and thus mitochondrial membrane potential which in turn reduces the driving force for ATP depletion by the ATP synthase. Hence, CCCP is described as a mitochondrial uncoupler, as it uncouples ATP synthesis from the electron transport chain/oxygen consumption. This means that CCCP causes the electron chain and oxygen consumption (respiration) to “work in overdrive” at a maximum rate in an attempt to maintain the proton gradient against this futile cycle of maintaining ATP synthesis. This in turn causes mitochondrial NADH consumption (derived from Krebs cycle intermediates) to “fuel” the electron transport chain and leads to eventual mitochondrial NADH depletion. Therefore, CCCP-induced NADH depletion could be described as an indirect measure of mitochondrial bioenergetics and thus ATP production. IAA is an inhibitor of GAPDH, which is the major source of glycolytic NADH. Therefore, IAA-induced NADH depletion could be described as a measure of glycolytic bioenergetics and thus ATP production. These data show that by reducing CCCP-induced NADH depletion and increasing IAA-induced NADH depletion, insulin is effectively inducing a shift from mitochondrial bioenergetics towards glycolytic bioenergetics, which would further explain the rightward shift in the POA concentration ATP response curve to the right (Figure 7a). We have now added a further explanation of this in the revised manuscript (line 193-203, page 8).

However, we accept that NADH autofluorescence, whilst a convenient surrogate for mitochondrial vs glycolytic bioenergetics, is not a direct measure of ATP and indeed may be affected by numerous other factors that affect NADH and is confounded by NAD(P)H autofluorescence. Therefore, we further investigated whether this insulin-induced switch in mitochondrial vs glycolytic bioenergetics could be translated to ATP. This was achieved using the same experimental paradigm but instead cells were loaded with magnesium green (MgGreen), to assess ATP depletion. Since most cellular ATP exists as MgATP, any ATP depletion will cause a corresponding increase in free Mg^{2+}, which is detected by MgGreen.

Although this is still not a direct measure of ATP concentration per se, it is a much closer representation of ATP depletion that can be correlated with NADH autofluorescence. These experiments essentially showed the same phenomena; insulin caused a decrease in CCCP-induced increase in MgGreen fluorescence (ATP depletion) and increase in IAA-induced increase in MgGreen fluorescence (ATP depletion). This further supports the notion that insulin switched metabolism from mitochondria towards glycolytic ATP production that was sufficient to maintain cellular ATP even in the face of POA-induced impaired mitochondrial metabolism. These new data have now been included in Supplementary Information (Methods, line 164-175, page 6-7; Results, line 269-284; Figure S6) and described in the Results (line 213-227, page 8-9).
Its also worth noting in the context of these experiments that our previous studies in rat pancreatic acinar cells (ref 5, Mankad *et al* 2012 & ref 6, Samad, *et al* 2014) show that;
1-Insulin prevented POA-induced MgGreen fluorescence in isolated pancreatic acinar cells
2-The insulin switch from mitochondrial metabolism towards glycolysis also translated to PMCA activity (*in situ* Ca^{2+} clearance assay). Insulin markedly reduced the CCCP-induced inhibition of PMCA activity but potentiated the bromopyruvate (glycolytic inhibitor)-induced inhibition of PMCA activity. Again this is consistent with a switch in metabolism which not only translates to ATP production but also PMCA activity.

It would be useful to further discuss the observed high level of pPFKFB2 in PACIRKO mice.

Authors Response: We have now investigated this further and provide new data showing that the high basal phosphorylation of PFKFB2 in PACIRKO mouse acinar cells is most likely due to an increased expression and phosphorylation of IRS1 and IRS2 (Figure S9), upstream of activation of PI3K/Akt. This will inevitably lead to a higher basal phosphorylation of Akt and consequently PFKFB2. The mechanism for this upregulation of IRS1/IRS2 remains unclear but may reflect some kind of compensatory mechanism in response to deletion of the insulin receptor (IR). However, it is important to note that insulin treatment of PACIRKO mouse acinar cells failed to increase phosphorylation of IRS1/IRS2 (Figure S9), Akt (Figure 8a*i*) or PFKFB2 (Figure 8a*iii*), further supporting the notion that IR deletion prevents insulin-mediated downstream signalling and thus protection against cellular injury. On the other hand, insulin treatment of $IR^{lox/lox}$ mouse acinar cells caused IRS1 (Figure S9b*i*) , Akt (Figure 8a*i*) and PFKFB2 phosphorylation (Figure 8a*iii*), as one would expect with normal IR expression. These new data are now shown in Figure S9 and described in the Supplementary Results (line 303-315, page 13-14).

This study provides sufficient evidence for the significant changes in the severity of acute pancreatitis but in the further studies (not this paper) it would be perhaps useful to characterise the effect on the early stages of pancreatic damage (e.g. pancreatic trypsin activity).

Authors Response: We agree that additional measures of pancreatic injury in addition to distant organ injury would further strengthen these data. However, due to the logistical issues of the COVID-19 pandemic and in the interest of reducing animal use, it was decided that these experiments could not justify such an incremental increase in knowledge.

Reviewer #2 (Remarks to the Author):
There has been speculation as to the role of islet function and especially insulin secretion in acute and chronic pancreatitis, not least because of the close anatomical relationship between pancreatic endocrine and exocrine tissue, with arterial blood feeding first into islets and a portal circulation within the pancreas that then supplies acinar tissue. This study specifically addresses the potential role of insulin in pancreatic acinar cell injury driving acute pancreatitis. The authors provide substantial evidence obtained in a number of different and corroborative ways in multiple murine models for a direct effect of insulin via insulin receptors in driving anaerobic glycolysis that contributes to pancreatic acinar cellular ATP supply, known to be critically impared by mitochondrial injury in acute pancreatitis. In this

study of Bruce et al the Pancreatic Acinar Conditional Insulin Receptor Knockout (PACIRKO) model is particularly informative. As a result of the action of insulin, shown to be through the insulin receptor of pancreatic acinar cells, there is protection from the intracellular calcium overload resulting from pancreatitis toxins in pancreatic acinar cells. There is corresponding protection in vivo, shown in two representative models of acute pancreatitis that are thoroughly and appropriately characterised. While it would have been desirable to have evidence of systemic protection e.g. lung injury this is not necessary for the present manuscript as the pancreatic injury of acute pancreatitis drives systemic injury, and the extent of pancreatic injury was such that it can reasonably be assumed that systemic injury will simply reflect the findings made. Similarly it would have been desirable to have data for such mechanisms in and potential for protection of human pancreatic acinar cells, but it is not necessary for the present manuscript, but could be the content of a further manuscript.

Authors Response: We would like to thank reviewer #2 for their positive and pragmatic comments. On reflection, we also agree that additional measures of both pancreatic injury in addition to distant organ injury would further strengthen our data. However, due to the logistical issues of the COVID-19 pandemic and in the interest of reducing animal use, we decided that these experiments could not justify such a small incremental increase in knowledge.

There are several textual issues:
1) line 58 would read better as 'reduced insulin sensitivity (type-2 diabetes)' not 'reduced insulin effectivenes (spelling) (type-1 diabetes)'
2) line 240 remove 'similar' as this appears later in the sentence
3) line 280 there are a number of other mechanisms likely to account for the association of a raised mortality in chronic pancreatitis with diabetes mellitus
4) line 282 and 283 switch to past tense inappropriate
5) line 316 'collatoral' (spelling)
6) line 336 'priveleged' (spelling)
7) line 338 'globl' (spelling)
8) line 361 suggest (and likely insulin receptors within acinar cells)
The 'conclusion' is repetitive and first section unnecessary so it is suggested that lines 387-391 are removed.
Concerning the figures:
1) figure 3biv the ordinate label should be Relative 18S rRNA, if the legend is correct
2) figure 4biv the ordinate label should be Relative 18S rRNA, if the legend is correct
3) figure 6g POA concentration should be 0 microM in the first PACIRKO column, if the legend is correct

Authors Response: We apologise for these minor errors, which have now been corrected

Reviewer #3 (Remarks to the Author):
Acute pancreatitis (AP) is serious inflammatory disease of the pancreas. Accumulating evidence links diabetes with severity of AP, suggesting that endogenous insulin may be protective. In the present study, the authors investigated this putative protective effect of insulin during cellular and in vivo models of AP in diabetic mice (Ins2Akita) and Pancreatric Acinar cell-specific Conditional Insulin Receptor Knock Out mice (PACIRKO). Caerulein and palmitoleic acid (POA)/ethanol-induced pancreatitis was more severe in both Ins2Akita and PACIRKO versus control mice, suggesting that endogenous insulin directly protects acinar cells in vivo. In isolated pancreatic acinar cells, insulin induced Akt-mediated phosphorylation of 6-phosphofructo-2-kinase/fructose-2,6-biphosphatase 2 (PFKFB2) which upregulated glycolysis thereby preventing POA-induced ATP depletion, inhibition of the ATP-dependent plasma membrane Ca2+ ATPase (PMCA) and cytotoxic Ca2+ overload. The authors concluded that these data provided the first mechanistic link between diabetes and

severity of AP and suggested that phosphorylation of PFKFB2 may represent a novel therapeutic strategy for treatment of AP.

Major Criticisms

1. There was a high basal Akt and PFKFB2 phosphorylation in untreated acinar cells from PACIRKO mice compared to acinar cells from IRlox/lox mice. There was no further increase in Akt or PFKFB2 phosphorylation following insulin treatment of acinar cells from PACIRKO mice. The authors speculate that the loss of insulin receptors was responsible for this insulin insensitivity. This reviewer requests the authors to examine the expression and phosphorylation levels of insulin receptor substrates, such as IRS-1 and IRS-2, because there are other up-stream signals for these molecules. For example, incretin, such as GLP-1, increases cAMP, thereby stimulating phosphorylation of CREB and up-regulating IRS-2. Information on these molecules may be needed to access the high basal Akt level in untreated acinar cells from PACIRKO mice.

Authors Response: We thank Reviewer #3 for these insightful comments and suggestions for additional experiments. In an attempt to address the issue of the high basal Akt/PFKFB2 phosphorylation in PACIRKO mice, we investigated the expression of IRS1 and IRS2 in PACIRKO vs $IR^{lox/lox}$ mice by western blotting using specific antibodies for IRS1 and IRS2. Although IRS1 and IRS2 can be phosphorylated by numerous kinases, insulin activation of insulin receptors leads to tyrosine kinase phosphorylation of IRS1/2, therefore, we reasoned that the tyrosine phosphorylation status of IRS1/IRS2 would be the most important to investigate. We therefore co-immunoprecipitated either IRS1 or IRS2 and western blotted using the Anti-phosphotyrosine antibody (clone 4G10, Sigma) to detect the general tyrosine phosphorylation status of these proteins following treatment with and without insulin treatment. Results show that as predicted by Reviewer #3, both IRS1 and IRS2 were markedly over-expressed (Figure S9) and hyperphosphorylated (Figure S9b*i* & Figure S9b*ii*) in PACIRKO mouse acinar cells, most likely responsible for the high basal phosphorylation of both downstream Akt and consequently PFKFB2. The mechanism for this upregulation of IRS1/IRS2 remains unclear but may reflect some kind of compensatory mechanism in response to deletion of the insulin receptor (IR). However, it is important to note that insulin treatment of PACIRKO mouse acinar cells failed to increase phosphorylation of IRS1/IRS2 (Figure S9b*i*), Akt (Figure 8a*i*) or PFKFB2 (Figure 8a*iii*), further supporting the notion that IR deletion prevents insulin-mediated downstream signalling and thus protection against cellular injury. On the other hand, insulin treatment of $IR^{lox/lox}$ mouse acinar cells caused IRS1 (Figure S9b*i*) , Akt (Figure 8a*i*) and PFKFB2 phosphorylation (Figure 8a*iii*), as one would expect with normal IR expression.

2. In isolated pancreatic acinar cells, insulin induced Akt-mediated phosphorylation of 6-phosphofructo-2-kinase/fructose-2,6-biphosphatase 2 (PFKFB2) which upregulated glycolysis thereby preventing POA-induced ATP depletion, inhibition of the ATP-dependent plasma membrane Ca2+ ATPase (PMCA) and cytotoxic Ca2+ overload. The authors speculate that phosphorylation of PFKFB2 acts as a "volume control" for glycolytic flux and thus ATP production and is thus likely to be the major molecular mechanism for the protective effects of insulin during pancreatitis. Then, the authors may provide with the results on glycolytic flux in acinar cells under normal and AP conditions.

Authors Response: We thank Reviewer #3 for these insightful comments and suggestions for additional experiments which we now believe further strengthen our data and hypothesis. Glycolysis was assessed in isolated mouse (C57BL/6) pancreatic acinar cells in response to the pancreatitis-inducing agent POA (30 µM) with or without insulin (10 nM) treatment using the pH-Xtra Glycolysis Assay (Agilent; see Supplementary Methods, line 176-196, page 7). This measures extracellular acidification rate (ECAR), due primarily to lactic acid efflux and thus is a convenient measure of glycolytic flux. The pH-Xtra assay utilises a dual-read ratiometric time-resolved fluorescence lifetime measurement (Figure S7a), which is converted to extracellular pH (Figure S7b) and $[H^+]$, using the MARS data analysis software (see Supplementary Methods, line 176-196, page 7) from which ECAR can be determined

and normalised to control (Figure S7c, mean data). Results show that insulin (10 nM) alone caused a marginal, but insignificant increase in ECAR (127 ± 14 % of untreated control). However, POA (30 μM) markedly reduced ECAR to 54 ± 6 % of untreated control cells (Figure S7), which was restored to similar levels of control (96 ± 5 %) by pre-incubation with insulin (10 nM). These data further support the notion that insulin maintains glycolytic flux and thus ATP production to fuel the PMCA, even in the face of POA-induced metabolic crisis. These new data are now described in the Results section (line 228-241, page 9) and Supplementary Methods (line 176-196, page 7) and Supplementary Results (line 285-302, page 13).

3. In the present study, the authors characterize mouse models whose insulin signaling is impaired in acinar cells. On the other hand, there are many models whose insulin signaling may be up-regulated due to hyperinsulinemia to compensate for systemic insulin resistance. Given the role of insulin signaling in acinar cells, such models may be protected against caerulein and POA/ethanol-induced pancreatitis. Is this true?

Authors Response: Intuitively it would make sense to combine experimentally-induced pancreatitis in mouse models that are hyperinsulinaemic to determine if there is any protection, as our data would suggest. Indeed there are numerous type 2 diabetes mouse models that in the early phase of the disease are hyperinsulinaemic, in response to the insulin resistance and a compensatory pancreatic β-cell hypertrophy/hyperplasia, but then become hypoinsulinaemic in the later course of the disease and the eventual loss of β-cells. These include B6-ob/ob, NON/ShiLtJ, KK-A^{Y} and the polygenic mouse lines, NONcNZO1O/LtJ and TALLYHO/JngJ mice. As far as we are aware there have been no studies in which experimental pancreatitis has been induced in these mice. However, despite the initial hyperinsulinaemia, type 2 diabetes in mouse models or in patients is also accompanied by profound hyperglycaemia and frequently hyperlipidaemia, both of which make pancreatitis worse. This makes these models unsuitable for studying the link between insulin and pancreatitis due to the numerous confounding effects hyperglycaemia and hyperlipidaemia that would make it impossible to dissect the mechanism.

The K_{ATP} knockout or loss of function mutant transgenic mice represent models of congenital hyperinsulinism, are hyperinsulinaemic without the corresponding hyperglycaemia or hyperlipidaemia observed in type-2 diabetes models. These may therefore represent better models for studying the protective effects of insulin during pancreatitis. However, it would take several months/years to acquire, breed, obtain Home Office approval and to complete pancreatitis experiments in any of the above hyperinsulinaemic mouse models. This would therefore be impossible in the time available for revision, even without a global pandemic, and is thus beyond the scope of this study. Moreover, even if successful, these experiments would only add to complement our existing data and are not critical in proving or disproving our over-arching hypothesis which raises questions about the ethical justification for such experiments.

4. The authors propose that insulin infusion with the aim of reducing pancreatic injury may prove effective for the treatment of severe AP, as long as there is tight moment-to-moment glycaemic control (hyperinsulinaemic euglycaemic clamp). This reviewer asks the authors to examine the amount of infused insulin to sufficiently reduce pancreatic injury. Is it possible to protect against pancreatic injury and simultaneously avoid hypoglycaemia?

Authors Response: We thank Reviewer #3 for these insightful comments and suggestion for additional experiments. We have now included new data obtained in collaboration with the Animal Phenotyping Core Facility at the University of Michigan. This involved combining caerulein-infusion induced acute pancreatitis (50 μg/kg/h) with the hyperinsulinaemic euglycasemic clamp. This enabled infusion of high dose exogenous insulin (12 mU/kg/min) while maintaining tight glycaemic control by adjusting glucose infusion rate according to

blood glucose which was monitored every 10 minutes. Specifically, this involved catheterisation of the jugular vein and carotid artery under anaesthesia. This allowed the cannulation of the arterial line for the administration of caerulein, insulin and glucose by continuous infusion using automatic syringes and mini-pumps (SEE METHODS). Following surgery, mice were allowed to recover and acclimatize for 5 days prior to experimentation and were housed individually and their body weight monitored daily.

Initial caerulein infusion experiments sought to determine the optimum caerulein administration by comparing continuous low dose infusion (10 µg/kg/h), high dose infusion (50 µg/kg/h) and hourly bolus caerulein (50 µg/kg) injection via the IV line (to mimic the conventional IP injections used in previous well established models, Figure 1and 3). Blood samples were collected (via sampling of the IV line) at time 0, 1h, 3h and 5 hour of the experiment for assessment of plasma amylase as an early readout of pancreatic injury. This revealed that continuous high dose caerulein infusion (50 µg/kg/h) induced the greatest increase in plasma amylase at 5 hours (23 ± 1.4 Uml-1, n=4, Figure 9bi) compared to low dose (10 µg/kg) insulin infusion (10 ± 0.6 Uml-1, n=4, Figure 9bi) or hourly bolus injections (15 ± 1.7 Uml-1, n=4, Figure 9bi). Moreover, this high dose insulin infusion (50 µg/kg/h) induced a similar increase in plasma amylase to that induced by 8 hourly IP injections of caerulein over 2 days (23 ± 2.0, n=7, Figure S1f). This suggests that this high dose caerulein infusion (50 µg/kg/h) was sufficient to induce pancreatic injury at 5 hours and was therefore taken forward for the combined caerulein infusion and hyperinsulinaemic euglycemic clamp experiments.

Blood glucose was reasonably well maintained during each experiment (insulin alone, Figure 9ai; combined caerulein/insulin, Figure 9aii; caerulein alone, Figure 9aiii and saline control, Figure 9aiv), with the exception of the spike in blood glucose when glucose infusion rate was increased to accommodate the onset of insulin infusion (Figure 9ai and Figure 9aii). This suggests that during high dose insulin infusion, tight glycaemic control can be maintained by close glucose monitoring and adjusting glucose infusion rate accordingly.

Measurement of plasma amylase at 5 hours revealed that the caerulein infusion-induced an increase in plasma amylase (14 ± 1.3 Uml-1, n=6, Figure 9bii) that was significantly reduced by high dose insulin infusion initiated 2 hours after the onset of caerulein infusion (9 ± 0.6 Uml-1, n=6, Figure 9bii, $p<0.05$). However, insulin infusion alone had no effect (5 ± 0.5 Uml-1, n=6, Figure 9bii) compared to saline control (5.6 ± 0.3 Uml-1, n=7, Figure 9bii). These data suggest that exogenous insulin infusion with tight glycaemic control may reduce early pancreatic injury associated with acute pancreatitis.

Reviewers' Comments:

Reviewer #1:
Remarks to the Author:
All my specific comments have been addressed satisfactorily. Additional experiments involving MgGreen measurements of ATP levels provided valuable information and strengthened the conclusions of the study.
Very minor. Please correct "glycolytoc" on line 282 in Supplementary Information.

Reviewer #2:
Remarks to the Author:
This work of Bruce and colleagues is original, well-conducted and important as it identifies novel effects of insulin on pancreatic acinar cells that are beneficial in experimental acute pancreatitis and may have application as a new therapy for acute pancreatitis, which currently lacks specific targeted therapy.

Reviewer #3:
Remarks to the Author:
None
